# The Meta-Dynamic Nature of Consciousness

**DOI:** 10.3390/e22121433

**Published:** 2020-12-18

**Authors:** John A. Barnden

**Affiliations:** School of Computer Science, University of Birmingham, Birmingham B15 2TT, UK; jabarnden@btinternet.com; Tel.: +44-0-121-440-5677

**Keywords:** consciousness, meta-causation, pre-reflective self-consciousness, physicalism, causal productivity, dynamism, laws of nature, laws of physics, temporal non-locality

## Abstract

How, if at all, consciousness can be part of the physical universe remains a baffling problem. This article outlines a new, developing philosophical theory of how it could do so, and offers a preliminary mathematical formulation of a physical grounding for key aspects of the theory. Because the philosophical side has radical elements, so does the physical-theory side. The philosophical side is radical, first, in proposing that the productivity or dynamism in the universe that many believe to be responsible for its systematic regularities is actually itself a physical constituent of the universe, along with more familiar entities. Indeed, it proposes that instances of dynamism can themselves take part in physical interactions with other entities, this interaction then being “meta-dynamism” (a type of meta-causation). Secondly, the theory is radical, and unique, in arguing that consciousness is necessarily partly constituted of meta-dynamic auto-sensitivity, in other words it must react via meta-dynamism to its own dynamism, and also in conjecturing that some specific form of this sensitivity is sufficient for and indeed constitutive of consciousness. The article proposes a way for physical laws to be modified to accommodate meta-dynamism, via the radical step of including elements that explicitly refer to dynamism itself. Additionally, laws become, explicitly, temporally non-local in referring directly to quantity values holding at times prior to a given instant of application of the law. The approach therefore implicitly brings in considerations about what information determines states. Because of the temporal non-locality, and also because of the deep connections between dynamism and time-flow, the approach also implicitly connects to the topic of entropy insofar as this is related to time.

## 1. Introduction: A Theory and Its Philosophical and Physical Sides

This article is about the development of a philosophically and physically distinctive theory, MDyn, of (phenomenal) consciousness, and a preliminary mathematical couching of it. I will normally suppress the “phenomenal” qualifier when mentioning consciousness—in this article, consciousness will always be a matter of what it is like to be in the state. Moreover, “experience” will always mean phenomenally conscious experience.

MDyn can heuristically be thought of as having a *philosophical side*, a *physical-theory side* and a *postulate of physically reified dynamism* (or *reification postulate* for short) that bridges between the two sides. The philosophical side rests on: an anti-Humean view of the universe whereby its regularities arise from underlying real “dynamism” or “pushing-forwards”; on an assumption about a type of reflexivity in consciousness, whereby a conscious process is physically, sensitive to its own dynamism; and on an argument that this sensitivity takes the form of “*meta-dynamism*”, which is dynamism that acts directly on dynamism itself. The reification postulate says that dynamism is not just metaphysically real but is a first-class citizen of the physical universe. The physical-theory side develops a way of accommodating the thus-reified dynamism, including meta-dynamism, in physical laws and system equations, and therefore in particular a way of mathematically describing conscious processes, which are constituted at base of the mentioned reflexivity realized as “auto-meta-dynamism”. The following subsections of this Introduction outline these components of MDyn in further detail.

The philosophical side, and to a small extent the physical-theory side, have been in development for some time [1,2,3,4], but most of the physical-theory side is new. (It was presaged in my *Models of Consciousness* conference talk in 2019 [4], but many concepts and most details have changed since then.) The physical-theory side is, therefore, more provisional and schematic than the philosophical side, leaving many alternative lines of development open. However, developing the physical-theory side has already been valuable, not just for its own sake but also for catalyzing certain important, new developments on the philosophical side, incorporated for the first time in the present article.

### 1.1. Modelling Phenomenality as Such

MDyn is a physicalist theory of *phenomenality as such*: it proposes a way in which the sheer phenomenality of consciousness can exist as an aspect of the physical world.

By saying MDyn is a theory of phenomenality as such I mean to stress the following: It is not a model of consciousness in the more ambitious sense of specifying the nature of some type of complete conscious, cognitive system, such as a conscious neural network or a conscious program running in a computer, or a major component of such a system, such as a global workspace [5,6]. Nor is it a model of how conscious perceiving, acting or thinking proceeds. It just seeks to provide for the general phenomenality that is possessed by all occurrently conscious systems. It makes fundamental claims about the core physical nature of any occurrence of consciousness, no matter how that core nature is woven into the rest of some more or less elaborate, conscious cognitive system. The article does provide mathematical equations for a toy, illustrative simple physical system that could be conscious in some very basic way according to the theory. But this small system would need to be developed further to become at all close to a realistic system that might exist in even a simple creature, robot, etc.

Amongst current efforts to model consciousness in a technically detailed theory, MDyn finds most fellow-feeling with IIT [7], in that it seeks to go from basic considerations about the nature of phenomenality to physical postulates, and ends up with a theory that is not centrally concerned with the overall activities of a complete cognitive system, but rather with the conditions under which it has phenomenality at all. A further very general similarity to IIT is that it focusses on causal structure. However, it radically departs from IIT and from virtually all other theories because it introduces a fundamentally new type of causation (new for consciousness theory, that is) as the very basis for consciousness. (Section 5.4.4 will discuss a possible historical precursor in the form of Fichte’s theory [8] from late 18th century, but even here only under a suitable, speculative interpretation.)

As we will see in Section 2.8 and Section 5.2, MDyn raises in a natural way the possibility of a very pure episode of conscious experience that is little more than an experience of being aware of its own continued existence. This connects to notions of content-less, pure, core and minimal consciousness that a variety of consciousness researchers have entertained or discussed [8,9,10,11,12,13,14,15,16,17,18] MDyn’s interest in such forms of consciousness is in the service, however, of its ultimate ambition to explain the phenomenality of everyday, complex consciousness, not just such pure consciousness.

MDyn leaves it open whether consciousness is *strictly on/off* (i.e., it is either present or absent, with no gradations, in a given system at a particular time), *fully graded* (i.e., it comes in degrees that can be anywhere in some interval up from zero), or *partially graded* (i.e., a combination of on/off and graded, in that the conscious degree is either greater than or equal to some fixed positive value; see also [19] for this suggestion). My own suggestion is that consciousness is partially graded, and that there may be different, independent senses in which it is graded, but nothing in the article depends on these suggestions.

### 1.2. Non-Egoic Phenomenality

MDyn encourages the idea that conscious experience can be *non-egoic* (non-egological) (see, e.g., [14,20,21], and [22] (p. 177–179) on Sartre). That is, MDyn encourages the claim that conscious experience does not need to involve a “self” or “I” which is, or is perceived by the experience as being, the entity that is having the experience and is differentiated from the experience itself. The experience intrinsically experiences *itself* according to MDyn, as will become apparent below, but may not experience *a self* other than the whole of itself. Rather, selves may be (real or illusory) aspects only of relatively rich or advanced forms of conscious system.

Moreover, in MDyn, experiences can in principle be entirely evanescent and disconnected, both synchronically and diachronically. Synchronically: MDyn leaves it open that a unified system could at some moment have several different experiences that have no cross-experiencing and do not form a unified experience, Diachronically: one and the same physical system may have experiences at different times where later ones have no experience of or information about earlier ones.

Because of the non-egoic possibility, it is salutary to abandon the prefix “self” in terms such as “self-conscious” in favour of the prefix “auto”. Accordingly, talk of “*auto-consciousness*” is clearer than talk of “self-consciousness”.

### 1.3. The Crux of the Philosophical Side

The nub of the philosophical side of MDyn, and the central claim of the whole theory, is that *phenomenality as such consists of a suitable type and arrangement of physical “meta-dynamism”.* Dynamism can intuitively be thought of as causation, and hence meta-dynamism as meta-causation. Meta-causation in the sense used here is where cause and/or effect are themselves instances of causation. (Unfortunately there are some very different senses for the term “meta-causation”, and some of these are more widely discussed, such as downwards causation. I say more on this in Appendix A.1.) We will be considering causation (dynamism) at a basic physical level. However, it may help the reader heuristically to see an intuitive example of (supposed) meta-causation at an everyday, common-sense level. Consider the sentence “John’s causing the car to run out of fuel caused Bill to laugh at him”. Here the first-mentioned causing is portrayed as the cause in the secondly mentioned causing. A causing can also be portrayed as a meta-causal effect, as in “John’s carelessness caused him to cause the car ro run out of fuel”, or more idiomatically, “Carelessness made John make the car run out of fuel”. (However, it is *not* proposed that these examples actually succeed in describing real, physical meta-causation in the sense of this article.)

More specifically, the philosophical side of MDyn says that, in any episode of conscious experiencing, *the causing that has arisen so far at a given point in the experiencing directly and meta-causally affects the later course of the experiencing.* To turn this around: the experiencing is constantly, directly sensitive through meta-causation to its own prior internal causation, over some time interval up to the present time. It is meta-causally, unmediatedly sensitive to its own recent causal history, i.e., to the very causation that holds the conscious process together as a process. We can thus say that, according to MDyn, consciousness is a matter of “auto-meta-causation”.

As discussed further below, meta-causation that is conceptually similar to that proposed in this paper has some presence in the philosophy of causation. But this presence is relatively tiny, as Kovacs [23] notes. However, furthermore, it has apparently not been previously proposed that consciousness is a matter of meta-causation. This makes MDyn doubly distinctive as regards how it involves causation.

The “causation” considered in MDyn is *dynamism* at a basic physical level. This is the sheer pushing-forward of the physical world everywhere and at all times. It is not just the fact that basic physical states (electrical fields mass distributions, spacetime curvatures, etc.) evolve, but is the necessitation of continued evolution exerted by the history so far of (some parts of) the world. Put another way, it is the causal interaction over time of aspects of the physical world with each other, that interaction considered as ontologically real in its own right, not just part of a view we have of the world. The claim that such dynamism is ontologically real at all, as opposed to the world consisting *just* of familiar sorts of material states at different place and times, puts MDyn in the “anti-Humean” camp within philosophy, as explained further below. The basic anti-Humean idea that the regular, apparently law-governed self-consistent unfolding of the universe is not there through pure arbitrariness—it is not just a coincidence that regularities in, for example, how electrons and magnetic fields behave, are the same here and now as those a second ago and an inch away. Some anti-Humeans have claimed that, instead, there is some sort of productiveness that, so to speak, pushes the universe forward in a systematic and consistent way. In short, things happen because they are *made to* happen. Some have called this pushing-forward the [metaphysical or causal] “oomph” in the universe. (On this concept, see [24,25,26] for suggestions and discussion. (On the question of anti-Humean stances in general, I have been particularly swayed by the physics-informed considerations of Maudlin [27]. However, he does not go so far as the radical reification of dynamism in MDyn.)

MDyn introduces a particularly radical form of anti-Humeanism, in two different respects. One is the *physical* reification of dynamism in the next subsection. The other is the inclusion of *meta*-dynamism. In parallel with the minority status of meta-causation in the theory of causation in general, meta-dynamism is rarely entertained in anti-Humeanism, even implicitly (an exception will be mentioned below). That is, it is rare for it to be entertained that instances of dynamism (MDyn’s versions of individual causings) can in their own right be the source or target of dynamism. Moreover, meta-dynamism instances, as a special case of dynamism instances in general, can interact dynamically with other aspects of the world, and such interaction is then meta-meta-dynamism. There is no in-principle limit to the number of “metas” one can add here.

### 1.4. The Bridge between Sides: The Physical Reification Postulate

MDyn is not only radical as an anti-Humean approach in proposing meta-dynamism as well as dynamism, but it also claims that, if we posit that dynamism exists at all, it is entirely artificial not to posit it as a “first-class citizen” of the physical world. That is, *it is artificial not to posit dynamism as a fully physical aspect of the world, no less genuinely physical than items mentioned in existing versions of physics, such as the familiar particles, fields, spacetime curvatures, etc. might be postulated to be* (depending on one’s particular outlook on physics). This promotion of dynamism to first-class citizenship is what I will call the physical reification of dynamism, as opposed to some more mysterious merely metaphysical reification of dynamism as something that supports but does not appear in the physical world. Dynamism must therefore be accounted for by mathematical physics, with some suitable mathematically couched physical laws that are explicitly about dynamism.

Once the reification step is taken, meta-dynamism is, as a special case, automatically a physical aspect of the world. Meta-dynamism is the physical, dynamic interaction of dynamism instances with ordinary aspects of the world or indeed with dynamism instances and even possibly with themselves.

To sum up, dynamism is not just the causal interaction of ordinary physical aspects with each other but also includes the interacting, with aspects of the world, of that interacting. Moreover, because consciousness is a matter of auto-meta-causation, it is a matter of *auto-meta-dynamism*—a matter of episodes of experience being *physically* sensitive to their own internal dynamism, via meta-dynamism. *This is the physicalist hub of MDyn.* MDyn involves a “physics plus” but is still physicalist.

### 1.5. The Crux of the Physical-Theory Side

MDyn takes a standard view of the physical world as being described by mathematical equations that couch physical laws that specify how the values of physical quantities constrain each other over time. This mutual mathematical constraining reflects the interaction that constitutes dynamism. Thus, we can view the laws as in some sense capturing dynamism. But of course, the equations do not mention dynamism itself—they just mention familiar physical quantities such as masses, velocities, field strengths, curvatures, etc. and rates of change of such with respect to space and time. In sum, the law equations in existing physics *capture* dynamism by *mentioning* ordinary physical quantities, but do not *mention* dynamism itself. These laws are *base-level dynamic laws*.

Given this picture, plus now the Reification Postulate, dynamism can be regarded as physically reified law-*governedness*. However, it does not reify physical laws as such. Laws remain as human-crafted descriptions that reflect the objective dynamism.

Introducing meta-dynamism means that there are interactions between dynamism instances themselves and other aspects of the world, be they ordinary or themselves dynamism instances. MDyn assumes, naturally, that these interactions are captured by laws. Thus, MDyn proposes that there are laws that capture meta-dynamism by mentioning dynamism instances themselves (along with ordinary physical quantities as well, typically). Such laws are *meta-dynamic laws*. Things can become tangled at this point, because the dynamism instances mentioned in a law can themselves consist wholly or in part of meta-dynamism, in which case the law is to that extent meta-meta-dynamic.

The physical-theory side of MDyn is a general prescription for what meta-dynamic law equations look like, and how in general they operate. This side also, of course, provides a framework for particular equations, derived from the general laws, describing a particular physical system that includes meta-dynamism. The development of MDyn has not nearly reached the stage where it can propose specific meta-dynamic laws, as opposed to a general framework for such laws. However, it has got to the stage that we are able to set out equations for toy physical systems (see Section 3.11, Section 3.15 and Section 3.16) that include meta-dynamism, where those equations would be derived from application of putative, yet-to-be-specified physical laws to the particular system at hand.

MDyn currently proposes, mainly, that meta-dynamic laws are meta-dynamic modifications of existing, base-level dynamic laws. The modifications are a matter of adding additional factors, terms, etc. to the law equations. However, entirely new laws are not in principle precluded. MDyn makes some specific proposals about the nature of dynamism instances and of physical quantities based on them that can be mentioned in meta-dynamic laws and system equations.

In particular, MDyn considers a dynamism instance to be defined as occupying a region of spacetime, which can in particular be a single spacetime point but can alternatively extend over regions covering large swathes of space and time. MDyn proposes that laws can contain *region-valued expressions* involving mathematical functions that deliver regions of spacetime occupying spacetime intervals before or up to the present time. The regions are selected not by specifying their spacetime coordinates but specifying their physical contents in a limited range of possible ways. MDyn provisionally proposes that a dynamism instance has various different *intensities*, such as the intensity of the base-level dynamism within it and the intensity of meta-dynamism within it. Such intensities can be evaluated by functions usable in laws.

The proposed provisions for mention of dynamism instances in meta-dynamic laws make such laws inherently *time-asymmetric*. This departs from the celebrated (if possibly imperfect [27]) time-reversal symmetry of laws in current physics. I leave this matter for further discussion elsewhere. The provisions also make MDyn’s “physics plus” be *temporally non-local* [28], a matter that Section 5.7 takes up again, briefly. For now, the intuitive effect is that the current state of a system is not just the result of continuous evolution of state up to the current time, but also involves *time-hopping* in that current state is *directly* constrained by state that can be arbitrarily far in the past. Furthermore, there can in effect be chains of time hops. A state can depend in part on past state that itself depends, in part, *directly* on state that is past relative to it. (Here state includes not just ordinary, familiar physical quantities but also dynamism-instance based quantities.)

The introduction of meta-dynamism into physics results in the need for a highly reflexive conception of how state evolves. The dynamism accessed by the operation of a law at a particular place and time can include aspects of the dynamism at that place and time, and these can include the dynamism inherent in the operation of this very law. As a result, Section 4.2 proposes that we should conceive of the physical quantities over space and time as forming a web of constraint *where the whole web is itself, reflexively, a node within itself.* This “self-swallowing” nature of the web may be perhaps the key addition to physics that MDyn makes in the service of consciousness.

### 1.6. Normal Invisibility of Dynamism

Of course, current physical laws provide exquisitely accurate predictions about many aspects of the world, so the new laws or law features proposed in MDyn should only have significant effects inside certain types of system, notably conscious ones. Outside such systems, the divergences of behaviour, if any, that they introduce must be small enough to have been undetectable so far. In that sense, physically reified dynamism is, normally, effectively “invisible”: it is physically there, but it does not in practice matter (much). Its meta-dynamic effects are tiny. This is provided for by MDyn because of the way that dynamism instances are accessed in laws. Expressions accompanying or incorporating mentions of these instances can control how significant an effect they have on other quantities in the law, and ensure the effect is only significant under specified physical circumstances.

Of course, a central aspect of MDyn is that, according to it, consciousness is *not* physically invisible. It has significant causal power in the world, as pointed out in Section 1.7.

The invisibility of the new laws or features outside, e.g., conscious systems is part of the “conditionality response” that Cucu and Pitts [29] discuss. This response is not in fact about physicalist theories such as MDyn, but is instead a rebuttal of objections to dualist views in which consciousness inhabits a realm that is beyond the physical but nevertheless dynamically interacts with it. In essence, however, Cucu and Pitts’s arguments are telling for physicalist theories as well as dualist ones. They support the legitimacy of proposing disturbances to normal physics inside conscious systems, irrespective of whether the disturbances involve just a modified physics (as in MDyn) or interactions with a non-physical realm. Current empirical results about the physical behaviour of occurrently conscious human brains is not yet at the stage of testing whether current physical theory is as accurate inside them as outside.

### 1.7. MDyn as a Physicalist, Broad Identity Theory

As a result of putting together its particular philosophical and physical-theory sides, MDyn in its strongest form is an identity theory of phenomenality. It identifies *being-occurrently phenomenal* with *having appropriately arranged, physical auto-meta-dynamism*. It is not that phenomenality “emerges” from such auto-meta-dynamism in any strong sense—rather, it *is* such auto-meta-dynamism.

While I cannot argue the point here, the intuitive strangeness of consciousness in contrast to what we commonsensically regard as the physical world—and that raises the “explanatory gap” between the physics and consciousness and the “hard problem” of consciousness—is explained as the intuitively strange, yet formalizable, nature of meta-dynamism, especially when turned back on itself in auto-meta-dynamism.

The qualification “in its strongest form” is included just above because, below, we will separate the brunt(s) of MDyn into a *Necessity Claim* and a *Sufficiency Conjecture.* The Necessity Claim is to the effect that a conscious process must be constituted in part by an appropriate arrangement of auto-meta-dynamism. The Sufficiency Conjecture is that such an arrangement actually constitutes phenomenality in at least a core form. It is this conjecture that ties up MDyn as an identity theory.

Because MDyn is a physicalist identity theory, it makes consciousness non-epiphenomenal: rather, consciousness has causal power upon the physical world (and is causally affected by it). The arrangement of meta-dynamism constituting the phenomenality in a conscious process is physically caused by prior circumstances (in fact, this causation is itself meta-causation), and can directly, meta-causally affect the world spatially outside or temporally after the process.

Identity theories are often talked of as “mind/body” or “mind/brain” identity theories. However, MDyn is an identity theory in a much broader, less committal sense, as follows.

### 1.8. Multiple Realizability, Bathypsychism, Non-Panpsychism, and Scale

MDyn makes consciousness multiply realizable in that the claimed meta-dynamism is not required to reside in any particular type of matter or system, such as biological matter or systems. The use of “realizable” here should not be taken to imply a difference between mental states and the physical states they are identical to. I am just taking identity as an extreme form of realization, albeit that the identity is to a very broad type of physical circumstance, defined in terms of meta-dynamism. By realizable in a particular type of physical matter, etc., I here simply mean residing physically in it.

As [19] says, there are arguments on either side of the question of whether or not consciousness must reside in, or is most likely to reside in, in biological systems. See [14] for the “most likely to reside in” sort of claim, and for general discussion of biological naturalism and realism about consciousness. MDyn does not even rely on residing in any particular physical aspect of the universe, such as its electromagnetic aspect, its gravitational/curvature aspect, etc. Potentially, the right interactions might exist in arrangements of many different specific types of physical matter, field, etc. Furthermore, the theory does not rely on consciousness being within an object in the everyday sense, as opposed to, say, within diffuse, disembodied fields. I am not making any particular claim here about where consciousness may actually reside, but merely pointing out the liberality of the theory.

However, it may in practice turn out, for reasons of stability and survival, that consciousness can only successfully reside in tangible objects; it could then be that these objects will tend strongly to be composed of a particular form of matter in some range of temperature, pressure, etc., and, further, that in an environment like our planet these are only likely to arise naturally within biological systems. This article does not address these pragmatic matters.

MDyn is *bathypsychic* in characterizing the conditions needed for consciousness at a *deep* physical level, rather than postulating conditions that intrinsically involve high-level entities or structures. (The author introduced the term “bathypsychic” in [1], given that “bathy” refers to depth, as in the submersible vehicles called bathyspheres.) However, bathypsychism, even when combined with the multiple realizability (and with the possible simplicity of consciousness to be discussed in Section 5.6) does not imply panpsychism. Panpsychism (see, e.g., [30]) is some form of the idea that consciousness in at least some elementary form is fundamental and ubiquitous—it exists throughout the micro-fabric of the universe, even possibly within individual elementary particles or other small ubiquitous physical systems—and even to the extent perhaps of being the foundation of the physical world itself. While panpsychic theories tend strongly to be bathypsychic, or can be constitutively bathypsychic, a bathypsychic theory need not be panpsychic. Section 4 and Section 5 reveal opportunities for MDyn to confine consciousness to certain regions or structures. This possibility exists partly because MDyn (probably) requires some special form of meta-dynamic auto-sensitivity, not just any such. The special form might only arise under special physical circumstances, even if meta-dynamism in general were to turn out to be ubiquitous.

Relatedly, while MDyn is bathypsychic, this does not necessarily mean that it is (only) about a microscopic scale. Just as, say, quantum theory is not confined to the microscopic scale, some meta-dynamism might intrinsically exist at a large scale.

MDyn does not hold that conscious processes are the only locus of meta-dynamism. MDyn holds that conscious processes involve some particular form of meta-dynamism (yet to be fully elucidated), and there is plenty of room for other forms of it to exist, in principle. There may well be important and perhaps widespread forms of meta-dynamism that have nothing at all to do with consciousness. An intermediate possibility is that there are forms of meta-dynamism that are precursors to consciousness in evolution or in foetal development. In addition, meta-dynamism could conceivably be a new weapon in seeking to explain puzzling phenomena other than consciousness. This matter must be left for discussion elsewhere.

### 1.9. The Starting Assumption on the Philosophical Side: PRAIS

As well as assuming a form of anti-Humeanism, the philosophical side of MDyn takes as a starting assumption something inspired by a much discussed and adopted, if contentious, claim—namely that all consciousness constitutively involves *pre-reflective self-consciousness* (PRSC), otherwise known as pre-reflective self-awareness. This claim has been recently much discussed by neo-phenomenologists and commentators (see e.g., [21,22,31,32,33,34,35,36]), carrying forward a theme in the work of phenomenologists such as Merleau-Ponty and Sartre, and also Husserl in at least one interpretation [21]. (See the cited modern works for historical references.) The claim about PRSC is that in [phenomenally conscious] experiencing, that experiencing is automatically also, in some way that may be difficult to articulate, continuously experiencing that experiencing. This experiencing-of-experiencing in the form of PRSC does not involve what we normally regard as conscious introspection—introspection that includes “reflective” mental actions such as concept applications, reasoning, and so forth that we normally take to constitute thinking. Instead, we somehow have conscious awareness of our consciousness in some more direct, basic way. As this article does not in fact assume PRSC, there is no need to theorize at this point what way this might be, but it could be, for example, a not-necessarily conscious variant of direct acquaintance [37,38,39]. Another candidate, in line with claims of “cognitive phenomenology” [40], is that it is a *feeling of*, as opposed to a thought about, our being-conscious.

A terminological point: Although the issue of “reflective” happens to be raised here in the context of possible thoughts about one’s own mental states, where we turn back to or into our own mentation, the term “reflective” as used in “pre-reflective” is more general. It does not involve the metaphor of something turning back on itself. Rather, it is about ordinary thinking as when we “reflect on” some issue that has nothing specifically to do with ourselves, such as the state of the oceans. So “pre-reflective”, which could also be rendered for most purposes in this article as “non-reflective”, does just mean non-conceptual, non-propositional, non-inferential—non-intellectual generally. It alludes to a mode of internal processing that could conceivably occur within an animal radically lacking our cognitive make-up. I will reserve the term “*reflexive*”, in a mental context, to cases of the turning-back-on-itself metaphor. Reflectiveness and reflexiveness are orthogonal. Thus, PRSC is a form of reflexive but non-reflective consciousness, and thinking about the ocean is reflective but non-reflexive.

Proponents of PRSC hold that the [phenomenally] conscious aspect of conscious, reflective introspection is provided by PRSC, not by the sheer fact that one is reflecting on one’s mental states or processing. Thus, the claim repudiates, in particular, HOT theories (Higher-Order Thought theories) of consciousness [41,42]. In such theories, one has, for instance, a conscious belief that X by virtue of having a belief about that belief (with suitably tight restrictions on the internal support for the meta-belief). Indeed, it is consistent with proposing PRSC to propose that one can alternatively have entirely *non*-conscious reflective mental states, inferencing, etc., about one’s own mental states or processing (whether they are themselves conscious or unconscious).

MDyn takes on board the pre-reflective reflexivity of PRSC, but weakens it in an important respect. MDyn’s actual starting assumption is not that consciousness always involves PRSC, but instead is one that concerns *pre-reflective auto-individuating auto-sensitivity* (PRAIS), operating physically. MDyn assumes that any conscious episode is a physical process and that, at every moment in its time-span, is *physically sensitive*, in a *pre-reflective* way, to its own existence (so far) as a physical, conscious process, that is a *unit* that is furthermore *differentiated* (individuated) from the world outside itself. The key difference of PRAIS from PRSC is that the auto-sensitivity is not presumed from the beginning to be conscious. Thus, we have a starting assumption that does not circularly involve consciousness. Further motivation will be given in Section 2.1.

Because PRAIS does not itself refer to consciousness at all, it does not in itself commit to any claim that consciousness always involves s form of self-consciousness. So, in principle, it could turn out that there is pre-reflective consciousness of external objects that does not involve any PRSC. However, Section 5.3 will argue that in fact, because of PRAIS, consciousness does always involve a restricted form of PRSC.

There is also a sense in which PRAIS strengthens PRSC. PRSC has no physicalist commitment, whereas PRAIS does. One could be a dualist and support PRSC. Equally, one could imagine a non-physicalism-assuming form of the PRAIS assumption, in which case the actual form used in this article could be called a physicalist version of it.

PRAIS also explicitly mentions the auto-individuation quality. PRSC does not do so, but I contend (though cannot fully argue here) that PRSC has always implicitly presumed auto-individuation. Under PRSC, the experience (or its subject or “I”, if distinct from the experience) is conscious of *this experience*. It is not simply conscious of *some* experience, or conscious of being immersed in experiencing in general, with no border between itself and the latter. To use a metaphor, according to PRSC, the experience is a particular current in the ocean of activity of the world where the current is not just aware of the movement of the ocean it is in, and therefore only implicitly aware of itself in some weak sense, but, instead, aware of itself being *this* current. (See also the discussion of “ipsiety” in [21].)

While PRAIS as such is a particular combination of properties that may not have been explicitly considered before, the centrality for consciousness of some form of auto-sensing by a process is not novel. Illustrations, amongst many possible, include [14,43,44] (see also [45]). Furthermore, if auto-sensitivity is a form of direct acquaintance, then it implies acquaintance with acquaintance. Such meta-acquaintance is mentioned in, for example, [37,46] and cited as a prominent idea by [47].

Because of the pre-reflectiveness, the PRAIS assumption readily allows for the in-principle possibility of consciousness in tiny infants, foetuses, and non-human organisms, going perhaps quite a way down in the biological hierarchy.

### 1.10. From the PRAIS Assumption to Meta-Dynamism

A key argument on MDyn’s philosophical side starts from the PRAIS assumption to the Necessity Claim mentioned in Section 1.7 and to be set out in Section 2.6, *viz* the claim that a conscious process must be constituted in part by an appropriate arrangement of auto-meta-dynamism. Important parts of the argument are relegated to Appendix A.2,Appendix A.3,Appendix A.4,Appendix A.5,Appendix A.6,Appendix A.7. I just give some highlights here.

The argument rests first of all on a prior argument (see Appendix A.3) that one cannot define consciousness just in terms of *trajectories of ordinary state* (e.g., electromagnetic or positional states) that a process goes through. Rather, one needs the states to be linked with each other by dynamism. The intuitive crux of the arguments on this point is that, without bringing in that dynamic binding, a suitable series of completely unconnected state sub-trajectories could, highly implausibly, have to be accepted as a unified, uninterrupted conscious process: e.g., a succession of sub-trajectories, each of which is in a different person’s brain, with essentially no interaction between between the brains.

Now, turning to the assumption that a conscious process involves PRAIS at every moment throughout its existence, one might think that the auto-sensitivity at a given moment could readily be achieved just by having the state at that moment hold some sort of representor of some or all of the prior states and possibly also their dynamic binding. It would remember a recent history of states and possibly their dynamic binding. So, part of the causation of a state as the process unfolds would include the causation of the updating of that representor. (I use “representor” for an item that represents, allowing me to reserve “representation” for talking about representing or the topic in general.) The representor could be of one of the many forms proposed in theories of representation in cognitive science, AI, etc. (see, e.g., [48] for comprehensive survey and discussion of the fundamental space of possibilities).

However, I argue (see Appendix A.2) that there is no theory of representation that is *completely* objective—free of *any* subjective (i.e., observer-relative) construal of what is represented by what—and *completely* pre-reflective. The objectivity point is supported by, for instance, [49]. Furthermore, [50] advocates a somewhat prominent view that the representational properties of mental states themselves depend on phenomenal experience, and presents well-known problems suggesting the probable inability of current non-phenomenally based theories of representation to provide an objective account of representation. Of course, a phenomenally based form of representation is no use for the purposes of this article, as it would create a circular argument.

So I conclude that representation is probably incapable of being the basis of an entirely objective attribution of PRAIS. Since MDyn does hold that consciousness is a purely objective feature of physical systems, representation is an inadequate construct for our purposes.

But if extant theories of representation cannot work for the purpose at hand, what can? MDyn’s answer is that, at any moment the very dynamism within the process and (partly) leading to the current state is also itself something that directly affects current state. So the affecting of current state is meta-dynamic in that it explicitly involves dynamism as an entity in its own right.

This answer provides an entirely objective, pre-reflective sense in which the current state is sensitive to its own history. Simultaneously, it answers a question implicitly left dangling above: the question of *why* causal binding matters in characterizing consciousness. On the question of whether a particular sequence of states constitutes a conscious process or not, why should it matter whether or not the states arise from each other naturally through normal causal binding amongst them, as opposed, say, to their being forced on a system by outside influences? In effect, MDyn’s answer is that *the binding within a conscious process matters because it matters to that process itself.* (Note that we are not talking here about whether *consciousness* matters in the physical world. MDyn takes that as given, and see Section 1.7 on the causal power of consciousness on other aspects of the world. We are just discussing what matters about the physical world for the purposes of consciousness.)

The arguments summarized here are not ones of secure logical deduction, and to an extent couch meta-dynamism as the best explanation for PRAIS, rather than proving that it is the only possible explanation. But I claim that the arguments are at least highly suggestive.

The argument only establishes that conscious processes are partly constituted of some form of auto-meta-dynamism, where it may be that necessary further details of that form remain to be uncovered, and it may be that there are further necessary conditions for consciousness, of different sort. The argument does not verify a Sufficiency Conjecture to be set out in Section 2.7, *viz* that if a process contains that form of auto-meta-dynamism then it is guaranteed to be pre-reflectively conscious.

### 1.11. Structure of the Article

Section 2 says more about the main assumptions, tenets and stances on the philosophical side of MDyn. Section 3 and Section 4 present the current progress in giving MDyn some precise mathematical clothing, largely concerned with the question of how to include dynamism and especially meta-dynamism in physical laws. The approach cannot, at present, provide a complete specification of some conscious system or a class of conscious systems, but it does provide equations for simple toy systems that illustrate the operation of meta-dynamism, including the type of meta-dynamism that MDyn claims is necessary for consciousness. The current treatment is limited and simplified in assuming a classical physical framework aside from the introduction of dynamism, leaving the task of embedding the MDyn philosophy in an extended quantum-theoretical or relativistic framework to future work.

Section 5 engages in discussion and further remarks. In particular, the section argues briefly that MDyn thereby holds the promise of being able to provide a model of phenomenality that is at its core very simple, involving a meta-dynamic auto-sensitivity that is describable in a relatively simple way in mathematical system equations. The discussion exploits a notion of core consciousness introduced in Section 2 and that consists largely of some core feelings (notably, a feeling of own continuing existence, and, possibly, basic forms of pleasure and pain).

Section 6 briefly concludes the main text, emphasizing certain issues and raising some topics for future research not already mentioned in passing.

There is also an Appendix, setting out more of the philosophical thinking and arguments informing MDyn.

## 2. Further Details of the Philosophical Side

Here I comment on PRAIS *versus* PRSC, expand on the process basis of MDyn, explain PRAIS and (meta-)dynamism further, and present a Sufficiency Conjecture and some Adjuncts to it, to the effect that some suitable form of meta-dynamism is not just necessary but also sufficient for—indeed, constitutive of—consciousness in at least some core form.

### 2.1. Replacing PRSC by PRAIS: Reflexivity *versus* Transparency

Adopting PRAIS rather than PRSC as a starting assumption is partly to avoid having a consciousness-based condition for consciousness. But there is also a more tactical reason, namely that the PRSC claim has been controversial. Many researchers claim that PRSC is outside their phenomenal experience, e.g., they deny that when they are conscious of a red rose they are also conscious of being conscious of it. Thus, they support the “transparency” of being conscious of something [51]. Of course, a dispute on such a matter is immediately complicated by the fact that one is philosophically reflecting conceptually on an experience that one is not currently having. Let us agree that in being conscious of a rose one is typically not *reflectively* conscious of the consciousness—one is not thinking conceptually about it in any ordinary sense. But in theorizing philosophically about the conscious episode, it is very difficult to consciously imagine or remember what it is like to engage in that episode without reflecting on it (thinking about it) but instead having pre-reflective consciousness of it. This is problem affecting the possible judgments of people on both sides of the dispute.

I follow [35,52] and others in taking that transparency objection to pre-reflective auto-consciousness to be flawed, and not fully appreciating the subtlety of phenomenal states. My own claim is that the consciousness of the rose is what one might call a “my-rose-ish” experiencing, where its being an *experience* at all lies precisely in the fact that it includes pre-reflectively experiencing the “own-ness” of one’s experiencing. I claim that in being conscious of a rose, while not reflectively introspecting on that consciousness, one is consciously experiencing (at least) its rosishness-for-oneself, not just experiencing particular rosishness. By a particular rosishness I mean an instance of rosishness at a particular time, at a particular place in the visual field and from a particular visual perspective, a first-person perspective. Thus, the “for-oneself” rider is over and above those particularities. But it is not an extra sort of consciousness beyond the consciousness of the rose. Rather, it is part and parcel of being conscious of the rose. One’s experience simultaneously and unifiedly has a rosish experiential quality and a reflexive experiential quality. Instead of putting the matter as “rosishness-for-oneself” one can (I claim) put it better as “rosishness-in-the-current-experience-belonging-to-oneself” or (yet better) “rosishness-in-one’s-own-experience” or “rosishness-in-this-current-experience”. In experiencing *this* quality, one is therefore tautologically engaging in a reflexive relation to one’s own experience. A point here is that there is a linguistic trap of thinking that “conscious of the rose” is the same as “experiencing the rose” where actually it is the same as “experiencing the rosishness-in-one’s-own-experience”. (These brief comments owe much to other authors’ works such as [35,52] and researchers who focus on the “mineness” or “for-me-ness” of experience—e.g., [22,33] amongst any others—though I differ from, notably, [22] in repudiating the idea that the conscious state *represents* itself. However, putting the representation issue aside, my claim is tantamount to saying that the “for” in “for-me” or “for-oneself” means “in-the-experience-belonging-to”. I believe that this is what past researchers have meant by it.)

Thus, in sum, from concerns about transparency, I take a caution about assuming a too-rich form of auto-consciousness while still averring that there is always a restricted form of it in consciousness.

However, for yet greater methodological safety, and also to avoid having experience (consciousness) within the statement of the PRAIS condition, PRAIS has been made yet weaker in a sense, by confining its explicit reflexiveness to the auto-sensitivity, where this is not claimed to be a conscious sort of sensitivity. Nevertheless, we will argue in discussion Section 5.3 that, because of PRAIS, a conscious process does in fact always have a variant of PRSC, namely pre-reflective consciousness of its own PRAIS. With the addition of the Sufficiently Conjecture (Section 2.7) as augmented with one of the Adjuncts in Section 2.8, this form becomes a restricted form of *pre-reflective-consciousness of its own pre-reflective consciousness*, or *auto-pre-reflective consciousness* for brevity. By contrast, in PRSC as usually discussed, there is pre-reflective consciousness about own consciousness, with no restriction of the *latter* to pre-reflective aspects of consciousness. Auto-pre-reflective consciousness may be especially difficult to notice in oneself or to remember, and may therefore be relatively immune to transparency objections.

### 2.2. Consciousness and Physical Processes

This article has slipped in, so far without comment, a view of consciousness (phenomenality) as being, in the first instance, a potential physical property of physical *processes* (behaviours). Consciousness is a matter of episodes of experienc*ing*. This is opposed to thinking of consciousness as being a property of things such as mental states, neural networks, creatures, computers, computer programs, etc. Such things are only conscious in a derivative sense of involving a conscious process. Thus, MDyn finds “process philosophy” (e.g., [53]) congenial, though it does not assume any particular existing form of process philosophy.

Moreover, I assume that consciousness is an *objective* property of processes. A given process is or is not conscious, or is conscious to some degree, as a matter of objective fact, ultimately definable in purely physical terms, and not requiring any observer to construe the process as being conscious.

But I do not assume that there is any objective demarcation of the physical world into processes. Rather, given the (objectively or non-objectively based) identification of the evolution of particular parts/aspects of the physical world as a process, it is an objective, physical matter how conscious it is. However, in Section 2.5 we will see that the auto-individuation aspect of PRAIS goes some way to providing a naturalistic demarcation of *conscious* processes.

I make the following further assumptions about processes in general and about conscious ones.

A process lies in a specific spacetime region (not necessarily a connected one) and involves at each moment certain aspects of physical state within that region (e.g., electromagnetic aspects, positions of certain particles, or what have you). The world “*outside*” the process covers both (a) the physical world outside the region occupied and (b) aspects of the world that are within the region but that are not included in the process. The process includes any dynamism that underlies the changes of state within the process, plus any “incoming” and “outgoing” dynamism, i.e., dynamism inherent in effects on the process from outside or vice versa.

Some selected aspects of the incoming or outgoing dynamism of a process may be regarded for some purpose as being, respectively, the “input” or “output” dynamism of the process, such as via sense and motor organs of a biological creature. However, this is a matter of construal by human observers, not an objective matter. I consider it in principle possible for a conscious process to have no significant interactions with anything at all outside itself—to be entirely “inward-looking” and “inward-acting” for all practical purposes—even if this is unlikely in practice to occur in consciousness arising naturally in creatures embedded in an environment.

Aside from observer-construed input and output, the process may have all sorts of incidental incoming and outgoing dynamism that would (at least normally) be considered irrelevant to consciousness, and may be vanishingly small, such as gravitational effects of the red rose one sees on neurons in one’s brain, to take a particularly extreme example to make the point.

A process may, intuitively, move as a unit through space. For instance, a process in a car engine or brain moves around in space as the car or person moves around. So in general the spacetime region occupied by a whole process over time will have a very complicated, wiggly shape through spacetime, if one thinks of the latter as a 4D block. Such movements cannot affect whether the process is conscious or not, given a fixed pattern of internal activity and of interaction with the outside.

The concept of a process in MDyn allows both for cases where a process is viewed as being on some substrate such as a brain or computer, with the process viewed as different from the substrate, and cases where no division is considered: there is just activity, be it movements of matter or some other sort of activity. In the latter, substrate-free, view, the activity may include that of the substrate under the former view. When the substrate view is taken, then I assume that the particular identities of components of the substrate are unimportant: replacement of functionally identical components does not affect the nature of the process, and in particular does not affect whether it is conscious or not. This assumption connects to common thought experiments such as those concerning replacing the neurons of a conscious brain by other neurons or by artificial, identically operating components (see, e.g., [54,55]). In MDyn, though, the functional equivalence has to extend to the dynamism involved, especially the meta-dynamism.

### 2.3. Punctate and Holistic Regional Dynamism

Dynamism is defined both at points (spacetime locations) and over regions. The dynamism at a spacetime location (place and time) *l* is the systematic pushing-forward of the universe as restricted to how it is constraining aspects of state at *l* with respect to each other (hence, synchronically) and with respect to aspects of state elsewhere in spacetime (hence, partly diachronically). The dynamism over a region is the systematic pushing-forward as restricted to how it is constraining aspects of state anywhere within the region with respect to each other or world aspects outside, synchronically and diachronically.

The dynamism over a region—a *region of dynamism* or *dynamism region*—can be considered to be composed from the punctate dynamisms at each of the locations within it. However, just as a set is more than the plurality of its members, the dynamism over a region is more than the plurality of its component punctate dynamisms. (Indeed, for the purposes of this paper it may be adequate to take it to be just the set of those punctate dynamisms, or a function from the locations to their punctate dynamisms.) The having-been-collected-together feature of the region is important, just as it is for a set of numbers, say.

We will therefore often emphasize the holistic or unitary nature of a region of dynamism. Its being a unit in its own right is an important tool in conceiving of a conscious process as being, at a given time, sensitive to (part of) its own history *taken as a unit*.

Nevertheless, I will assume that the plurality of punctate dynamisms in a region logically determines the dynamism region, and vice versa. This is again analogous to a set of numbers.

Physically, the unitary nature means that a region of dynamism can physically interact with other aspects of the world in a way that is not a matter of the combined interactions, if any, of the punctate dynamisms with that aspect.

A complication in the notion of punctate dynamism is that, when state at the location is affected by a region of dynamism, that affecting is part of the punctate dynamism at the location. So the punctate-ness is confined to the dynamism being indexed by a particular location and being focussed on the constraining of state at that location, rather than being about what parts of spacetime are involved overall.

We will often talk of dynamism as defining, embodying or otherwise providing mathematical constraints between quantity values, whether at the same location or at different locations. This is discussed in some detail in Section 4.2 in particular.

### 2.4. More on Pre-Reflective Auto-Individuating Auto-Sensitivity (PRAIS)

It could be said that there is a vacuous sense of auto-sensitivity that applies throughout any process. The state at any moment of any process is of course moulded by the normal evolution of the world in the process’s spatio-temporal region up to that point. Thus, PRAIS must be about some special form of auto-sensitivity that goes beyond this generic, basic form. For the sake of illustration, one possibility to consider (as mentioned in 1.10) is that each momentary state during the process holds a representation of some portion of the sequence of states within the process up to the present moment. The PRAIS assumption demands that the history of the process (from some previous time within the process) up to this moment does not just lead in the normal way to that state, but rather, its being-the-history itself has an effect on the current state. To put it metaphorically, the process at each moment “notices” its own history (at least for some time into the past) as an entity in its own right and as being its history. But this language is misleading, as it could by misunderstood as implying conscious, reflective noticing.

As part of being “pre-reflective” (non-conceptual, non-propositional, non-inferential), the auto-sensitivity in PRAIS does not amount to a proposal that a conscious process conceives of itself as a physical, conscious process, etc.; indeed, the process may be within a system that is incapable of conceiving anything at all. On the other hand, a conscious process in a human being *may* conceive of itself as a physical and/or conscious process, as well as being pre-reflectively sensitive to itself.

The PRAIS assumption requires that a conscious process possess PRAIS *throughout*, i.e., the mentioned auto-sensitivity exists at each moment in the process’s time-span. This is demanded because any break would lead to the process being more properly viewed as only intermittently conscious. So the question of consciousness would be pushed down to break-free portions. Thus, the remainder of this article is implicitly about *uninterrupted* conscious processes.

Moreover, as already hinted in passing, MDyn does not assume that a conscious process is at each moment sensitive in any direct way to *all* of its existence so far as a conscious process. The PRAIS assumption only demands that the current state be sensitive to its dependence on a recent segment (reaching up to the current time) of its history. One could view this as a matter of a sliding window of auto-sensitivity, perhaps akin to notions such as the “specious present” (see, e.g., [56], and [35]: Chs. 4–6). This sliding window indirectly implies the auto-individuated existence of the whole process so far, even though the process at the present moment is directly sensitive only to a part of the whole history. This exactly fits an observation by [57]: it may be that a subject’s having a phenomenally unified consciousness over an extended period of time is simply the subject’s continuously having a consciousness that is phenomenally unified within the specious present as this slides along through the period.

### 2.5. Auto-Individuation, Processes and Objectivity

The “*auto-individuating*” aspect of PRAIS, i.e., the sensitivity of the process to itself as differentiated from the world outside itself, is as follows. It consists in the process being sensitive to its own prior activity in some way W in which it is not sensitive to activity outside itself, where W is uniform across processes (it is not dependent on which process we are considering). What that way could be is partly addressed by the remainder of this article.

In considering what conscious process exists in, say, a brain, it may be that there is no prior, objective fact of the matter about how much of the brain’s activity should be included (recall comments above about what counts as a process). However, it is plausible that the requirement of auto-individuation in PRAIS significantly restricts the spacetime regions that can turn out to be home to a conscious process, since the process must be sensitive to its inner nature in a distinguished way (W above) in which it is not sensitive to the world outside it.

However, as that way is not yet fully elucidated, there is a wide range of possibilities, especially if the sensitivity difference is a matter of degree. Thus, a process being self-individuating may not of itself prevent some process spatially within it from also being self-individuating. I must leave this possibility for future investigation.

### 2.6. The Meta-Dynamism Necessity Claim

To sum up so far, the argument about achieving PRAIS outlined in Section 1.10 leads to the *Necessity Claim* of MDyn:

There is a way W of being meta-dynamically sensitive to dynamism such that *any conscious process must, at each moment in its time-span (however short or long), be meta-dynamically sensitive in way W to dynamism taken as a unit over some time interval leading up to the current moment, and must not be meta-dynamically sensitive in way W to dynamism outside the process.*

The following clarifies some aspects of the claim.

- There may be further conditions that a process’s meta-dynamic auto-sensitivity might need to satisfy in order for the process to be conscious. Thus, all the Claim says is that *some “suitable” form of* meta-dynamic auto-individuating auto-sensitivity of type W is required, i.e., an extra suitability condition may need to be satisfied. Whether such a condition is needed and what it might be is also yet to be fully determined (though one suggestion, about “centred reflexivity”, is made in Section 3.14). It may, perhaps, involve a further restriction on the qualitative type of sensitivity, and/or on some particular arrangement of qualitatively different auto-sensitivities, and/or a threshold of sensitivity intensity (if W does not already involve a threshold).
- However, on the other hand, it could in principle be that W turns out to be vacuous, i.e., just *any* way of being meta-dynamically sensitive to dynamism. Thus, the Necessity Claim would require that the process be meta-dynamically sensitive to a sub-history of itself in some way or other but not to be meta-dynamically sensitive in any way at all to dynamism in the world outside.
- The meta-dynamic auto-sensitivity is itself part of the process’s dynamism, and as such can in turn be part of the dynamism that the process is sensitive to. As a result, the theory leads naturally to the idea that the meta-dynamic auto-sensitivity is, at least sometimes, and at least in part, constituted of *auto-sensitive meta-dynamism*. This is meta-dynamism that is (meta-meta-dynamically) sensitive to itself. It is a special case of “auto-meta-dynamism”, which alludes to a process that is meta-dynamically sensitive to some of its own dynamism, with no assumption that the latter includes meta-dynamism.
- Moreover, the auto-sensing/affecting in the auto-sensitive meta-dynamism is itself part of that very meta-dynamism, so the reflexivity in the “auto” can be said to be *internal*. (This contrasts with the possibility that the auto-sensitivity goes via a channel of activity external to the meta-dynamism that is being sensed.)

### 2.7. The Sufficiency Conjecture

The Sufficiency Conjecture is that

*having, throughout, some suitable form of auto-individuating meta-dynamic auto-sensitivity is enough to make a physical process conscious in at least some pre-reflective, basic phenomenal sense. Furthermore, this is not about mere logical sufficiency. Rather, the meta-dynamic auto-sensitivity IS the process’s conscious phenomenality.*

The Sufficiency tenet is labelled as a Conjecture rather than a Claim because MDyn does not have substantive arguments for it from premises, unlike the case of the Necessity Claim. Rather, it is presumed as a possibly fruitful line of further investigation.

### 2.8. Adjuncts to the Sufficiency Conjecture

On the supposition that the Sufficiency Conjecture is true, there are riders or adjuncts that one can add, giving more specific forms of the theory. They are not just arbitrary, but themselves motivated by the thinking leading to the Necessity Claim. Six such are as follows, building on each other.

*Adjunct 1A: Existence of Core Meta-Dynamism:* There is a unique, core form of the suitable meta-dynamic, auto-individuating auto-sensitivity as mentioned in the Sufficiency Conjecture. That is, all consciousness, no matter how devoid of other physical structure or relationships to its environment, involves this *core form of meta-dynamism*.
*Adjunct 1B: Core Meta-Dynamism as Pre-Reflective Core Consciousness:* The presence, within a process, of that core meta-dynamism constitutes the possession by that process of some group of *core feelings* (which do not involve the use of concepts). Thus, these feelings are present within all consciousness. They accordingly are said to form *core consciousness.* Core consciousness is thus entirely non-reflective.
*Adjunct 1C: Further Specification of Core Consciousness:* The core feelings include a crude *feeling of the experience’s own continuing existence*, however brief. The feeling is entirely *non-egoic*. Furthermore, especially if the “suitability” mentioned in 1A is vacuous (i.e., no further conditions are placed on the meta-dynamism) then this feeling constitutes core consciousness by itself.
*Adjunct 1D: Beyond Core Consciousness:* As an enrichment of the core feeling of this-experience’s-continuing-existence in adjunct 1C, consciousness of something X (whether X is internal to the experience or external) involves (at least pre-reflectively) experiencing that-particular-X-ness-in-this-very-experience.
*Adjunct 2A: Quasi-Isolatability of Core Meta-Dynamism:* A process can consist almost entirely of the core meta-dynamism, i.e., it largely lacks other dynamism (base-level dynamism or other forms of meta-dynamism), and it has a relatively very low degree of interaction with its physical environment. Thus, the process consists *almost-purely of core meta-dynamism*.
*Adjunct 2B: Pure Coreness of Meta-Dynamism as Pure Coreness of Consciousness:* The closer an episode of consciousness is merely to being composed just of the core meta-dynamism, the closer it is to consisting just of core consciousness. Very-close cases can be called *almost-pure core consciousness*, or *almost-pure consciousness* for short. Moreover, at some degree of closeness the consciousness becomes entirely non-conceptual—it is non-reflective and consists just of feelings that are little more than core consciousness.

The point of Adjunct 1A is that the Sufficiency Conjecture in itself does not imply that all conscious processes share a *common* form of suitable meta-dynamism. The adjunct stipulates a common core.

The point of Adjunct 1B is to attach particular, core phenomenal significance to the core meta-dynamism, where the phenomenality consists purely of certain concept-free feelings. What these feelings are is not yet specified.

Adjunct 1C rounds out 1B by further specifying core consciousness. It makes a form of temporal consciousness be part of, and perhaps even the whole of, core consciousness. This form is basic and pre-reflective. It does not, for instance, involve a concept of time durations, successions, etc. Rather, it is a direct experience of the temporally local durating (continuing). The postulation of a fundamental feeling is parallel to the postulation of an “ur-quale” in the AI-related proposal of Perlis [44].

Adjunct 1D addresses the topic of what it is to be conscious *of* something, a more extensive treatment of which is beyond the scope the present article. However, it is useful to codify here the ideas in Section 2.1 about reflexivity in consciousness-of, that for instance in being conscious of a rose one is experiencing particular-rosishness-in-this-current-experience. The adjunct also states, in effect, that the core feeling of own continuing existence postulated in Adjunct 1C is in particular the core of all consciousness-of.

The point of Adjunct 2A is that 1A does not claim that it is possible for little else to be happening in a process other than the core meta-dynamism. As far as 1A is concerned, the core meta-dynamism might always have to be embedded in rich dynamism of other sorts. Adjunct 1B commits to the “else” being possibly slight. In stops short of claiming that there can be nothing else at all, or that there can be absolutely no interaction with the physical environment.

The point of Adjunct 2B is to translate the possibility of overtly physical quasi-isolation of Adjunct 2A into the possibility that a conscious episode is little more than core consciousness. The less internal physical activity there is other than the core meta-dynamism, the less internally generated conceptual content (if any at all) there is, and the less internally generated feeling there is. The more that the episode physically consists just of core meta-dynamism, the less externally related conceptual content there is and the less externally prompted feeling there is. Moreover, it is possible for an almost-pure consciousness to lack any conceptual content.

We thus end up with the proposal that there can be *largely contentless* episodes of consciousness—the almost-pure episodes, which in more extreme cases can exist of little more than the core feelings. We identify extent of phenomenal purity with extent of content. We are careful here to distinguish between, but to account for both conceptual content and content in the form of feelings. We are also careful to distinguish between purity and mere coreness. To bring out something already implied, and abstracting away from MDyn’s particular proposals, a notion of “core” consciousness need not of itself imply more than the proposition that that type of consciousness is present within all consciousness: it need not imply that a conscious episode can consist of little more than core consciousness, let alone consist literally of just the core type. Equally, a claim that a conscious episode can be (almost) contentlessness or (almost) pure (which could be different matters in a theory other than MDyn) need not imply that there is any common core to consciousness: there may be different conglomerations of basic content in different versions of quasi-contentlessness or quasi-purity.

I should also note that, in the literature, “pure consciousness” can mean something more like core consciousness above. For instance, Husserl [10] in his *Ideas: General introduction to pure phenomenology* (pp. 111–146) uses the term in addressing episodes of consciousness in their own right, concentrating on general features of consciousness such as unity, and putting aside connections to the external world, even when the consciousness is highly contentful of thoughts and feelings (including about the external world). But this does not imply that, deprived of (most of) such content, the aspects of consciousness referred to can exist (almost) all by themselves. So the “pure” consciousness is not necessarily pure in this article’s sense.

Furthermore, lurking here is the idea of “minimal” consciousness. While MDyn’s core consciousness could be called minimal in a sense, MDyn does not claim that core consciousness can exist by itself as a minimal type of consciousness episode, any more than an apple core can grow on a tree all by itself and therefore be a minimal apple. A conscious episode probably needs always to include a little more than the core. Core consciousness is probably at best a lower limit rather than a minimum. So an exactly core meta-dynamic episode might only be able to exist in a universe consisting of nothing but it. But it may be that consciousness can (in principle) get arbitrarily close to being just core consciousness, so there is no free-standing minimum at all in principle. Equally, a theory that did propose a minimal free-standing type of consciousness could conceivably propose several such, even if there is a common core: they would just be different minima that stand away from coreness.

There is also the separate question of minimality with respect to degree of consciousness, when consciousness is fully or partially graded. The Adjuncts do not constrain the (non-zero) degree of consciousness in core or almost-pure consciousness. Thus, in particular, there could be qualitatively episodes of almost-pure consciousness that differ only in the degree of consciousness (or individual degrees for various parts of the experience).

Henceforth, when the Sufficiency Conjecture is in the picture, we will usually assume all the Adjuncts are added, to form MDyn’s main proposal. Discussion Section 5.2 discusses the issues further.

### 2.9. Conceptual Succession of Levels of Meta-Dynamism

At any point in a conscious process (and perhaps at some or all points in certain types of non-conscious process), past dynamism in a stretch of the process meta-dynamically affects state at that point. But the same observation applies to earlier points in the process. So the dynamism meta-dynamically affecting state at a point includes that very same sort of meta-dynamism. So the mentioned meta-dynamism is to that extent *meta-meta*-dynamism—and *that* very sort of dynamism is present throughout earlier stretches of the process. And so forth, up an infinite ladder of “levels of meta”. Furthermore, if we allow a system to refer to current dynamism as well as past dynamism, we get a similar succession of levels.

However, thinking of this as composing an infinite ladder of levels of meta is just a conceptual view, and does not mean that physical reality somehow contains a troublesome infinite stratification. Rather, MDyn proposes that there is meta-dynamism that simply has no defined “height” in terms of levels, and the dynamism that takes part on the cause or effect side in a given instance of such meta-dynamism itself has no defined height.

## 3. Formal Treatment

This section attends to the formal treatment of dynamism, meta-dynamism and meta-dynamic auto-sensitivity. Up to and including Section 3.11, it addresses these matters as general possibilities, i.e., as something not necessarily related to consciousness. Section 3.12 onwards then specialize to meta-dynamism in consciousness. Section 4 will go into some additional considerations about the formal treatment.

This article’s main interest is in *time-hopping* meta-dynamism (see Section 1.5), as opposed to base-level dynamism and any non-time-hopping meta-level dynamism. This is because the philosophical side of MDyn proposes that at any given, current moment *t* a conscious process is meta-dynamically affected by dynamism *over some (non-empty, non-punctate) time-interval* within the process’s history up to *t*, where, moreover: the dynamism over that interval have its effect as a *unified entity*, not for instance as independent effects of instantaneous instances of dynamism within the interval. As noted earlier, the time-hopping nature of MDyn’s physical side means that it introduces a temporally non-local form of physics. The time-hopping is a real feature of the physical world in MDyn’s view, not (for example) just a mathematical way of looking at things.

The section proposes a way in which classical physical laws can be modified to explicitly mention dynamism as well as ordinary quantities, and thereby to display meta-dynamic constraints. The modifications to laws also imply similar modifications to the equations of motion or other change that describe some specific physical system, be it a particular pendulum or a particular brain, and that are derived from the general laws. For most of the section, we will talk explicitly only about laws, with the understanding that derived, specific equations of change are being implicitly addressed as appropriate. But in Section 3.11, Section 3.15 and Section 3.16 we will switch to considering some toy systems described by specific equations of change, without commenting on the laws that the equations would be derived from. Section 4.1 discusses some of the provisions that are desirable in laws in order to enable the derivation of such equations of change.

I emphasize that this article does not propose particular modifications of any particular law. My intent is just to show the general nature of the components of meta-dynamic laws and derived equations of change that I envisage in order to give a possible precise, fundamental basis to MDyn.

The mathematical treatment incorporates some simplifying assumptions and some ways of making the philosophical ideas more specific. All the simplifications and specifications are subject to change in the future. A salient one is that I currently assume classical continuous spacetime, and spacetime coordinates [x,t] relative to some arbitrary origin. I will usually summarize [x,t] as being a (spacetime) location *l*. Furthermore, it is convenient to say l=[x(l),t(l)].

For a spacetime region *R*, Times(R)={t(l)|l∈R}. For maximum flexibility, I use the term “[spacetime] region” just to mean a non-empty set of locations in spacetime, with no further restriction, but regions of interest will usually in fact be restricted in the text as appropriate, e.g., restricted to be open, connected sets of locations.

### 3.1. Laws: Initial Comments and Ordinary Laws

The mathematical treatment centres on how meta-dynamism might be reflected in *meta-dynamic* laws as couched in mathematical equations, and how the notion of meta-dynamic auto-sensitivity of processes might thereby be accommodated. Meta-dynamic laws could be modifications of existing physical laws or entirely new laws. A main feature of the meta-dynamic elements in the laws is that they make the laws non-Markovian and in a particular sense *temporally non-local*, so that the dynamics of the world intrinsically become temporally non-local, going against a prevailing tendency in physics [28]. Adlam [28] goes through a variety of ways in which physics has already been proposed to be, or could or should become, temporally non-local, including by means of non-Markovian laws. MDyn therefore brings in considerations about what information determines states. Furthermore, because of the temporal non-locality, and also because of the deep connections between dynamism and time-flow, the approach also implicitly connects to the topic of entropy insofar as this is related to time [58].

In order to avoid having to address forms of temporal non-locality other than MDyn’s, I will use what I will call *ordinary* laws as my starting point for proceeding towards meta-dynamic laws. Ordinary laws are non-meta-dynamic and also in all other respects temporally local, i.e., the mathematical equations in which they are couched explicitly or implicitly involve a time-parameter *t* denoting an arbitrary time instant, and all physical quantities mentioned in the law are evaluated at *t*. An application or instantiation of a law in a particular case is therefore always about the state of the world at one instant. An ordinary law does not explicitly put quantity values that apply at different instants in relation to each other. The implicit way such relations arise through the use of time derivatives will be discussed shortly.

As another current simplification I will also put aside the spatially non-local effects and possibly inherently stochastic aspect of physics, and essentially proceed by using classical physical laws such as F=ma (as applied to idealized point objects) as a model. This limitation of spatial locality should be relatively straightforward to surpass in future possible development of MDyn in a quantum-physical framework, as the main thrust of MDyn is not concerned with patterns of state or behaviour across space as opposed to across time. In fact, for brevity, I sometimes talk about the whole world state at a particular time, across all spatial positions, or across all spatial positions in a given process or system.

I will therefore take a given application of an ordinary law to be about world state at just one point in space as well as time, i.e., one “current” punctate location l=[x,t] in spacetime. The aspects of world state addressed by an ordinary law are the values at *l* of familiar physical quantities such as forces, potentials, masses, accelerations, electric fields, etc., and derivatives of such quantities with respect to time or space. I will call such aspects of the world the “ordinary” aspects of the world to distinguish them from the reified dynamism that MDyn includes in world states (more on this below).

### 3.2. Ordinary Laws Continued, and Scenario 0

I will now make some make some familiar or otherwise unsurprising points about ordinary laws in order to set the stage for talking about meta-dynamic laws.

Time derivatives in an ordinary law can be viewed as *implicitly* alluding to the state of the world at times surrounding a given time of application of the law. A simple example is the classical F(l)=m.a(l) for an idealized point object with constant mass *m* at spacetime location *l*. The use of acceleration *a*, in its nature as the time derivative of velocity and second time derivative of position, makes the application of the law implicitly allude to the velocity and more indirectly the position of the object at times around t(l), although the application is explicitly only about force on and mass and acceleration of the object at *t* itself.

But this implicit allusion only exists because of an added, usually tacit, assumption; namely that the states of the world at surrounding times are *consistent with the state at t*. With this assumption in place, the law applications at *t* implicitly allude to, and thereby mathematically constrain, the evolution of world state up to and including *t* and the evolution from *t*. From now on, I will be mainly concerned with the past evolution, up to *t*. This evolution, though of course time-spanning, can itself be said to be entirely *[arbitrarily] temporally local* in the sense that the world state at some time $t^{\prime }<t$ affects the world state at *t* entirely via that continuous evolution. This point resides in the fact that no particular time $t^{\prime }$ is important here. The state at *t* can equally be thought of as arising from world state at any time strictly between $t^{\prime }$ and *t*, and therefore from the world state at times $t^{\prime \prime }$ that are arbitrarily recent (arbitrarily close to *t*). For no such time does the determination of the state at *t* require consideration of states before $t^{\prime \prime }$.

So, aspects of world state at different times are mutually constrained by virtue of, and only by virtue of, continuous consistent evolution in between those times, hinging on the need for the values of quantities at one time to be consistent with inherently related values at arbitrarily recent times. (For brevity I will mostly omit mention of the need for consistency of state evolution with boundary conditions.)

The use of the term “continuous” in “continuous evolution” is only intended to refer to the non-discreteness of the time dimension. There is no necessary assumption in this discussion that quantity values always change continuously.

Any type of constraining of states with respect to each other at different times can be said to be the *diachronic* dimension of constraint. So far, the only diachronic constraint at hand consists in the consistent, continuous, arbitrarily temporally local evolution as described above. There is also the *synchronic*, dimension of constraint, namely the explicit mutual constraining between ordinary state quantities at any one time *t*, as stated by the laws. Again, a basic example is the mutual constraining of the values of force, mass and acceleration at the time *t* of *l* in F(l)=m.a(l). The laws taken as a whole are assumed to determine all the synchronic constraining that needs to be described for evolution to be determined with the help of the diachronic constraints.

I label this scenario of consistent evolution in accordance with ordinary laws as “Scenario 0”. In the next subsection, we consider a variant non-meta-dynamic scenario, Scenario 1. After that, meta-dynamism will be added to get a fuller scenario.

### 3.3. Scenario 1: Ordinary Laws and Reified Dynamism

Here we vary Scenario 0 slightly by including reified dynamism in the world. However, we do not yet let laws explicitly refer to dynamism—they remain ordinary. So, though there is dynamism in the world, and this is what constitutes the very evolution of world state, the laws are not explicitly *about* dynamism, and merely display the synchronic constraints (between ordinary quantities) inherent within the dynamism. Discussion of this scenario helps us to understand what happens when laws are allowed to refer to dynamism, in the full scenario.

Dynamism is now part of world state, so is now itself part of what it is that evolves by dynamism. Dynamism and ordinary state at a given time *t* determine the dynamism and world state at future times. Whatever dynamism is, the dynamism at a location should by itself logically determine the overall law-abiding, evolutionary constraining of all quantities directly relevant to that time, including dynamism itself.

I assume that the mere reification of dynamism, without including laws that mention it, has no effect on what evolution of ordinary state arises, so the ordinary-state evolution in the current scenario is identical to that in Scenario 0. Dynamism is what accounts for why the universe evolves at all, but the changes that thereby arise are, in this scenario as in the one above and those below, completely described by ordinary laws together with the need for evolutionary consistency across time that we have discussed. No law in the current Scenario states a constraint between dynamism and ordinary state, so the only changes to dynamism in the evolution are such that it always implies the constraining inherent in diachronic consistency and the synchronic constraints in laws between ordinary quantities.

It is now useful to introduce the following notation.

O(l) denotes the conglomeration of ordinary state quantity values at a spacetime location *l*. What quantities this conglomeration covers is left unspecified here—it can vary across different completed physical theories. It includes all ordinary quantities, including relevant derivatives, that are needed in the laws of the theory.
D(l) denotes the dynamism at spacetime location *l*, and D(R) for a spacetime region *R* denotes the holistic dynamism region over *R* (recall Section 2.3). We will deploy dynamism regions below, and for the moment deal only with the dynamism at a location. The dynamism at a location defines, for the moment, just the constraining that involves state at *l* and is exerted by the arbitrarily temporally local continuous evolution and by the laws.
TotalState(l) denotes the combined ordinary/dynamism quantity value conglomeration for *l*, the pair (O(l),D(l)). A combined value with this structure, even if it is a hypothetical value, is called a *total-state.* The *actual* total-state at *l* is TotalState(l). Given a total-state *T*, $T_{o}$ is its ordinary-state part and $T_{d}$ is its dynamism part, i.e., $T=(T_{o},T_{d})$. So in particular, $\mathrm{TotalState}\left(l\right)=\left(O\left(l\right),D\left(l\right)\right)=\left(\mathrm{TotalState}{\left(l\right)}_{o},\mathrm{TotalState}{\left(l\right)}_{d}\right)$.

### 3.4. Meta-Dynamic Laws

Meta-dynamic laws are ones that, at least in part, explicitly *refer to* “dynamism quantities”—aspects/components of, or quantities associated with dynamism itself—via mathematical expressions of some sort, in the same sense that ordinary laws refer to ordinary quantities. Just as ordinary laws thereby can be thought of as *displaying* dynamism that is concerned with those referred-to ordinary quantities, meta-dynamic laws *display* meta-dynamism that is concerned with the referred-to dynamism. They display ways in which dynamism quantities affect and are affected by other dynamism quantities or ordinary quantities. This affecting is meta-dynamic.

However, a complication is that referred-to dynamism can itself be meta-dynamism, so a meta-dynamic law can both refer to some meta-dynamism and display some meta-dynamism, and indeed perhaps even the same meta-dynamism, as we will see. Note also that the meta-dynamic laws can refer to ordinary quantities as well as to dynamism.

The meta-dynamic laws envisaged in most of this article will still have a single-spacetime-location parameter *l*. The differences from ordinary laws will reside most importantly in their ability to explicitly refer to dynamism, both dynamism at *l* and earlier dynamism regions. Past values of non-dynamism quantities can also be referred to, but we will only make a certain, limited use of this here, in sub-expressions allowing laws to refer to past dynamism regions.

There are two broad possibilities for a meta-dynamic law: (1) it is a directly modified form of some existing law, e.g., a modified classical law, a modified Schrödinger equation in quantum theory, or potentially (when we eventually attend to relativistic concerns) a modified Einstein field equation in general relativity, with for instance extra terms that refer to dynamism; or (2) it is not such a modification—it is a completely new law. Under (2) we could have laws that state relationships between ordinary state and dynamism, and/or laws that just refer to dynamism, and not ordinary varying quantities, though perhaps involving existing physical constants. This article talks mainly in terms of (1) but the points made are generally extensible to (2). In (1) I include not only possible changes to existing, standard laws, but also changes to already non-standard laws as proposed by other researchers.

A schematic illustration of the proposed way of modifying laws is as follows. Consider, for instance, an ordinary law with the structure:

u(l)=v(l)w(l)

A modified version might then have the structure

$$u\left(l\right)\;=\;\Gamma_{1}\left(l\right)v\left(l\right)w\left(l\right)+\Gamma_{2}\left(l\right)$$

(or simpler versions with only one of the Γ components), where the Γ expressions refer to dynamism in ways to be described shortly. There are additional, related possibilities for placing Γ expressions. For example, some existing term in a law could be raised to a power involving such an expression, or some new operator somehow involving a Γ expressions could be applied to a term. However, for simplicity of presentation I will confine attention to multiplication by factors such as $\Gamma_{1}\left(l\right)$ above and addition of terms such as $\Gamma_{2}\left(l\right)$.

The $\Gamma_{i}\left(l\right)$ expressions as written above are just schemata, standing here for particular, detailed *gating expressions* in the particular laws. These are particular expressions about ordinary state O(l) at *l*, dynamism D(l) at the *current location l*, or dynamism D(R) over *past spacetime regions* *R* are needed in the particular law. An *R* will always extend across some non-punctate range(s) of times and spatial places, and be “past” in the sense that the times in it are no more than the current time t(l). However, in examples, the regions will always have times strictly before t(l), although the region may *temporally abut l* in the sense that it contains times arbitrarily close to t(l):

**Definition** **1.** *Given a spacetime location l, a temporally abutting open spacetime region at l is an open region A such that Times (A)=(t,t(l)) for some t<t(l).*

Neither the punctate dynamism at a location nor holistic dynamism over a region are envisaged to be numerical quantities (whether scalar, vector, etc.), and may have many separable aspects, arranged in complex structures. So, in order to have a quantitative interface between dynamism as such and the rest of a law, we suppose that there are *[dynamism] valuation* functions, typically illustrated by the symbol β below, usable within gating expressions, that when applied to an instance of punctate or regional dynamism return quantitative values defined by the details of that instance.

Derivatives (notably, with respect to time and space) of valuation functions can be used, and will themselves for be classed as valuation functions. This allows, for one thing, the evolution of dynamism to be lawfully constrained in much the way that the evolution of ordinary quantities is.

To refer to punctate dynamism at the current location, *l*, a gating expression would use the expression D(l). To refer to a past dynamism region, a gating expression would use D(R) where R is a *region-valued expression*, identifying a spacetime region. Thus, the current main way for a law to be meta-dynamic is to contain, within a gating expression or as the whole of one, an element such as

β(D(R))

A region-valued expression R, as currently envisaged, does not identify a region by quantitatively specifying locations as such. Rather, one main way, and the only one used in examples in this article, is to identify a region by some condition it must satisfy taken as a whole, or by some condition that world state at each of its locations must satisfy. To take the latter first, a main proposed form for a region-valued expression R is

$$R_{\mathrm{loc}}\left[C\right]\left(l\right)$$

where C is an expression stating the condition that each location in the region must satisfy (C is a Boolean function on locations). The displayed expression then delivers the set-wise maximal region that satisfies the following:

- All of its locations $l^{\prime }$ make $C(l^{\prime })$ TRUE;
- It is *open*;
- It *temporally abuts* *l*; and
- The region plus location *l* is *connected*.

An important possibility, key in some examples below, is that the delivered region is empty.

The delivered region is also the union of all regions satisfying that bulleted list. In particular, in such a union all locations are connected at least via *l*.

But note that the region itself, without *l*, may not be connected. The connectedness-with-*l* requirement is included because otherwise the region might be arbitrarily spatially distant from *l*. So it is a minimal spatial-locality requirement. Furthermore, in future developments, we may wish to ensure that the region is contained within the past light-cone of *l*.

There could be other region-delivering functions that impose a different list of requirements, but this article only uses the above list.

Currently at least there are no restrictions on the structure, complexity or content of the expression C. In particular, it can itself include gating expressions, and the latter may themselves include region-valued expressions. We will make good use of such possibilities below.

The other main way of specifying a spacetime region *R* is by a condition H on the region as a whole. We therefore have expressions such as

$$R_{\mathrm{reg}}\left[H\right]\left(l\right)$$

In this article, this delivers the set-wise maximal open region temporally abutting *l* that satisfies condition H and is connected once *l* is added. However, caution is needed here because there may be no unique maximal such set—the union of sets satisfying H might not satisfy H, for some types of H. Again, there may be variant sorts of region-returning function that appeal to a global condition H.

Naturally, a location-wise condition C is technically equivalent to a whole-region condition H that says that C must apply at every location in the region, but it is formally easier and clearer if we use a special syntax for the location-wise case.

Finally, the current formalism currently does not allow for a past ordinary-state value to be referred to in a gating expression other than in the conditions C and H within region-valued expressions. It also has no provision for referring to dynamism values at past individual locations, except again within such conditions.

Putting various possibilities together, a meta-dynamic law that varies the above imagined standard ordinary law, u=vw, might have a form such as

(1) $$u\left(l\right)\;=\;q_{1}\beta_{1}\left(D\left(R_{\mathrm{loc}}\left[C\right]\left(l\right)\right)\right)v\left(l\right)w\left(l\right)+q_{2}\beta_{2}\left(D\left(l\right)\right)$$

where in principle C might be a simple condition on an ordinary quantity *r*, such as $\lambda l^{\prime }.r\left(l^{\prime }\right)>0$. (This lambda-expression denotes the function on locations $l^{\prime }$ that delivers TRUE when $r(l^{\prime })>0$, FALSE otherwise.) Expressions $q_{1}$ and $q_{2}$ may be composed of ordinary quantities. The gating expressions are $q_{1}\beta_{1}(D\left(R_{\mathrm{loc}}\left[C\right]\left(l\right)\right)$ and $q_{2}\beta_{2}\left(D\left(l\right)\right)$. (Where the boundaries of a gating expression are is a matter of which ordinary law the meta-dynamic law has been derived from, and is otherwise a matter of convenience in discussion, subject to all dynamism-references being within gating expressions.)

A meta-dynamic law application at current location *l* that refers to past regions of dynamism places a constraint between such dynamism regions and other quantities referred to in the law, including ordinary quantities at the current location. This is a diachronic constraint in the terminology of Section 3.2, but is of an additional type that is not arbitrarily temporally local: we cannot fully determine current world state just by appealing to the evolution of state since some arbitrarily recent time, because there is some sort of dependence on world state back to the beginnings of the referenced dynamism regions (or further back, as made clear in Section 4.2). I use the term *time-hopping meta-dynamism* for the sort of dynamism that involves the new diachronic constraints, which will also be said to be time-hopping. I use this term even if the spacetime region occupied by the referenced dynamism region temporally abuts *l*. I assume that time-hopping constraints only arise if some law has explicit time-hopping features, i.e., refers to past dynamism regions.

### 3.5. Formalities Concerning Dynamism Regions

**Definition** **2.** *The home region Home(Y) of a region of dynamism Y is the spacetime region it occupies.*

**Definition** **3.** *For any regions of dynamism $Y_{1}$ and $Y_{2}$, $Y_{1}$ is [contained] within$Y_{2}$ or a subregion of $Y_{2}$, notated $Y_{1}\subseteq Y_{2}$, if and only if Home$\left(Y_{1}\right)\subseteq Home\left(Y_{2}\right)$.*

**Definition** **4.** *Given a region of dynamism Y, the set of times in Y is Times(Y)=Times(Home(Y)).*

**Definition** **5.** *Given a spacetime location l and region of dynamism Y, Y is past for l, or a past region of dynamism for l, if and only if ∀t∈Times(Y),t<t(l).*

**Definition** **6.** *Given a spacetime location l, a temporally abutting past region of dynamism for l is a past region of dynamism Y for l such that Home(Y) temporally abuts l.*

### 3.6. Meta-Dynamism in Current Dynamism Values

When a law (with location parameter *l*) refers to a dynamism region that is past (with respect to *l*), it is thereby *displaying* the time-hopping meta-dynamism consisting of that region (typically) affecting current state, not *referring to* it, using the distinction made in Section 3.3. However, matters now become complex because that meta-dynamism is also part of current punctate dynamism D(l). So, if a law refers to current dynamism, via D(l), it may therefore (depending on what valuation function is applied) implicitly refer to time-hopping meta-dynamism if any, included within D(l). In this sense, a meta-dynamic law can both refer to and display current time-hopping meta-dynamism, and a law as applied to a particular location *l* can even refer to the current instance of the time-hopping meta-dynamism displayed in that very law.

A similar thing can happen with non-time-hopping meta-dynamism. To make this clear, assume that no law refers to past regions of dynamism, so there is no time-hopping meta-dynamism, but some law refers to D(l). D(l) includes any meta-dynamism constituted by the law-displayed interacting of (aspects of) dynamism with ordinary quantities (and aspects of dynamism with each other). This is just in the same way that it includes any *non*-meta-level dynamism constituted by the law-displayed interacting of ordinary quantities with each other.

When a law that uses D(l) thereby indirectly refers to the meta-dynamism included within D(l), that meta-dynamism is put into (typically synchronic) constraint with other quantities in the law, giving a sort of meta-meta-dynamism. And so forth.

In sum, meta-dynamic laws can both refer to and display dynamism, including time-hopping and non-time-hopping (meta-...meta-)meta-dynamism as special cases, and can do so in such a way that there is complex reflexivity in the sense that meta-dynamism displayed by a law may also be referred to by one or more laws, perhaps even the same law.

A special matter arises from the point in Section 2.3 that a dynamism region is holistic in that it is not just the plurality of the punctate dynamisms over all its locations. Correspondingly, we postulate the following:

**Regional Holism of Time-Hopping Meta-Dynamism:** The direct constraining, if it exists, of state at *l* by a given past region of dynamism *Y* by virtue of *Y* being referred to in laws is not, in general, determined by, or determining of, such constraining of state at *l* by any proper subregions of or locations in *Y*.

In particular, no matter how many proper subregions *Z* of *Y* constrain state at *l*, *Y* as a whole may exert further constraint on that state. Of course, the *Z* regions provide an indirect way in which *Y* constrains state at *l*, since fixing *Y* fixes each *Z*, but there can be direct “added value” exerted by *Y* as a unit. But an opposite point is that *Y* as a unit may not directly constrain state at *l* at all. Moreover, the direct constraining, if any, of state at *l* by a proper subregion *Z* of *Y* is not determined by the constraining or otherwise of that state by *Y* as a unit.

### 3.7. Gating of Involvement of Dynamism in Laws

Gating expressions serve not only to allow laws to refer to dynamism, but also to “gate” or modulate the extent to which that reference has an effect on other quantities, i.e., to amplify or attenuate the strength of the meta-dynamism to whatever extent and in whatever way might be appropriate. Recalling a point in Section 1.7, this is important in order to ensure the effective invisibility of new laws or law-features outside specific sorts of system, notably conscious brains.

For a varied form of an existing law to fulfil that condition, we need the value of an additive gating expression such as the $\Gamma_{2}$ above to be, over large regions of spacetime, zero or a tiny value (scalar or otherwise) relative to other values involved. Similarly, the value of a multiplicative gating factor such as the $\Gamma_{1}$ above to be (dimensionless) 1 or a value extremely close to it. I will say that being sufficiently close to these 0 or 1 values, respectively, makes a gating expression *[effectively] invisible*, while other values make it *visible*. If a gating expression value is exactly 0 or 1 as appropriate, I will say it is an *absolutely invisible* value, and the gating expression in question is absolutely invisible (at the current location).

In general, even absolute invisibility does not mean the ordinary state and dynamism state mentioned by the gating expression are protected from taking part in interaction with the ordinary quantities elsewhere in the law: those quantities may be viewed as helping to constrain the gating expression to be absolutely invisible and thereby constraining the state items it mentions.

As a special case, it may be that only the ordinary state quantities in a gating expression affect whether the expression is visible or not—that is, it is only ordinary quantities that provide switching between invisibility and visibility. Another special case is that, when a gating expression is visible, its value at a particular *l* may depend only on mentioned dynamism—i.e., the gating expression is sensitive, when visible, only to the dynamism, not ordinary state.

A combination of these two special cases is that the ordinary state provides all the switching and only the switching. As a simple example, in the schematic example (1) we have a gating expression $q_{2}\beta_{2}\left(D\left(l\right)\right)$. Here $q_{2}$ could be entirely ordinary and only lending the whole gating expression a significant size in special circumstances. Note also that here, when $q_{2}$ is exactly zero, no constraint is exerted on or by D(l) via the law: any conceivable instance of punctate dynamism will fit in with other quantities.

A more implicit way in which gating of meta-dynamism, when time-hopping, can happen is that a region-valued expression might normally return an empty dynamism region because the punctate condition C or whole-region condition H is only exceptionally satisfied. With a suitable valuation function applied to the delivered dynamism region, emptiness of the region will give a value of zero for that function, and can be regarded as a lack of the time-hopping meta-dynamism that would otherwise have existed. This is amply illustrated below.

### 3.8. Dynamic Intensities and Regional Holism of Intensity

D(l), the overall dynamism at *l*, has various aspects, corresponding to different sorts of interaction among ordinary quantities and dynamism quantities. I now assume that some or all of such aspects have a non-negative real-valued *intensity* at *l*. I leave to further work of the question of the precise range of aspects that have an intensity and how an intensity is defined, and how it relates to the specific laws included in the physics. Instead, I assume a minimum required for the developments and comments below.

The intensities are objective features of the physical world, and as such can potentially be used in laws via intensity-returning dynamism valuation functions.

The most straightforward intensity is that of a location’s punctate dynamism as a whole. (Other intensities will be mentioned below.) This overall intensity is the intensity with which the world state at location *l* (including the dynamism itself) is being dynamically affected, taking into account all types of dynamic effect, whether meta-level or not and whether time-hopping or not, and irrespective of which regions of dynamism are involved in the time-hopping case. More selective notions of intensity will shortly be mentioned.

The overall intensity is not, intuitively, a question of the “amount” of dynamism at *l* and therefore related, for instance, to how large the spacetime regions featuring in the time-hopping aspects of the dynamism. Rather, the intensity is intuitively like a rate of change of overall state at *l* with respect to changes in the world at earlier times. Since there can be many possible aspects of the world that could be changed, I assume the intensity is a maximum of the “rates of change” over the possible types of change that could be made. I cannot yet propose a more detailed and specific analysis of intensity, given that dynamism is a complex matter and its structure has not been adequately elucidated.

I will also assume that a dynamism region has intensity values. These are holistic in not necessarily having any simple relationship to the intensities of sub-regions or locations within the region. This respects the holism of dynamism regions themselves, as emphasized in Section 2.3. Note carefully that a dynamism region’s own intensities are different from the intensity with which it meta-dynamically affects later state.

The following intensity functions are useful for current purposes.

κ(D(l)) denotes the overall intensity of the dynamism at location *l*, taking into account all types of dynamism: base-level or meta-level, and involving any type of quantity.
κ(D(R)) denotes the overall, holistic intensity of the dynamism region D(R).
$\kappa_{B}\left(D\left(l\right)\right)$ denotes the base-level dynamism at spacetime location *l*, i.e., taking into account only those types of dynamism that involve the constraining of, merely, ordinary physical quantities with respect to each other, irrespective of which ordinary quantities they are.
$\kappa_{B}\left(D\left(R\right)\right)$ denotes the holistic intensity of the base-level aspect of dynamism region D(R).
$\kappa_{B,q}\left(D\left(l\right)\right)$ denotes the base-level dynamism involving ordinary quantity *q* at spacetime location *l*, i.e., taking into account only those types of dynamism that involve the constraining of *q*, either by itself, e.g., constraining *q* and ∂q/∂t to be consistent with each other, or with respect to other ordinary quantities.
$\kappa_{B,q}\left(D\left(R\right)\right)$ denotes the intensity of the base-level, *q*-involving aspect of dynamism region D(R), where *q* is an ordinary quantity.
$\kappa_{M,TH}\left(D\left(l\right)\right)$ denotes the time-hopping meta-dynamism at spacetime location *l*, i.e., covering all dynamism that involves constraint between past regions of dynamism (considered as units) and state at *l*.
$\kappa_{M,TH}\left(D\left(R\right)\right)$ denotes the holistic intensity of the time-hopping meta-dynamic aspect of dynamism region D(R), i.e., covering all dynamism linking past regions of dynamism (considered as units) to state at at any location within *R*. Note that these past regions regions need not have home regions that spatially intersect *R*.
$\kappa_{M,TH}\left(Y,l\right)$, denotes the intensity of the time-hopping meta-dynamic constraint, if any, of dynamism region *Y* with respect to state at *l*. Note: Such an effect is part of D(l). All aspects of TotalState(l) are lumped together here as regards considering *Y* to constrain that state.

Some particular points to note about intensities are as follows.

- The above functions do not exhaust the possible range. For instance, it may be useful to have functions that measure intensities of non-time-hopping meta-dynamism. Functions that measure the meta-dynamic interaction of ordinary quantities with dynamism may be useful.
- Values are zero if an effect of a required type is missing. For instance, $\kappa_{M,TH}\left(Y,l\right)=0$ if *Y* does not meta-dynamically affect state at *l*. Furthermore, when *Y* is empty (i.e., its home region is empty) then $\kappa_{M,TH}\left(Y,l\right)=0$. Similarly, the other regional dynamism intensities are zero when the region is empty.
- Intensities of dynamism regions are not systematically dependent on the spatio-temporal sizes of the regions, and similarly $\kappa_{M,TH}\left(Y,l\right)$ is not systematically affected by the size of *Y*. A particularly important case of this is when *Y* temporally abuts *l*. No matter how little a temporally abutting *Y* goes back in the past from *l*, and how small spatially it is, $\kappa_{M,\mathrm{TH}}\left(Y,l\right)$ may be greater than zero. Furthermore, we do not exclude the possibility that there is positive lower bound on such values.
- All intensities are at least zero, but currently we impose no systematic upper bound. In toy examples below we occasionally assume an upper bound of 1, but this could be replaced by the applying to intensities a function that limits the values to [0,1].
- We assume that the holistic intensity of a particular sort for a region is non-zero if and only if the punctate intensity of that sort is non-zero for some location in the region. For instance, $\kappa_{B}\left(Y\right)>0$ if and only if we have $\kappa_{B}\left(l\right)>0$ for some l∈Home(Y).
- For that assumption it follows that: the holistic intensity of some sort for an (open) region is non-zero if and only if it is non-zero for some proper sub-region.

### 3.9. Processes in General

Here I present a notion of a process, convenient for purposes of formulating conscious processes in MDyn but also allowing consideration of meta-dynamism within processes more generally.

**Definition** **7.** *A process P is a triple (Home(P),ProcStates(P),D(P)) where*

*Home(P) is an open region of spacetime, called the home region of P.*

*ProcStates(P) is a function from Home(P) into the set of conceivable total-states.*

*D(P) is a dynamism region that could conceivably exist, has home region equal to Home(P), and is compatible with ProcStates(P).*

Recall from Section 2.3 that a region of dynamism determines the punctate dynamisms within it, and vice versa. Accordingly, part the compatibility mentioned in the last clause of the definition is that the dynamism portions of the total-states are as determined by D(P), and vice versa. Technically, therefore, the D(P) component is redundant, but is included for convenience.

**Definition** **8.** *For a process P, Times(P)=Times(Home(P)).*

**Definition** **9.** *A process P is actual if and only if ∀l∈Home(P),ProcStates(P)(l)=TotalStates(l).*

That is, the states of the process are the actual states over the home region. By the compatibility requirement, this requires that D(P)=D(Home(P)), i.e., the overall dynamism in the process is the actual holistic dynamism of the home region.

Because the home region is merely required to be an open set of locations, this definition of processes is extremely general, allowing the region to be a set of disconnected subregions arbitrarily far from each other in space and time. This freedom is useful for certain processes of discussion. However, further restrictions will be placed on the home region when we come to conscious processes below.

The definition omits any constraint on the state trajectory as given by the ProcStates function (except the compatibility mentioned above with D(P)), so as to allow hypothetical consideration of processes that may not be physically possible. Where necessary, extra assumptions can be made about a process under discussion, for instance that it is a physically realizable one (if its spatiotemporal origin is suitably positioned in a physically possible universe), or that the trajectory is continuous in space and time.

The definition embodies the current simplifying assumption that, at every given location within the home region, *all* aspects of state are included in the process. So the world “outside” the process is the world outside its home region, and incoming and outgoing dynamism (see Section 2.3) lies only between the process’s states and states outside the home region.

### 3.10. Meta-Dynamic Auto-Sensitivity of a Process

While this article has introduced meta-dynamic auto-sensitivity in the context of consciousness, this concept does not itself presume consciousness and may conceivably have broader application.

One could define many variant notions of meta-dynamic auto-sensitivity, depending on what sort of dynamism the process state at a given spacetime location is sensitive to, what locations/regions for dynamism are involved, and so forth. Candidates include sensitivity to current dynamism, to past dynamism regions in general, and to temporally abutting past regions. There is also the question of whether a past region should be required to have a home region that intersects the process’s home region or even be within it. For brevity I will confine attention to a variant that is the most convenient later in the case of conscious processes.

Since, in the current simplified treatment, a process covers all types of state and dynamism in its home region, any meta-dynamic effects of dynamism that lies within the home region can be considered to be effects of the process’s own dynamism (even if the dynamism involves some interactive dynamism with the world outside). Now, if we are to consider the process to be meta-dynamically sensitive to the dynamism of a recent sub-history (up to a current moment) taken as a unit, as we will need to for consciousness, it is reasonable to specify that the auto-sensitivity should be to past dynamism regions that temporally abut the current location in the process, and that also lie within the process’s home region. Furthermore, it is convenient to deploy the notion of intensity of meta-dynamic effect captured by $\kappa_{M,\mathrm{TH}}\left(Y,l\right)$, as this respects the holism of the effect of a past region *Y* of dynamism.

These points motivate the following definitions.

**Definition** **10.** *Given a process P and l∈Home(P), an inner abutting region of dynamism for l in P is a region of dynamism Y temporally abutting l where Home(Y)⊆Home(P) and Y∪{l} is connected.*

**Definition** **11.** *For any process P and l∈Home(P), the intensity of P’s inner abuttingly time-hopping meta-dynamic auto-sensitivity at l is the supremum of $\kappa_{M,\mathrm{TH}}\left(Y,l\right)$ over inner abutting regions of dynamism Y at l, or infinity if there is no such supremum. The intensity is notated as InAbutto_M,TH_(P,l).*

“InAbutto” is a convenient blend of “Inner”, “Auto” and “Abut”. The possibility of no-supremum is included for completeness, but may be legislated against in future developments.

The motivation here is to measure the maximal meta-dynamic effect that past regions have, and in particular to take care of the possibility that as we take smaller and smaller regions *Y* of the type mentioned in the definition, the intensity of meta-dynamic effect on state at *l* increases (possibly tending to a limit).

In fact, for most purposes below we only need the following, rather than any specific intensity:

**Definition** **12.** *A process P is inner-abuttingly time-hoppingly meta-dynamically auto-sensitive at l∈Home(P) if and only if InAbutto_M,TH_(P,l)>0 (i.e., if and only if there is some inner abutting region of dynamism Y for l in P such that $\kappa_{M,\mathrm{TH}}\left(Y,l\right)>0$).*

Notice that InAbutto_*M*,TH_(P,l) can depend on the particular *l*. This allows the value to tend to zero as *l* tends to a point on the border of the home region. So if the value is zero outside, there need be no discontinuity of the value at the border. (This is mentioned because the issue of discontinuities featured in Section 4.3).

### 3.11. Some Toy Systems with Time-Hopping Meta-Dynamism Arising

To make the ideas in our formalization more concrete, it is useful consider some simple toy systems where meta-dynamism dynamically arises. As the main thrust of the article is toward conscious processes, which are held (in Section 3.12) to involve inner-abuttingly time-hopping meta-dynamic auto-sensitivity, we will go through a sequence of illustrative toy systems that show some specific ways in which a process with certain forms of such auto-sensitivity might arise and cease. These processes are not claimed to be serious candidates for being conscious. In Section 3.15 and Section 3.16, we will draw from the toy systems here to present a further toy system that could be closer to being such a candidate.

The systems are specified merely by imagined, artificial equations of change crafted to illustrate various in-principle possibilities. We do not consider specific physical laws from which the equations would be derived, and we postpone to Section 4.1 the question of the nature of laws that could plausibly exist and that could lead to systems with flavours similar to our examples. We postpone to Section 4.3 a discussion of discontinuities of state with respect to space and time that arise in the systems.

Some particular features of the systems are as follows.

- They all make central use of region-valued expressions of the form $R_{\mathrm{loc}}\left[C\right]\left(l\right)$ as introduced above. It is important to bear in mind that the returned region *R* abuts *l* and that R∪{l} is connected.
- The valuation functions applied to dynamism regions will mostly be $\kappa_{\ldots }$ intensity functions as above. This is for the sake of concrete illustration, as these are the only specific valuation functions so far proposed in this article. However, the point of an example could potentially be preserved if a different sort of valuation function were used.
- Again for simplicity of presentation, all ordinary physical quantities will be assumed to be dimensionless, the systems will start at time 0, and we will not consider the world before time 0.
- The examples involve a main equation controlling the behaviour of an ordinary quantity *u*, by specifying the changing value of ∂u/∂t, often making it at least 1 and sometimes at least *u*. Thus *u* generally rises, sometimes exponentially. This is just for simplicity of presentation, and the illustrations are focussed on the nature of the meta-dynamism arising rather than the particular behaviour of *u*. Many other types of behaviour of *u* could be realized with variant equations, e.g., an oscillation of *u* within a range of small positive values.

An important lesson from the examples is that there are simple ways in which time-hopping meta-dynamism, including forms that provide auto-sensitivity to past dynamism, can start and stop. In addition, auto-individuation (to be discussed further later on) can arise in a natural way.

#### 3.11.1. Example 1—Starting of Meta-Dynamism

Here and in many of the other systems there are two ordinary quantities, *u* and *v*, plus time-derivatives.

The equations of change of the present system are:

(2) $$\frac{\partial u}{\partial t}\left(l\right)=\kappa_{B,v}\left(D\left(R_{\mathrm{loc}}\left[\lambda l^{\prime }.v\left(l^{\prime }\right)>0\right]\left(l\right)\right)\right)$$

(3) $$\frac{\partial v}{\partial t}\left(l\right)=1\;\mathrm{for}\;x\left(l\right)\in A\;\mathrm{and}\;\mathrm{any}\;t\left(l\right),\;\mathrm{otherwise}\;0$$

where *A* is some open, connected area of space. The initial conditions are that u=0 everywhere at time 0, v=−5 at time 0 within *A*, and v=0 elsewhere.

All quantities (e.g., *u*, *v* and dynamism intensities) will be constant across space at any given time, except that that their values in the spatial region *A* may differ from their values outside.

We use a particular number, −5, here for vividness, rather than a symbol constant such as $v_{0}$. Nothing hangs on the particular value except that it is negative.

The system’s behaviour is as follows.

- For locations *m* that are spatially within *A* (i.e., x(m)∈A), v(m) is negative when t(m)<5, zero when t(m)=5, and positive ever after. Outside *A*, v=0 always.
- So for all locations *m* with times up to and including 5 and any spatial position, $R_{\mathrm{loc}}\left[\lambda l^{\prime }.v\left(l^{\prime }\right)>0\right]\left(m\right)=\emptyset$. Thus the dynamism region delivered by *D* in Equation (2) is empty, and $\kappa_{B,v}$ delivers 0. Thus, ∂u/∂t=0 everywhere at times up to and including 5, and hence *u* remains at zero everywhere in that period.
- However, for *m* spatially in *A* and t=t(m)>5, that region-valued expression delivers the spacetime “cylinder” $R_{A,(5,t)}=\left\{l^{\prime }\;|\;x\left(l^{\prime }\right)\in A,t\left(l^{\prime }\right)\in \left(5,t\right)\right\}$. Note that this region plus *m* is connected, as required: *m* is one of the locations on the advancing face of the cylinder. We can notate the cylindrical region of dynamism delivered by *D* acting on this spacetime region as $D_{A,(5,t)}$. I assume $\kappa_{B,v}\left(D_{A,(5,t)}\right)>0$ since the dynamism in the region (continuously) constrains *v*.
- Hence, for *m* spatially in *A* and t(m)>5, ∂u/∂t(m)>0 and *u* always grows.
- For *m* spatially outside *A* and with any time value, there is no region abutting and connected to *m* in which v>0, so the region-valued expression delivers the empty set, so *D* delivers the empty dynamism region, $\kappa_{B,v}$ delivers 0, and ∂u/∂t and *u* are constant at zero.
- Consider the process *P* whose home region is the infinite cylinder $R_{5,\infty }=\left\{m\;|\;x\left(m\right)\in A,t\left(m\right)>5\right\}$. At each location *l* in this region, ∂v/∂t(l)=1,∂u/∂t(l)>0 and so v(l)=t(l),u(l)>0. Moreover, at each such location *l*, the ∂u/∂t aspect of ordinary state is—by virtue of the right-hand side of Equation (2) involving a non-empty dynamism region— time-hoppingly, meta-dynamically affected by that region, which is the dynamism cylinder up to t=t(l), i.e., $D_{A,(5,t)}$. That is, $\kappa_{M,\mathrm{TH}}\left(D_{A,(5,t)},l\right)>0$.

Since that cylinder’s home region $R_{A,(5,t)}$ is an inner abutting region for *l* in *P* (in fact, it covers the entire history of *P* before *t*) we have that *P* is *inner-abuttingly time-hoppingly meta-dynamically auto-sensitive at* *l*.

*P* at a given time has no sensitivity to any past region of dynamism that is not within its history up to that time. This makes *P*’s auto-sensitivity *auto-individuating*, according to a definition below.

As we do not yet have a theory of how dynamism intensity is defined, we cannot say much about the specific value of $\kappa_{B,v}\left(D_{A,(5,t)}\right)$ at a given *t* in our example. However, in future specification of how intensities work, it may well turn out that it does not depend on *t*. This is because, at the base level of dynamism, *v* is being constrained in a constant way. Then *u* would rise linearly during the unfolding of process *P*.

#### 3.11.2. Example 2—Ending of Meta-Dynamism

Unsurprisingly, just as time-hopping meta-dynamism can be “switched on” at a particular time (5) and place (*A*) as in Example 1, it can also be switched off (and on again, etc.). This is clearest in the following variant of Example 1, where the ∂u/∂t equation is as before but *v* oscillates, crossing zero:

(4) $$\frac{\partial u}{\partial t}\left(l\right)=\kappa_{B,v}\left(D\left(R_{\mathrm{loc}}\left[\lambda l^{\prime }.v\left(l^{\prime }\right)>0\right]\left(l\right)\right)\right)$$

(5) v(l)=2sinπt(l)−1forx(l)∈Aandanyt(l),otherwise0.

Now *v* is negative across *A* until time 1/6. After that point there is cylinder based on *A* growing in the time dimension, and it will be occupied by a process *P* that is inner-abuttingly time-hoppingly auto-sensitive (and auto-individuating) as before. However, as soon as *v* is negative again, when time (t(l)) has crossed 5/6, the region-valued expression reverts to delivering the empty spacetime region, because there is no region in which v>0 and that *abuts* *l*, until *v* again becomes positive later on, when time has crossed 13/6. Notice that in the cylinder portion $R_{A,(5/6,13/6)}$ it is also the case that there is no time-hopping meta-dynamism, because ∂u/∂t is not being affected by any dynamism region.

So, it is convenient for this example to consider a process *P* that temporally abuts 5/6. When time crosses 13/6, and *v* again rises above zero, a new, similarly auto-sensitive process $P^{\prime }$ starts. Notice that because $R_{\mathrm{loc}}$ delivers a region that is connected when *l* is added, $P^{\prime }$ is sensitive only to its own cylinder portion, not the previous one (that of *P*).

This definition of *P* and $P^{\prime }$ does not prevent us regarding the whole infinite cylinder as being occupied by a process that proceeds uninterruptedly from time 5, with *P* and $P^{\prime }$ and similar later processes as sub-processes, separated by sub-processes that contain no meta-dynamism.

#### 3.11.3. Example 3—A Reflexive Use of Meta-Dynamism

A interesting variant of Example 1 is where *v* rises linearly from −5 as in that example and the initial conditions are the same, but *u*’s changing is now described by

(6) $$\frac{\partial u}{\partial t}\left(l\right)=\kappa_{B}\left(D\left(R_{\mathrm{loc}}\left[\lambda l^{\prime }.v\left(l^{\prime }\right)>0\right]\left(l\right)\right)\right)$$

The only formal difference from Example 1 is that $\kappa_{B}$ is used instead of $\kappa_{B,v}$. A cylinder grows just as before, but now the valuation function’s result, while certainly still positive within the cylinder, is related not only on base-level dynamism involving *v* and ∂v/∂t but also to that involving *u* and ∂u/∂t themselves. Thus, there is a mutual dependence between *u*’s behaviour and the dynamism in the growing cylinder.

#### 3.11.4. Example 4—Diachronic Evaluation of Time-Hopping Meta-Dynamism

Consider

(7) $$\frac{\partial u}{\partial t}\left(l\right)=\kappa_{M,\mathrm{TH}}\left(D\left(R_{\mathrm{loc}}\left[\lambda l^{\prime }.v\left(l^{\prime }\right)>0\right]\left(l\right)\right)\right)$$

with *v* linear as before and with the same initial conditions. A crucial difference from Example 3 is that, at any time after 5, the time-hopping meta-dynamism, rather than the base-level dynamism, across the cylinder abutting that time is being evaluated. However, it is still the case that that cylinder of dynamism is affecting ∂u/∂t according to the equation, whatever the value returned by $\kappa_{M,\mathrm{TH}}$, even if zero. So there is still time-hopping meta-dynamism at the current location, irrespective of whether the abutting cylinder itself contains any time-hopping meta-dynamism or not.

But the abutting cylinder does in fact contain such meta-dynamism, because at every location within it the same comments hold. So there is in fact time-hopping meta-dynamism at every location in the abutting cylinder. So ∂u/∂t is positive at the current location. That location was arbitrary, so that derivative is positive everywhere in the cylinder.

Notice that at every location in the infinite cylinder, the time-hopping meta-dynamic effect of the abutting dynamism cylinder on current state is in part a matter of the time-hopping *meta*-dynamism in that cylinder having an effect. This affect can therefore be viewed as *meta-meta*-dynamic. This meta-meta-dynamism is then part of the dynamism that affects state at later times, meaning there is yet another layer of meta, and so forth. But the times being considered are any throughout the cylinder. So we could say there is, conceptually speaking, an infinite succession of meta-levels of dynamism throughout the cylinder. However, as I argue in Section 2.9, this is not an actual, vicious regress that should trouble us.

A point to note is that this succession of meta-levels exists for every time after 5, no matter how close to 5. So, despite complexity of the situation, we have an essentially constant state of affairs as regards dynamism throughout the cylinder. This makes it reasonable to suppose that the $\kappa_{\ldots }$ term delivers a constant value throughout the cylinder, so the equation for *u* reduces to ∂u/∂t=k for some positive constant *k*.

#### 3.11.5. Example 5—Combining Some Effects

This example varies Example 4, by combining it with Example 1 and also throwing in an ordinary term in the equation for ∂u/∂t.

(8) $$\frac{\partial u}{\partial t}\left(l\right)=u\left(l\right)+\kappa_{B,v}\left(D\left(R_{\mathrm{loc}}\left[\lambda l^{\prime }.v\left(l^{\prime }\right)>0\right]\left(l\right)\right)\right)+\kappa_{M,\mathrm{TH}}\left(D\left(R_{\mathrm{loc}}\left[\lambda l^{\prime }.v\left(l^{\prime }\right)>0\right]\left(l\right)\right)\right)$$

Now there is time-hopping meta-dynamism at every time during the infinite cylinder from time 5, by virtue both of the $\kappa_{B,v}$ term.and the $\kappa_{M,\mathrm{TH}}$ term. But note that the latter term evaluates the combination of the two types of time-hopping meta-dynamism within the abutting cylinder, even though the term only displays one of the two types that exist at the current location.

Because the $\kappa_{\ldots }$ terms are positive in the infinite cylinder, *u*’s derivative must be positive there too, and so *u* must be positive (having been zero on *A* at time 5). *u*’s value now provides an added boost to the value of the derivative.

#### 3.11.6. Example 6—A More Implicit Combination of Effects

Consider the following, based on Example 5.

(9) $$\frac{\partial u}{\partial t}\left(l\right)=u\left(l\right)+\kappa \left(D\left(R_{\mathrm{loc}}\left[\lambda l^{\prime }.v\left(l^{\prime }\right)>0\right]\left(l\right)\right)\right)$$

with linear *v* and initial conditions as before.

Since κ evaluates all types of dynamism, this works similarly to Equation (8) in the previous example, getting, qualitatively speaking, at least the effect of the $\kappa_{B,v}$ and $\kappa_{M,\mathrm{TH}}$ there. However, at any given location in the infinite cylinder, the κ term is positive irrespective of any consideration of meta-dynamism in the abutting cylinder. Aside from the potential difference this makes to how large the right-hand side of the equation is compared to Example 5, the considerations are much the same.

#### 3.11.7. Example 7—Synchronic Evaluation of Time-Hopping Meta-Dynamism

We now change Example 4 more radically, to use the following equation:

(10) $$\frac{\partial u}{\partial t}\left(l\right)=\kappa_{M,\mathrm{TH}}\left(D\left(R_{\mathrm{loc}}\left[\lambda l^{\prime }.v\left(l^{\prime }\right)>0\right]\left(l\right)\right),l\right)$$

with the same linear *v* and initial conditions.

∂u/∂t at *t* equals the intensity not of the time-hopping meta-dynamism in the abutting cylinder, but the intensity with which the dynamism (of any sort) in that cylinder is time-hoppingly meta-dynamically affecting (any aspect of) the state at *t*. However, that meta-dynamism is an aspect of the dynamism *at l itself*. So this is the first example in which dynamism at the current time is (potentially) explicitly constraining state at the very same time. The meta-dynamism can be thought of as the current, non-time-hoppingly meta-meta-dynamic affecting of current state by the (current) time-hoppingly meta-dynamic affecting (if any) of current state by past dynamism.

However, it is consistent to suppose that there is no time-hopping meta-dynamism in the system and that ∂u/∂t stays zero on *A* as well as outside. That zero-ness implies the $\kappa_{M,\mathrm{TH}}$ term is zero, consistent with there being no time-hopping meta-dynamism. I assume here that this solution is the one that is realized, because no equation displays any meta-dynamism that would justify the $\kappa_{M,\mathrm{TH}}$ term being positive. (However, further research is needed on this, as there could have been additional equations forcing ∂u/∂t to be positive, thus forcing the $\kappa_{M,\mathrm{TH}}$ term to be positive. There would thus be a requirement of time-hopping meta-dynamism with no constraint on what the meta-dynamism is other than that it have the intensity required by the equation.)

#### 3.11.8. Example 8—Dispensing with *v*

In the examples above, the condition to which $R_{\mathrm{loc}}$ is applied involves only *v*. This simplifies the systems conceptually and obviates some difficulties. But it is important to consider what happens when *u* is involved as well or instead.

We will just look at two possibilities here, both now omitting *v* and having the initial condition that *u* and its time derivative are zero everywhere. The first possibility is:

(11) $$\frac{\partial u}{\partial t}\left(l\right)=\kappa \left(D\left(R_{\mathrm{loc}}\left[\lambda l^{\prime }.u\left(l^{\prime }\right)>0\right]\left(l\right)\right)\right)$$

For simplicity, we specify that this equation applies only in spatial region *A*, with ∂u/∂t otherwise zero. Hence *u* is always zero outside *A*.

The difference from before is that we have a circular situation. In the infinite cylinder on *A* from time zero, a non-empty region temporally abutting a given location *l* is only returned by the region-valued expression if *u* has been positive somewhere up to now, but conversely, this can only have happened if the κ term has been positive so that *u* was able to rise from and stay above zero, and this can only have happened if earlier abutting regions had been non-empty. (An intensity function applied to an empty dynamism region delivers zero.)

We can break the cycle by appealing to the fact that ∂u/∂t and *u* are zero at the start of the infinite cylinder, at time 0. It is therefore consistent with evolutionary constraints that these quantities remain zero over *A* for some interval after 5. From this we can see that a consistent solution is for them to remain zero throughout the infinite cylinder.

Once again I assume that this is the solution that is realized, but we should note that a solution where *u* and its derivative are positive in the cylinder is conceptually more reasonable than in Example 7. This solution implies that a non-empty abutting cylinder is always returned by the region-valued expression. κ applied to the resulting dynamism cylinder is positive, partly because *u* is dynamically constrained within it. But also, because the dynamism cylinder is non-empty, the equation displays a particular meta-dynamic affecting of *u*’s derivative by that cylinder. This affecting is then a contribution to the dynamism evaluated by κ at later times. Thus, there is time-hopping meta-dynamism throughout the infinite cylinder, contributing to the value returned by κ and hence to the positive value of ∂u/∂t.

Of course, in a larger system, it would be possible for *u* to be forced to be positive by other influences, in which case we get a meta-dynamism effect that is displayed by (11).

However, the following case involves a simpler forcing of *u* to be positive in *A*:

(12) $$\frac{\partial u}{\partial t}\left(l\right)=1+\kappa \left(D\left(R_{\mathrm{loc}}\left[\lambda l^{\prime }.u\left(l^{\prime }\right)>0\right]\left(l\right)\right)\right)$$

again applying only in *A*, with ∂u/∂t=0 outside *A*, and with the same initial conditions. We obtain an interesting cylinder after time zero, because ∂u/∂t and hence *u* are guaranteed to be positive in *A*, irrespective of the value of the κ term. Thus, at every location in the infinite cylinder, there is the non-empty abutting cylinder forming its initial portion before the time of that location, so in particular there is time-hopping meta-dynamism at that location.

In this example, we have restricted the equations to apply only in *A* and have separately stipulated ∂u/∂t to be 0 outside *A*. Another way to get this effect would be to multiply their right-hand sides by a factor *w* as in the next example.

#### 3.11.9. Example 9—Gating by Ordinary Quantities

In the previous example, the question of whether there was meta-dynamism or not rested only on whether a region-valued expression delivered a non-empty region or not. But the presence of meta-dynamism can be separately controlled by ordinary quantities, as intimated when gating expressions were first described in Section 3.7. An elementary illustration is:

(13) $$\frac{\partial u}{\partial t}\left(l\right)=w(1+\kappa \left(D\left(R_{\mathrm{loc}}\left[\lambda l^{\prime }.u\left(l^{\prime }\right)>0\right]\left(l\right)\right)\right)$$

This equation applies everywhere, not just in *A*. Quantity *w* is an ordinary quantity that is always zero outside a spatial region *A* but is 1 across *A* at times after 5. *u* is everywhere zero at time zero. The equation therefore makes ∂u/∂t and hence *u* be always zero outside *A*.

For locations *l* spatially in *A* before time 5, ∂u/∂t is zero because *w* is zero, hence u=0. *w* being zero is enough to ensure there is no meta-dynamism at the location. But in any case, an empty region is returned by the region-valued expression.

However, in *A* after time 5, *w* is positive, so ∂u/∂t and hence *u* are positive, much as in Example 8. Hence also the dynamism region that is referred to is non-empty, the resulting positive value returned by κ pulls ∂u/∂t above 1, and that region (a cylinder from time 5) is meta-dynamically affecting ∂u/∂t. A contribution to the dynamism evaluated by κ is the similar time-hopping meta-dynamic effect at earlier times throughout that cylinder.

We could vary the example by using, say, $\kappa_{B}$ instead of κ. In this case, there would still be the time-hopping meta-dynamism at every location in the infinite cylinder, but it it would not contribute to the value delivered by $\kappa_{B}$.

In this example, we have assumed so far that *w* jumps discontinuously (within spatial region *A*) from constant zero to a constant non-zero value. It is useful to consider the more realistic possibility that *w*, while still always zero outside *A*, is always non-zero but tiny everywhere in *A*, except that it rapidly but continuously rises to a substantial value, say around 1, around time 5, and stays at such a value until rapidly but continuously falling back to being tiny but non-zero at from a later time, say 7. Because *w* is always non-zero in *A*, there is always, after time zero in spatial region *A*, a meta-dynamic effect on ∂u/∂t by a dynamism cylinder extending from time 0. However, assuming that the κ does not get large (let’s say it is always no more than 1), this meta-dynamic effect will itself be tiny, and ∂u/∂t will have a tiny positive value. However, soon after time 5, the effect becomes as substantial as in previous examples, relapsing to tininess at around time 7.

#### 3.11.10. Example 10: Non-Ordinary Region-Defining Conditions

In previous examples, the region of dynamism has always been defined by a condition on an ordinary quantity, e.g., the condition that *v* be positive everywhere in the region. But the condition does not need to be confined to ordinary quantities. Consider:

(14) $$\frac{\partial u}{\partial t}\left(l\right)=1+\kappa_{B,v}\left(D\left(R_{\mathrm{loc}}\left[\lambda l^{\prime }.v\left(l^{\prime }\right)>0\right]\left(l\right)\right)\right)+\delta_{d}\left(D\left(R_{\mathrm{loc}}\left[\lambda l^{\prime }.\kappa_{M,\mathrm{TH}}\left(l^{\prime }\right)>0\right]\left(l\right)\right)\right)$$

with *v* and initial conditions as in Example 1. The equation applies at all locations, not just in *A*.

The $\delta_{d}$ valuation function delivers 0 if its argument is the empty dynamical region and 1 otherwise. We use this function for simplicity here—other functions could be used, with more complex effects.

The second $R_{\mathrm{loc}}$ expression demands that the region be one in which at every location there be some time-hopping meta-dynamic effect on state at that location. Now, if it were not for the $\kappa_{B,v}$ term, we would have a circularity issue analogous to the one arising from Equation (11). But with that term, there is always a time-hopping meta-dynamic effect on state at locations *l* spatially within *A* after time 5, so there is time-hopping meta-dynamism throughout the infinite cylinder with base *A* from time 5. Thus, for *l* spatially in *A* the $\delta_{d}$ term will in turn involve a non-empty dynamism region (the abutting cylinder on *A* extending over times after 5) and will have value 1.

Notice that although the spacetime region in that term is selected by means of time-hopping meta-dynamism, it is the complete holistic dynamism occupying it that has a meta-dynamic effect on current state. So if, for instance, κ were used in place of $\delta_{d}$, it would return the full dynamism intensity of the region.

Also notice that that even with κ in place of $\delta_{d}$ in the last term of the equation, we should not assume that the intensity of time-hopping meta-dynamism in that region governs the intensity with which that region is meta-dynamically affecting current state via the term. Even though the region’s quantitative contribution to the right-hand side of the above equation may be small, the fact that it is affecting the vale of *u*’s derivative at all may mean that the meta-dynamic effect should be considered strong. On the other hand, if the term were multiplied by a factor *w* as in Example 9, then the smaller the value of that factor then the more that the κ term could vary while only having a small change of effect on the equation. (Of course, there is separate channel of meta-dynamic effect via the $\kappa_{B,v}$ term.)

#### 3.11.11. Example 11—Further Reflexive Involvement of Current Meta-Dynamism

Finally, only in Example above 7, which uses a $\kappa_{M,\mathrm{TH}}\left(Y,l\right)$ expression, do we have a case of *current* dynamism being explicitly referred to in an equation. However, this just a very special case of current dynamism being referred to. We only need look at a different, formally simple example of this, as in, say,

(15) $$\frac{\partial u}{\partial t}\left(l\right)=\kappa \left(D\left(l\right)\right)$$

For simplicity, as in Example 8, we restrict this to apply only inside a spatial region *A*. There can optionally be another ordinary quantity *v* linearly increasing in *A* as in Example 1 and others. Then any dynamism involving constraints on *u* or *v* is included in current dynamism, and is thereby implicitly referred to by Equation (15) in order to explicitly constrain ∂u/∂t. That explicit constraining is a meta-dynamic effect that is an aspect of the current dynamism. So at each location the current dynamism contains non-time-hopping meta-dynamism, and this is itself part of the dynamism that κ is evaluating and that is affecting *u*’s derivative.

Despite the conceptual complexity here, a solution for the behaviour of the system could be for ∂u/∂t to be constant over time in *A*. (Note that ∂u/∂t must always be positive, because there is always some base-level dynamism involving the ordinary quantities, whatever law-based constraining they might be subject to.) Now, the intensity of dynamism in any system is not necessarily affected by the mere fact that a given quantity *u* happens to be varying: the intensity may purely be a matter of *how* quantities are constrained, not directly of their rates of change. In our case, it would certainly seem consistent, that, however it is that κ is defined, there is a constant value *k* of κ(D(l)) that fits ∂u/∂t also being *k*.

Of course, there are more complex possibilities. If we include κ(D(l)) on the right-hand side of an equation such as the one in Example 2, giving a sequence of separated phases in which there is meta-dynamism, and assuming some such sequence still arises, then κ(D(l)) is presumably a quantity that has different values within those phases from its values between the phases.

### 3.12. Formulation of the PRAIS Assumption as a Necessity Condition

In Section 2.6, we stated a Necessity Claim in terms of PRAIS for consciousness. We now use the apparatus of Section 3.10 to formulate that claim in more specific terms involving time-hopping meta-dynamic auto-sensitivity as formalized in that section, thereby obtaining a “Formal Necessity Condition”. In Section 3.15 and Section 3.16, we will outline two toy systems exhibiting a process that satisfies the condition.

The reader should keep in mind that, on the one hand, the condition as stated is indeed only a *necessary* condition, so that, as far as it itself is concerned, further specifications and unrelated additional conditions may be needed for consciousness. On the other hand, we do also conjecture that some form of the type of meta-dynamism required by the Formal Necessity Condition is in fact, by itself, sufficient for consciousness.

The condition is based mainly on Definition 12 plus an implicit definition of what auto-individuation amounts to:

**Formal Necessary Condition for Consciousness:** There is a way *W* of being meta-dynamically sensitive to dynamism regions such that, for a process *P* to be (uninterruptedly) conscious, whether in a strictly on/off sense, or with degree greater than zero if consciousness is graded, it must be that:

- Home(P) is an open, connected region.
- *P* is *inner-abuttingly, time-hoppingly meta-dynamically auto-sensitive throughout*, where “throughout” means at every location in Home(P), and where the meta-dynamism is of type *W*;
- *auto-individuation*: for each l∈Home(P), and for each dynamism region *Y* that meta-dynamically affects state at *l* (i.e., $\kappa_{M,\mathrm{TH}}\left(Y,l\right)>0$ and the meta-dynamism is of type *W*), we have Home(Y)⊆Home(P).

### 3.13. Explanation of the Condition

The connectedness requirement on the process’s home region is imposed for the following reasons. First, it ensures that we are considering an uninterrupted experience. Secondly and more sweepingly, it seems to fit naturally with the idea of a unified experience: if, for instance, we were to allow the home region to consist of two spatially disjoint regions, it might be hard to justify that we are talking about the physical realization of a unified experience, unless we proposed physical interaction between them that was not considered part of the process, which would seem unmotivated and artificial.

However, this argument does not take account of the possibility of non-local spatial coordination based on entanglement, so may be relaxed in the future. Of particular interest is the Bohmian approach in [59] where the coordination in entanglement is based on the proposed quantum potential that has a holistic, spatially non-local guiding effect on all coordinated entities and that occupies space in which the entities are embedded. This potential or some variant of it could be considered part of the conscious process, providing connectedness of the home region once again. This point is continued in Section 4.1.4. (Bohm and Hiley [59] speculate about extension of their theory to handle consciousness, but on different lines from MDyn’s.)

The Necessity Condition demands the time-hopping version of meta-dynamism. Suppose we were to relax this and require only non-time-hopping sensitivity to own dynamism. This is clearest if we suppose there were no time-hopping meta-dynamism anywhere. This would still allow a process to be sensitive, at a given location, to its own local dynamism, and thus be sensitive to its own evolutionary consistency with surrounding states. But it would not be sensitive to the connectedness of the current state to any time-spanning (sub-)history as a unit. Hence, this article proposes that the time-hopping version of meta-dynamism is needed.

The auto-individuation clause in the Condition does achieve a form of auto-individuation as described in Section 2.5, because it means that, for any case of time-hopping meta-dynamism of type *W* affecting the process, the past region of dynamism *R* featured in that case cannot extend beyond *P*, so there is no possibility of type-*W* time-hopping meta-dynamic effect on *P* by a region that is even partially outside the process.

One may wonder whether the Condition is too demanding in a certain respect. This respect is that, at every time t∈Times(P), it requires meta-dynamic auto-sensitivity at *all* spatial places involved at *t* in the process. It might be thought that one could have a conscious process where at each time only some spatial places have sensitivity to the process history, but the remaining locations are nevertheless included in the process because later locations are sensitive to regions including them. This issue interacts with another significant question, as to whether we should allow the process to be sensitive to dynamism regions that are not entirely within the process. I leave these matters for future consideration.

### 3.14. The Way *W* and Centred Reflexivity

This article does not explicate what a “way” *W* might be like in the Necessity Condition, but one basic *possibility* is that it is vacuous and readable as “any way”. In that case the Condition demands that the process have non-zero inner-abuttingly time-hopping meta-dynamic auto-sensitivity (with no further restrictions) throughout, but no time-hopping meta-dynamic sensitivity to dynamism regions not contained within itself. A more refined version, to be investigated in future work, would arise if the process only involved certain aspects of ordinary state and concomitant aspects of dynamism. Then auto-individuation would be achieved by the process having sensitivity to that sort of dynamism inside its home region but not to that sort outside its home region. Furthermore, in the future it will be desirable to investigate the possibility that there is some further general condition restricting the type of meta-dynamism.

A important constraint on the way is suggested by examination of the toy examples in Section 3.11. In most cases in those Examples, we had the location-wise condition C used in a region-valued expression $R_{\mathrm{loc}}[\left(C\right]\left(l\right)$ in an equation depend on an ordinary quantity *v* different from the quantity ∂u/∂t that that equation was primarily constraining. (“Primarily”, because that is what our focus was on, and *v* has a particular behaviour forced by other equations. However, typically, the quantities in an equation are in mutual constraint with each other.)

Furthermore, we often restricted explicit dependence on past base-level dynamism to be that involving *v*, by virtue of of using $\kappa_{B,v}$, although that dynamism does involve *v*’s constraints with respect to other quantities such as *u*. As a result, meta-dynamic sensitivity to past base-level dynamism is in a sense *v*-focussed. But then, recursively, even meta-dynamic sensitivity to past meta-dynamism becomes indirectly *v*-focussed. This is in conceptual tension with the fact that our primary concern is with the behaviour of *u* and in particular with time-hopping meta-dynamic effects on it (via ∂u/∂t).

By contrast, in Examples 8 and 9 (Section 3.11.8 and Section 3.11.9), we based C on *u* itself. Thus the meta-dynamism that arises is much more intimately and centrally based on the constraining of *u*. We can see a more intense and direct reflexivity in the way that *u*’s trajectory and the overall dynamism constrain each other.

It is unclear as yet how to translate this into a precise addition to the Formal Necessity Condition, but the general suggestion is that way *W* should involve a requirement that the auto-sensitivity in the Condition should be a matter of certain aspects of the process state at any time being affected through time-hopping meta-dynamism that *is centrally and directly based on those very aspects*. For convenience I call this the *Centred Reflexivity* requirement.

### 3.15. A Toy System Satisfying the Formal Necessity Condition

Several of the toy systems in Section 3.11 already obey the Necessity Condition (although mostly only if we ignore Centred Reflexivity mooted in Section 3.14), but here we build on those examples with new considerations. We consider the following system and show that it obeys the Condition plus Centred Reflexivity. As with the toy systems in Section 3.11, we use $\kappa_{\cdots }$ measures for the sake of illustration, but other dynamism valuation functions could potentially lead to satisfaction of the Condition; and the use of a simple equation for ∂u/∂t, leading *u* to keep on increasing, possibly to large values, is again just for simplicity of illustration. Many other types of behaviour could be realized, including where *u* keeps to small positive values during the process of interest.

(16) $$\frac{\partial u}{\partial t}\left(l\right)=w\left(1+v.\kappa_{B,u}\left(D\left(R_{\mathrm{loc}}\left[\lambda l^{\prime }.u\left(l^{\prime }\right)>0\right]\left(l\right)\right)\right)+\kappa_{M,\mathrm{TH}}\left(D\left(R_{\mathrm{loc}}\left[\lambda l^{\prime }.\kappa_{M,\mathrm{TH}}\left(l^{\prime }\right)>0\right]\left(l\right)\right)\right)\right)$$

Ordinary quantity v(l) (or an expression v(l) built only from ordinary quantities) is zero at all *l* except in a spatial region *A* over time interval (5,6), where it is 1, and after time 6, when it has a small positive value. The above equation applies everywhere, not just within *A*. Ordinary quantity or expression w(l) is zero at all *l* except that it is 1 in spatial region *A* over time interval (3, 300). (The stepping behaviour of *v* and *w* is for simplicity of presentation: temporally continuous versions could be used instead.) At time zero, u=0 and ∂u/∂t=0. I assume that dynamism-intensity functions deliver values in [0,1].

The form of this equation is roughly similar to Equation (14) for Example 10 (Section 3.11.10). One important change is that *u* itself replaces *v* in the first κ’s subscripts and in the condition in the first region-valued expression. The switch from *v* to *u* is done to conform to the requirement of Centred Reflexivity. Another important change is the introduction of the *v* and *w* factors to provide gating of meta-dynamism by ordinary values, much as in the use of *w* in Equation (13) in Section 3.11.9.

The behaviour of the system is as follows. ∂u/∂t and hence *u* is always zero outside *A*, because *w* is zero there. Furthermore, *w* being zero outside *A* means that there is no meta-dynamism outside *A*.

∂u/∂t is positive in *A* between times 3 and 300 because *w* is positive in *A* then, and is otherwise zero in *A*. Notice that this means that *u* is always positive in *A* after time 3.

So, in *A* after time 5, *v* and *u* are always positive, and as long as *w* stays non-zero, there is a meta-dynamic effect displayed by the $\kappa_{B,v}$ term from $D_{A,(5,t(l))}$, the dynamism cylinder, with base *A*, from time 5 that temporally abuts the current location *l*.

But because there is therefore this first channel of meta-dynamism in *A* at all times in (5,300), the region-valued expression in the $\kappa_{M,\mathrm{TH}}$ term is also non-empty, and returns the spatiotemporal cylinder $R_{A,(5,t(l))}$. (Recall here that there is no meta-dynamism at all outside *A*, so the condition in the region-valued expression is only satisfied by locations $l^{\prime }$ spatially within *A*.) Thus, the dynamism region occupying that cylinder, $D_{A,(5,t(l))}$ again, has a second meta-dynamic channel of effect on current state as long as *w* stays non-zero. Because of the application of $\kappa_{M,\mathrm{TH}}$ in the equation to that dynamism cylinder, the time-hoppingly meta-dynamic intensity of that cylinder as a whole is what contributes to the equation via that channel.

In time interval (3,5], *v* is zero, so the first channel of meta-dynamism does not yet exist. Thus, the $\kappa_{M,\mathrm{TH}}$ term gives rise to a circularity in the system much like the circularity in Example 10 (Section 3.11.10). I assume this means that no meta-dynamism arises via this term, i.e., the second channel of time-hopping meta-dynamism does not yet exist either.

Both channels of meta-dynamism disappear when time crosses 300 and *w* becomes zero again.

We therefore, much as in the previous toy Examples, get a process *P* that is inner-abuttingly time-hoppingly meta-dynamically auto-sensitive throughout, with home region $R_{A,(5,300)}$. That region is open and connected, satisfying there first requirement of the Formal Necessity Condition. The auto-sensitivity satisfies the second requirement, as amplified by the Centred Reflexivity condition. Thirdly, the auto-sensitivity is auto-individuating because the process has no meta-dynamic sensitivity to any dynamism outside its home region (so it does not matter what “way” of being meta-dynamically sensitive to dynamism regions turns out to be required in the Formal Necessity Condition).

The system is a special one in that at each location the process is auto-sensitive to the whole of itself up to the time of that location. In more elaborate systems, the process could be sensitive to a history that is shorter and does not go up to the spatial borders of the process. A shortened history arises in the next subsection.

#### Impure Auto-Sustenance of the Meta-Dynamism

Now we attend to the reduction of the value of *v* when time crosses 6. This is not included for satisfying the Necessity Condition, but rather because it illustrates what we can call *[partial or impure] auto-sustenance* of the time-hopping meta-dynamism. Even though *v* becomes small, from which it is reasonable to assume that the first channel of meta-dynamic effect from the abutting cylinder now has low intensity, the fact that *any* time-hopping meta-dynamism continues to exist after time 6 is enough to ensure that the second channel is present (the channel via the second $\kappa_{\ldots }$ term), while *w* remains non-zero. The intensity of that second channel is substantial. Furthermore, the second channel, as it contributes to the equation the time-hoppingly meta-dynamic intensity of the abutting cylinder, is more and more an effect from a dynamism region whose own internal meta-dynamism is dominated by the second channel as opposed to the first channel. That is, as time progresses the meta-dynamism is more and more indirectly connected to the base-level dynamism on which the meta-dynamism is originally founded. In a sense, the meta-dynamic auto-sensitivity gets purer and purer: more and more a sensitivity to itself, and its continuing intensity of effect is more and more a matter of its presence up to the current moment.

As for the behaviour of *u* itself, recall that the system is wide open to be varied to give other types of behaviour than an always-rising one. For instance, *u* could keep within range of very low positive values, so that the main activity lies in the meta-dynamism itself.

Having said this, the auto-sustenance is impure because the involvement of the first channel of meta-dynamism continues to be a necessary ingredient in keeping the meta-dynamism going.

Factors *v* and *w* might be based on the type of quantity to be discussed in Section 4.1, such as a measure of system complexity. So, in the case of *v*, such quantities could be important for the triggering of consciousness and to some extent its sustenance. *w* in our system is not a trigger but rather a factor included to limit the time-span of the consciousness process, and might be based on something like the general level of arousal of the organism, as reflected by local side-effects such as neural firing frequencies or chemical concentrations.

### 3.16. A Purified and Historically Limited Toy System

The impurity mentioned in the previous subsection can be reduced, in particular to eliminate the direct involvement of the time interval (5,6) in the first channel of meta-dynamism. The idea is to limit the distance that the time-hopping goes back in time, rather than always letting it go back to 5. In principle this would arise if there were a time limitation in the conditions used in the region valued expressions, e.g., as in

$$\lambda l^{\prime }.\left(\kappa_{M,\mathrm{TH}}\left(l^{\prime }\right)>0\;\mathrm{AND}\;t\left(l\right)-t\left(l^{\prime }\right)<\tau \right)$$

for some tolerance τ, which could be either constant or variable. If this were used in Equation (16), especially with τ relatively small compared to the interval (6,300), then as time goes on the time-hopping meta-dynamism becomes more distantly related to the base-level dynamism in (5,6).

Such a time-window sub-condition might conceivably arise by specification of some more complex and general sub-condition in a law, and therefore be an acceptable feature of our toy system. But it is beneficial to discern possibilities that are less arbitrary, with a view to approaching more closely what might appear in a law. For instance, the above temporal sub-condition might be replaced by a condition on the lines of those to be suggested in Section 4.1, such as behavioural complexity or structural features, or on some local physical feature such as neural activity oscillation frequency. If such a condition is only satisfied in short bursts it would limit the extent of time-hopping.

As will be further discussed in Section 5.2, the discussion of purity here is relevant to the notion of almost-pure consciousness, if some type of auto-sensitivity fitting the Formal Necessity Condition is also sufficient for consciousness.

## 4. Formal Treatment: Additional Considerations

### 4.1. What Gating Expressions are Reasonable in Laws?

Equations of the change of specific systems, such as the toy systems in Section 3.11 and Section 3.15, should be derivable from general-purpose, fundamental physical laws. The main issue that we need to address is the gating expressions in the equations of change. These must derive somehow from gating expressions in laws. So I will suggest types of gating expressions that could be general-purpose enough to reasonably exist in laws and lead to gating expressions in equations of change with effects like those in the Examples. (Despite the suggestions to be made, it is beyond the scope of this article to propose a particular set of modified physical laws that would lead to the above toy systems.)

I will approach the issue by continuing to assume that dynamism regions are specified by means of region-valued expressions based on region-delivering functions such as $R_{\mathrm{loc}}$. Hence, a main task is to consider what sorts of location-wise conditions C such a function could reasonably be applied to in laws, and similarly to consider reasonable sorts of whole-region condition H could be used in region-valued functions like $R_{\mathrm{reg}}\left[H\right]$ (see Section 3.4).

A related concern is with factors such as the ordinary quantities or expressions *v* and *w* in the system of 3.15 that help provide gating of dynamism. Comments below about use of ordinary quantities in conditions C or H carry over to such factors.

One guiding consideration for the task is that the article’s approach is focussed ultimately on the meta-dynamism that MDyn says is needed for consciousness, but recognizes that it *may* be that such meta-dynamism arises only in complex physical structures across a substantial region of space (such as a certain type of neural or similar network, comparable to the size of at least a small animal’s brain). In order to service this possibility, if location-wise consciousness-relevant conditions *C* are proposed, they had better be conditions that can indeed be evaluated at a single location within a complex structure while also being suitably affected by the locations’ being within that overall structure. On the other hand, a holistic condition *H* must be such as to measure the appropriate things about the complex structure.

At the same time, it should be remembered that it is possible that consciousness is *not* dependent on a complex structure, and that anyway there may be meta-dynamism that has no connection to consciousness and that arises much more simply.

Another consideration is that, while meta-dynamism in general may conceivably be fairly ubiquitous at least at tiny intensities, the meta-dynamism needed for consciousness may need be sparse, only arising in special circumstances, in which case consciousness-relevant conditions C (location-wise) and H (whole-region) in region-valued expressions may need to be only sparsely satisfied (although we should bear in mind that such conditions are not in sole charge of gating).

I do not specifically propose that, in realistic laws, simple location-wise conditions such as $\lambda l^{\prime }.u\left(l^{\prime }\right)>0$, where *u* is an ordinary physical quantity such as an electric field potential, are used to define the dynamism regions referred to in gating expressions in laws. Such conditions mainly served a simplifying, presentational purpose in our examples. But we should not entirely discount the possibly of such conditions appearing in laws. For instance, conditions might test for unusually high levels of excitation of certain particles (whether fundamental or complex ones such as atoms) or presence of a special sort of particle. It could even be that some such matter is a local side-effect of the presence of a certain type of physical structure spanning an appreciable region, so that specialized types of meta-dynamism could arise as indirect result of that structure being present.

Furthermore, a condition C—in a region-valued expression of a form such as $R_{\mathrm{loc}}[\left(C\right]\left(l\right)$—that could reasonably be used in laws might nevertheless, in the context of a particular physical system, reduce to, or be equivalent to, some relatively simple condition on one or more ordinary quantities that are not specifically mentioned in the law, a condition that would typically not be appropriate to some other distinctly different type of system.

I propose here four broad possibilities for what a reasonable C and/or H might test for, in the following sub-subsections.

But first, we should briefly consider what valuation functions might be used other than the dynamism intensity functions and $\delta_{d}$ function used above. A detailed answer waits upon future clarification of the intrinsic nature of dynamism. However, dynamism is intuitively a highly structured aspect of the world, with many interacting sub-aspects. We can therefore envisage valuation functions that are based on extracting aspects of structure in a more refined way than implied by the various intensity functions above, or that respond to global properties of the structure. A handle on this stands to be provided by the close link between dynamism and the web of synchronic and diachronic constraint amongst aspects of world state—see Section 4.2 and Section 5.7. Valuation functions could perform tasks that are related to examining and selecting from the geography of this web.

#### 4.1.1. Testing Intensities or Other Valuations or Features of Dynamism

There is one type of C that is much used in the toy Examples above and that is ready to be transposed to laws. For instance, in Equation (14) in Example 10 we used $\lambda l^{\prime }.\kappa_{M,\mathrm{TH}}\left(l^{\prime }\right)>0$. This condition is clearly not tailor-made to any very specific sort of system, and there appears to be no other reason why it should not be used in a law. To generalize, any dynamism intensity function applicable to a single location could similarly be used, with the possible exception of intensity functions like $\kappa_{B,v}$ that are restricted to some particular ordinary quantity *v*. And of course, the threshold need not be 0, or indeed be any specific constant value, rather than being specified by some variable expression (that in particular could itself involve dynamism intensities). For instance, if $\kappa_{B}$ were used, then a sufficiently high location-wise intensity of base-level dynamism throughout a region could lead to that region then having a meta-dynamic effect on something. I have no specific reason for proposing that some law have such an effect, but it is a general enough type of condition to appear in a fundamental law.

Generalizing further, other location-wise dynamism valuation functions β that are investigated in future research could potentially be applied, in a condition such as $\lambda l^{\prime }.\beta \left(D\left(l^{\prime }\right)\right)>\theta$ for some threshold θ, or in a condition that tested various valuation-function values against each other.

Moreover, conditions need not be quantitative at all. Given that the dynamism at a location is a complex structured entity of some sort, there may be many useful functions that test for the presence or absence of particular qualitative features of the structure, features that are not tailor-made for any specific physical system.

Equally a global condition H as above could involve holistic dynamism intensities, any other valuation functions applicable to dynamism regions, or qualitative features of a dynamism region.

#### 4.1.2. Testing Complexity

Conceivably, there could be some measure of the overall degree of complexity of interaction over a spacetime region, or a test for the presence of a particular qualitative type of complexity.

One illustrative possibility on these lines could be for a holistic condition H to involve a measure of the integratedness of information on the lines of the measure Φ in the Integrated Information Theory of consciousness [7,60]. The exact measure used in the theory may well not suitable, as some authors have pointed out features that make it non-objective [61], i.e., dependent on exactly how an analyst views a given system, whereas we require a completely objective measure. Another problem is that the measure as it stands rests on probabilistic patterns of cause and effect, and it is not clear what mechanism could reasonably be proposed that would observe the necessary probabilities, especially of this mechanism is to be enshrined in fundamental physical laws. While the theory involves mathematical equations that derive values for the measure from such patterns, and humans can consciously apply the equations, thereby demonstrating that there do exist naturally arising systems can in principle derive the values, this is far from being able to propose that underlying the meta-dynamism that MDyn says is needed for consciousness is a process that examines the probability patterns.

However, the next subsection exploits an IIT-related possibility suggested by others.

#### 4.1.3. Testing Quantum Integrated Information

To anticipate a discussion in Section 5.4.2, Kremnizer and Ranchin [62] have proposed a measure of Quantum Integrated Information (QII), for which the authors give a precise physical formula. QII is not intended to produce the values that IIT’s measure does, but instead is inspired by similar intuitions. In particular, it is a completely objective quantity defined at a fundamental physical level, and of complete generality. Thus, potentially, QII could be used within a C or H condition, as well as or instead of its originally proposed use in controlling quantum state collapse. Naturally, the suggestion is not tied to the specifics of QII. Analogous measures could equally be considered.

A nuance here is the question of whether QII is responsive only to ordinary physical quantities or also to dynamism, including possibly meta-dynamism. I return to this in Section 5.4.2.

#### 4.1.4. Testing Bohmian Quantum Potentials

The approach of Bohm and Hiley [59] to quantum theory is of interest for future quantum-theoretic development of MDyn. According to the approach, in any quantum system there is a “quantum potential” (typically notated as *Q*) defined over the spacetime region of the system and that intricately guides the (say) particle movements of the system. For instance, in a two-slit experiment, each given particle is guided by *Q* to take a particular, defined trajectory, with particles starting off in slightly different conditions getting different such trajectories. Bohm and Hiley emphasize a quite elementarily derived property of *Q* is that its strength is independent of the distance from the system components (e.g., slits and particles) that give rise to it, and there can be rapid, intricate spatial/temporal variations of the potential at a given location, giving rise to complex effects on (e.g.,) particles, and reflecting the nature and layout of distant parts of the system.

The *Q* over the spacetime region of a given system therefore stands as a promising candidate for an objective, universally arising, physical quantity that reflects at a given location something about the overall structure of the system. It may therefore have the sort of properties needed for conditions C that give rise to meta-dynamism in restricted circumstances that are dependent on the presence of certain sorts of system structure.

### 4.2. Addressing the Core Reflexivity of Dynamism

Here we make moves towards formally expressing the intensely reflexive operation of dynamism in the presence of meta-dynamic laws.

Consider the application of laws to location *l*, and consider TotalState(l). The laws state (i) mathematical constraints between aspects of TotalState(l) and other such aspects, and (when there is a time-hopping meta-dynamic law) between aspects of TotalState(l) and dynamism in past regions of dynamism. There are also (ii) the mathematical evolutionary-consistency constraints between state quantity values at *l* and related values at arbitrarily close times (and places), based on the use of derivatives in laws. Finally, there is (iii) the constraint that the dynamism at a location should by itself logically determine the overall constraining of all quantities directly relevant to that time, including of itself.

We can thus identify an overall *web of constraint* at *l*, where each node in the web identifies a quantity and its value, and a link or hyperlink (a link that joins more than two nodes together) identifies a constraint at *l*. The constraining of dynamism by itself is the crux of the reflexivity in this subsection, whose task is mainly to clarify what the reflexivity amounts to.

I assume that there is a function **MathWeb** that, when applied to TotalState(l) and some suitable portion *h* of the world’s history (see below) of states that led evolutionarily up to TotalState(l), delivers the web of constraint consisting of constraints of types (i)–(iii) above. The name of this function reflects its purely mathematical nature. Given some specific laws, the web is purely a matter of their mathematical form, the nature of the above derivatives, and the equation involving a function called DynWeb below, i.e., (19). Note that since TotalState(l) is an actual total-state arising in the universe, the constraints in the web are all in fact satisfied. If on the other hand we were to consider a hypothetical total-state, it might be unsatisfied.

The nodes of the web are (at least) a node for each ordinary state quantity (including derivatives) at the location *l*, the dynamism at *l*, and the regions of dynamism referred to by law applications at *l*, plus all similar quantities for all locations in some history as mentioned above. Thus, there are uncountably many nodes. Furthermore, as we are about to see, the web is reflexive in needing to include itself as a node. (Note that, whereas dynamism is reified, it is not assumed that constraint webs are physically real. They are just a part of our analysis, as far as the present article is concerned. However, see an alternative view in Section 5.7.)

Constraint (iii) implies a function **DynWeb** from the dynamism at a given location to the constraint web there. Clearly, at any location the webs delivered by MathWeb and DynWeb must be the same. Furthermore, the dynamism at *l* is part of the overall state at *l*: it is $\mathrm{TotalState}{\left(l\right)}_{d}$. So, we have, for some suitable history, *h*, at *l* (i.e., *h* is the collection of states at the locations in an open temporally abutting spacetime region, where presumably also the region plus *l* is connected),

(17) $$\mathrm{MathWeb}\left(h,\mathrm{TotalState}\left(l\right)\right)=\mathrm{DynWeb}\left(\mathrm{TotalState}{\left(l\right)}_{d}\right)$$

This equation is just an expression of constraint (iii), which is itself a part of the web. So we now seeing more precisely what the intense reflexivity of dynamism amounts to. It is the fact that *the constraint web contains a constraint between that very web and the dynamism at l*. The web is also thus a node within itself, and equally the dynamism is a node in the web. The dynamism is, in general, also constrained in other ways with respect to other nodes in the web.

So Equation (17) captures (at least some of) the reflexivity of dynamism, even without any time-hopping. The web is in part reflexively defined, in being a function of (amongst other things) itself. Indirectly therefore, the dynamism is in part reflexively defined. We can more fully see the role of dynamism in the web by expanding the total-state into its ordinary and dynamic components:

(18) $$\mathrm{MathWeb}\left(h,\left(\mathrm{TotalState}{\left(l\right)}_{o},\mathrm{TotalState}{\left(l\right)}_{d}\right)\right)=\mathrm{DynWeb}\left(\mathrm{TotalState}{\left(l\right)}_{d}\right)$$

This suggests that the dynamism should be linked not only by a DynWeb-based constraint to the whole web, but also by a DynWeb-and-MathWeb-based constraint to itself. The latter takes the form of a hyperlink that brings in all ordinary quantities.

As to the nature of *h*, note first that, were it not for time-hopping aspects of laws as instantiated at *l*, it could be any partial history of the world from some time arbitrarily close to the time of *l* that was adequate to determine the total state at *l* (recalling the observations in Section 3.2). Furthermore, we can allow all aspects of state at all locations with times from the start of the history up to but not including t(l) to feature in *h*. (Or, we could exclude certain spacetime locations, e.g., those outside the past light-cone of *l*, depending on what assumptions hold in the overall physics).

However, when there is time-hopping meta-dynamism at *l*, *h* must go back far enough to encompass all past state that directly or indirectly affects state at *l* via time-hopping. So, as a minimum, *h* contains all locations in all past dynamism regions meta-dynamically affecting state at *l*. Moreover, further, if at some location *m* featuring in *h* the dynamism has time-hopping aspects, then *h* must go back far enough to encompass *that* time-hopping, as the past dynamism that it brings in is part of what determines the evolution of state within the history. It is not enough just to consider any time-hopping in the dynamism at *l* itself: all chains of hopping going back into the past from *l* must be encompassed. With this in mind, we have the following definition, catering for such chains.

**Definition** **13.** *A region R of spacetime is backwards hop-closed if and only if, for all locations m in R, the time-spans Times(Y) of the regions Y of dynamism involved in time-hopping aspects of D(m) are all within the time-span of R.*

It could in principle be that, for *R* to contain some particular location *m*, the chaining of time-hopping goes back to the start of the universe or infinitely far back if the universe has no start. This does not affect the treatment below, but I expect that, in practice, chains of time-hopping only go finitely far back, or we can apply a cut-off at the price of only insignificantly small defects of accuracy in how states are determined by their histories.

So, we require the history *h* above to be the conglomeration of states over some spacetime region that: temporally abuts *l*; is such that when *l* is added we get a backwards hop-closed region; and contains enough locations to determine TotalState(l). To emphasize the possible dependence that the extent of *h* has on time-hopping in the dynamism at *l*, I notate the history as $h{\left(l,\mathrm{TotalState}\left(l\right)\right)}_{d})$, although generally there is no unique history that we need to choose. Introducing this into Equation (18), we get

(19) $$\mathrm{MathWeb}\left(h{\left(l,\mathrm{TotalState}\left(l\right)\right)}_{d}\right),\left(\mathrm{TotalState}{\left(l\right)}_{o},\mathrm{TotalState}{\left(l\right)}_{d}\right))=\mathrm{DynWeb}\left(\mathrm{TotalState}{\left(l\right)}_{d}\right)$$

However, with no time-hopping, the backwards extent of the history can be limited arbitrarily, and concomitantly, the web can be limited to times arbitrarily close to t(l).

There is a further consideration. It may be that for a given web of constraint at a location *l*, there is only one value of dynamism that implies that web. In that case, the DynWeb function has an inverse ${\mathrm{DynWeb}}^{-1}$, which is at least a partial function, defined when the web is a physically possible one. For convenience, we can re-notate ${\mathrm{DynWeb}}^{-1}$ as WebDyn. Then:

(20) $$\mathrm{WebDyn}\left(\mathrm{MathWeb}\left(h{\left(l,\mathrm{TotalState}\left(l\right)\right)}_{d}\right),\left(\mathrm{TotalState}{\left(l\right)}_{o},\mathrm{TotalState}{\left(l\right)}_{d}\right)\right){)=\mathrm{TotalState}\left(l\right)}_{d}$$

This conveys a clearer sense in which the dynamism (and hence also the web of constraint) at *l* is in part reflexively defined.

We now check that the treatment is reasonable when we consider Scenarios 0 and 1 in Section 3.2 and Section 3.3, where there was no meta-dynamism. After that we will analyse what happens when there is meta-dynamism, in further Scenarios 2 and 3. For these purposes we go back to Equation (19).

We can deal with Scenario 0, with dynamism not reified and all laws ordinary by removing dynamism from total-states and removing dynamism and all constraints involving it from the web. To fit with the above treatment we can stipulate that $\mathrm{TotalState}{\left(l\right)}_{d})$ is undefined, that the value delivered by MathWeb is just the web formed from constraints of types (i) and (ii) above, confined to ordinary aspects of state, and refrain from postulating a function DynWeb, so that Equation (19) is inapplicable. The history used to define the web can be made arbitrarily brief. Our treatment simply reduces to the formalization of the (i) and (ii) constraints (with no involvement of time-hopping) as a satisfied web covering an arbitrarily brief history. This is just a regimentation of the view set out in Section 3.2 of how ordinary laws work in a universe without reified dynamism.

There is also an alternative approach to Scenario 0 that drops out of the following approach to Scenario 1.

#### 4.2.1. Scenario 1: Reified Dynamism but No Meta-Dynamism

Scenario 1 includes reified dynamism but no meta-dynamic laws, so there is no constraint of types (i) and (ii) between dynamism and anything else. Dynamism is only constrained by Equation (19), expressing constraint (iii). So, the physical reification of dynamism does not affect the way the world evolves.

Hence, it is clearly consistent to take MathWeb to deliver exactly what it does in Scenario 0, except for the addition of $\mathrm{TotalState}{\left(l\right)}_{d})$ as a node and of that constraint between it and the whole web. This is simply tantamount to a definition of what the dynamism at a location is in terms of the remainder of the web: the value of the dynamism does not matter except in so far as it is related to the web by that constraint. This is what we want for the Scenario, namely that, when no law is meta-dynamic, our proposal reduces to a standard form of physics insofar as the evolution of ordinary state is concerned. Reifying the dynamism should make no difference to how ordinary state evolves if dynamism does not appear in laws.

We can modify this approach slightly to provide the promised variant approach to Scenario 0. The modification is to regard dynamism as existing purely metaphysically and not physically. We then merely use $\mathrm{TotalState}{\left(l\right)}_{d})$ as a convenient formal denotation of that metaphysical dynamism. MathWeb merely delivers a theoretical construct anyway, not a physically real entity, so we are free to include purely metaphysical dynamism within it. We therefore get an approach to Scenario 0 where we have chosen to add metaphysically existing dynamism and where DynWeb links that dynamism to the web.

#### 4.2.2. Scenario 2: Including Non-Time-Hopping Meta-Dynamism

In this scenario there are some laws that refer to dynamism, but only via D(l) where *l* is the current location. So there is meta-dynamism (I assume this is not suppressed by zero-valued factors like the *w* in an illustration in Section 3.11.9). But the meta-dynamism is all non-time-hopping. Meta-dynamism is *displayed* through the stated synchronic constraints between (aspects of) D(l) (via valuation functions) and other quantities at *l*, and possibly between different aspects of that dynamism. But any current meta-dynamism is also within D(l), the current punctate dynamism, and so can also be *referred to* indirectly by use of D(l), and separately picked out by valuation functions.

We still have a constraint web over an arbitrarily brief history, as in Scenario 1, but now the node for the punctate dynamism at *l* is linked by constraints to nodes for quantities in the web other than the whole web (including possibly nodes for the dynamism at prior locations). So dynamism, the web and the quantities in the web are cooperatively defined, with the partial reflexivity of the web and of dynamism having real significance: we can no longer regard the ordinary-state aspect of the web as independently defined, with the dynamism a function of it.

#### 4.2.3. Scenario 3: Including Time-Hopping Meta-Dynamism

This is the last of our succession of Scenarios. We now have laws that are meta-dynamic by referring to past non-empty regions of dynamism (and laws may still refer to current dynamism as in Scenario 2). Thus, there is time-hopping meta-dynamism. Again, I assume it is not blanked out by zero factors at the location *l* we are considering.

The web of constraint for Scenario 3 it is like that in Scenario 2 except that now it extends over a hop-closed history and contains nodes for the past regions of dynamism implied by time-hopping aspects of laws. These nodes are connected by law-based constraints to nodes for aspects of state at later locations in the history or at *l*. The constraints are the ones displayed by the laws that mention past regions of dynamism.

Note that, unless we want to contemplate the possibility of the past being changed, such regions of dynamism that are past relative to *l* are not subject to change for the purpose of defining and satisfying the web of constraint at *l*, any more than past values could be changed in Scenarios 1 and 2.

In the Scenario, it is still possible for a law to mention current dynamism using D(l). But that dynamism will now itself include time-hopping meta-dynamism affecting state at the current location, so the law application may still in part mentioning that meta-dynamism. Thus, this meta-dynamism is in mutual constraint with other current quantities in the law. Intuitively, the way the past affects the present is being made to be consistent with the present. However, recall that this does not come out of the blue at the current time. This making-consistent is just the current instance in a making-consistent that has already being proceeding all along during the preceding evolution.

### 4.3. Discontinuities and (Non-)Ubiquity

The toy systems in Section 3.11, Section 3.15 and Section 3.16 contain discontinuities. The meta-dynamism is sharply confined to the “cylinders” in those examples, being completely absent outside the cylinders but possibly intense inside, with no gradual transition. Concomitantly the values of the ordinary quantities and their derivatives can discontinuously jump across the cylinder boundaries.

I leave it to further work to establish whether such discontinuities should simply be accepted, or should be replaced by gradual transitions in a completed theory. For instance, there may be a way, given suitable provisions in meta-dynamic laws, of having the intensity of meta-dynamism rise gradually from and fall gradually to zero (with respect to time and space), and to be exactly zero across the whole of one spacetime region but non-zero in a neighbouring region. Here, to achieve perfect smoothness (infinitely differentiability everywhere in its domain, so there is no discontinuity at any level) we would need non-analytic real-valued functions. (These are real-valued functions that are not everywhere expressible as a power series in a certain way, in fact as a Taylor series [63]. An example of such a function is f(r)=0 for r≤0 but $=e^{-1/r}$ for r>0. It is smooth throughout, but fails analyticity at r=0, because all derivatives are zero there, meaning that the Taylor series expansion around that point would deliver zero for r>0 as well as r≤0.)

These issues connect with deep, controversial, general questions about physics, such as whether spacetime itself is discrete or continuous, or whether the universe (in its spacetime and dynamics) is largely continuous but has some discontinuities. Bound up with these issues is whether discontinuities and non-analytic features appearing in current physical theories (e.g., to describe phase transitions or quantum collapse events) are merely a symptom of the models being just useful calculation tools to make good-enough predictions about reality, or are intended to be approximate descriptions of reality itself, or are actually intended—ultimately, when completed—to describe reality accurately. Worries about discontinuities of meta-dynamism, etc. go away if space and time are themselves discontinuous. See, for example, the discussion of discreteness in [64], especially with respect to loop quantum gravity (see, e.g., [65]) and the causal set approach (see [66] for review). It is made clear in [64] that discreteness of volume and area operators in loop quantum gravity does not guarantee that spacetime itself is discrete, but the discreteness of such operators may be enough for the purposes of the present discussion, and for the continuation of it just below, in Section 5.1.

## 5. Discussion and Additional Further Research

### 5.1. (Non-)Ubiquity: Of Consciousness and of Meta-Dynamism in General

The discussion in Section 4.3 prompts, as a special case, the question of whether it is physically possible for there to be extended regions in the universe where, throughout, there is absolutely no consciousness, not even the tiniest degree, though there are immediately neighbouring regions where there is some consciousness. For instance, can consciousness be completely absent outside brains, though present inside some brains at some times? These questions engage with the important philosophical issue of whether some form of panpsychism is correct, i.e., basically whether consciousness, to *some* degree at least, is ubiquitous in the fundamental fabric of the universe, though serving as the basis for particularly marked forms of consciousness in, for instance, human brains and possibly within other complex entities.

Although we have raised an MDyn-specific version of the issues, the issues face most theories of consciousness in some form or another. Certainly, any purely physicalist theory must ultimately be consistent with the continuity or otherwise of spacetime and of the mathematical functions that are countenanced by the underlying assumed physical theory of the universe.

Analogous to the panpsychism issue for consciousness is the issue of whether meta-dynamism in general is widespread in the universe—even everywhere, possibly. But this (quasi-)ubiquity question is much less pressing than in the consciousness case. There seems to be something conceptually remarkable about saying that there is at least an extremely tiny degree of consciousness everywhere in spacetime. By contrast, once one has accepted that there may be meta-dynamism of some sort somewhere, there seems to be no particular *conceptual* bar to thinking it might be everywhere, in possibly a wide variety of forms.

### 5.2. The Additional Conjectures and Basic Forms of Consciousness

In the description of the philosophical side of MDyn, a Sufficiency Conjecture was proposed (Section 2.7), and six Adjuncts to it (Section 2.8). According to these conjectures, there is a core form of meta-dynamic auto-sensitivity underlying all consciousness, and conscious episodes can get close to consisting only, and environment-isolatedly, of this core; where, the “purer” they are in terms of this dynamism, i.e, the closer they get to this core, the “purer” they are *also* in terms of phenomenal content—both conceptual content and feeling, and both content about the external environment of the episode and content about matters internal to the episode. However, when close enough to the core, any conceptual matters drop away and we are left just with non-reflective feeling. (Note that this is still all staying within the physical realm as extended by MDyn).

We can say a little more about the core meta-dynamism underlying a conscious episode. It is itself part of the dynamism that the auto-sensitivity of the process is sensitive to. This sort of *internally auto-sensitive meta-dynamism* is illustrated in the toy systems in Section 3.15 and Section 3.16 (and some of the earlier ones). We also saw there that such meta-dynamism can be partially *auto-sustaining*, and that it can become almost pure in the sense used in Section 3. We can now unite that sense of purity with the one in the previous paragraph. That is, the core meta-dynamism suitable for consciousness has no dynamic dependence on anything other than itself, and in particular no involvement of ordinary state or of base-level dynamism. All we have is *meta-dynamism being internally sensitive to itself* in some suitable way. However, what it is for the meta-dynamism to be “suitable” for consciousness may involve additional conditions, yet to be elucidated. For instance, it may need to be internally structured in a certain way, possibly a complex way (though there is no particular reason to think it has to be complex).

With Adjunct 1C on board, we get the proposal that the purer that an episode of consciousness is, the more it consists just of the core feelings, and perhaps just of the feel of own continuing existence. The “own” in “own existence” does not (necessarily) allude to any higher-level, complex, personal self, but just to the very experience being discussed (recalling Section 1.2 on the non-egoic potential within MDyn). However, going back to the caveat above, we note that if extra structure in the meta-dynamism is needed for it to be suitable for consciousness, then there might be some sort of concomitant *experienced* structure or additional phenomenal quality in the consciousness. A special case of this will be mentioned in a moment. Further additional aspects of experience could arise from small interactions with the environment.

The just-mentioned special case is as follows. There is reason to think that there could be a basic form of pleasure or pain as a possible addition core consciousness in an almost-pure episode of consciousness. This claim needs development in further research, but its motivation is that resistance by the meta-dynamism to its own self-affecting, i.e., negative feedback in that affecting, could constitute *core pain*, or at least *core discomfort*, while acceptance and encouraging of its own self-affecting, i.e., positive feedback, could constitute *core pleasure*, or at least *core comfort*. Of course, given the assumption that in the middle of the episode the activity is reasonably stable—neither dying away yet nor exploding into ever-increasingly massive activity—the process also intrinsically inhibits the effects of such negative or positive feedback, including here the possibility that each type of feedback inhibits the other. A related proposal, probably more plausible, is that there is no core (dis)comfort in the exact core as such, but rather it can exist in enrichments of the core. Such enrichments might be composed of further, “optional” internal structure in the auto-sensitive meta-dynamism, or impurities as above, or both.

The mention just now of dying away brings us to the point that an important sort of potential impurity arises from the need for us to allow a conscious process to stop sometime. Then, unless this could happen through additional effects internal to the process, we would need to allow some interaction with the world outside itself. This does not necessarily mean spatially outside, if we refine our treatment to allow processes to be concerned with only certain aspects of state at any location.

In talking about pure, minimal or core consciousness I do not directly address notions of pure, minimal or core *self* or *subject* [21,31,67,68], especially as MDyn recognizes the possibility of non-egoic consciousness (Section 1.2). In fact, the above-claimed core, maximally pure consciousness is further claimed to be non-egoic. Furthermore, if I were to address selves or subjects I would address them as real or illusory objects (actual entities in the physical world or as entities imagined as existing by consciousnesses) not as more abstract matters such as, for instance, a mere potential for epistemicity [69]. (My selves would thus be more like subjects in a broad, everyday sense as mentioned by that article.) I do address a notion of pure self in Section 5.4.4, because it may support a notion of pure consciousness. But the relationship between selves and consciousness is a tangled one.

Almost-pure forms of consciousness are mainly put forward in this article as a matter of sheer physical possibility without any strong claim of their likelihood in practice. But I do in fact go further and suggest that, if MDyn is on the right track, then the possibility is very real—we should be surprised if it turns out *not* to be realized in practice. The work on minimal/pure/core/content-less forms of consciousness in the consciousness literature is partly motivated by reports of altered states of consciousness, as induced by drugs, meditation, mystical experience, etc., or in claimed states of “waking sleep” [18,67]. But it could be much more mundane. It could even be that one fleetingly goes through a state of almost-purely core consciousness in falling-asleep or waking-up, for example. The very simplicity of core consciousness may explain that most of us, at least, do not have any memory of going through such a pure state. Indeed, from the point of view of our efficient, evolved interaction with the world it may be pointless to remember it, so the capability has been evolved to be difficult to recall (or induce).

An additional speculative but analogous possibility is that there may be or have been simple biological organisms that are halfway between being entirely lacking in consciousness and having some simple, useful form of outer-directed consciousness, and whose phenomenality is of a largely core sort. Such phenomenality could arise as a side effect of other evolutionary developments, perhaps because certain pre-phenomenal forms of meta-dynamism are useful, or because core pleasure/pain as above is functionally useful, Then, having arisen, it could lead to more comprehensively useful, richer forms of phenomenality.

### 5.3. From PRAIS (and Sufficiency) to Restricted PRSC?

Let us put the Sufficiency Conjecture and its Adjuncts aside for the moment. Independently of these, there is an argument that suggests that if a process has PRAIS throughout, realized by meta-dynamism as proposed in this article, then it also has a restricted, varied form of pre-reflective self-consciousness (PRSC, see Section 1.9). Specifically, the argument suggests it has *pre-reflective consciousness of its own meta-dynamic auto-sensitivity*. This is consciousness of something intrinsic to the process itself, and so one could call it a restricted sort of “self-consciousness”. But we cannot conclude that it is a form of what one normally means by that term, as we cannot conclude that it is a consciousness of the process’s being-conscious. This is because we have no claim that the auto-sensitivity by itself constitutes consciousness—other ingredients may be needed for that, if we are not adopting a Sufficiency conjecture. The argument is as follows.

There is no reason to make a fundamental distinction between dynamic influences coming from outside the process and dynamic influences within. When the process is conscious of an external entity such as a red rose, then somehow the internally reflexive meta-dynamic auto-sensitivity throughout the process, in a particular form that is causally (i.e., dynamically) modulated by the external input, constitutes pre-reflective consciousness of the rose. But this same internal activity is also modulated (through meta-dynamic auto-sensitivity) by the very meta-dynamism caused in part by the input. Recall here that the internally reflexive meta-dynamic auto-sensitivity intuitively involves an indefinite ladder of meta levels, so the ladder from its bottom legs intruding into the outside world—the base-level dynamism coming in from visual input—is similar to the ladder from the first rung of meta-dynamism going upwards. It is difficult to see why the internal activity should be considered any less to be pre-reflective consciousness of its own meta-dynamic auto-sensitivity as being pre-reflective consciousness of the rose or of its dynamic effect on the process. It is all one unified pre-reflective consciousness of something, where the something is unifiedly the rose, its affecting of the process, and the internal doings of the process.

This argument is not put forward as final, and certianly needs further refinement and exploration, but it at least suggests that, even though PRAIS itself does not incorporate any sort of self-consciousness, it leads to a specialized variety of it.

But now, if we introduce the Sufficiency Conjecture, we get that the process’s consciousness at any moment of its prior meta-dynamic auto-sensitivity is thereby consciousness of (at least) its own prior pre-reflective consciousness, because that auto-sensitivity just is its prior pre-reflective consciousness (or possibly more than that). Part of the thinking here is that, in being pre-reflective, the consciousness in the process does not involve conceptual construal of the rose, the dynamism or anything else *as* anything, so we are free to substitute a mention of the process’s consciousness for that mention of dynamism, as they are identical, so we conclude that the process has *pre-reflective consciousness of (at least) its own (prior) pre-reflective consciousness.* This clearly is something one should be happy to call an interesting, restricted form of PRSC. We called it *auto-pre-reflective consciousness* for short in Section 2.1.

For the argument to work, we need to make the assumption that at each moment, what the process is auto-sensitive to includes the previous PRAIS itself (not just some more limited aspect of the process), at least in some recent portion of the process history. But it is natural to assume this, because if the process is sensitive to previous dynamism, there is no particular reason to suppose it is not sensitive to the meta-dynamism within than dynamism, and no particular reason to suppose that it is not sensitive to the special sort of meta-dynamism that is sufficient for consciousness (and it may even be that that all the previous meta-dynamism is in fact of that sort).

We have an additional if still provisional argument to auto-pre-reflective consciousness if we adopt Adjunct 1C in Section 2.8. This postulates that any experience includes a pre-reflective feeling of its own continuing existence. Suppose, as I believe is reasonable, we can take feeling-of to be a special case of consciousness-of. This consciousness is pre-reflective. So, the experience is pre-reflectively conscious of own continuing existence. This is a way of being pre-reflectively conscious of itself, which plausibly implies being pre-reflectively conscious of its own pre-reflective consciousness, given that the experience is crucially composed, at least in part and perhaps wholly, of pre-reflective consciousness.

I say “plausibly” here for fear that a problem that arises for reflective consciousness has an analogue here. One can be reflectively conscious of an apple without being reflectively conscious of its core, so, we should analogously be careful of assuming that pre-reflective consciousness of something implies such consciousness of a part. However, a special case of the situation we are reasoning about is that an experience might be composed of nothing but pre-reflective consciousness, in which case we have a trivial inference to make. So at least in this case we have pre-reflective consciousness of own pre-reflective consciousness. But one can now appeal to continuity of the general case with the special case, especially if we can assume that case can be arbitrarily close to the special case, to enable ourselves to say that pre-reflective consciousness of the whole experience implies pre-reflective consciousness of its pre-reflective aspect.

In sum, we have the following, at least in the presence of extra assumptions that remain to be fully elucidated: an argument that the meta-dynamically realized PRAIS assumption by itself, without any Sufficiency conjectures, implies a specialized form of PRSC; an argument that that the meta-dynamically realized PRAIS assumption in concert with the main Sufficiency Conjecture implies a more interesting, restricted form of PRSC that we call auto-pre-reflective consciousness; and that if we further add Adjacent 1C we get an additional confirmation, relying on PRAIS but not any assumption of meta-dynamic realization, that a conscious process is auto-pre-reflectively conscious. An interesting point here is that we are being careful to isolate auto-pre-reflective consciousness as a special case of PRSC, whereas PRSC is typically discussed as pre-reflective consciousness of one’s overall, typically reflective, consciousness.

### 5.4. Interplay with Proposals by Others

Here I briefly consider how MDyn could be enriched by ideas from proposals by others about consciousness or related matters, or, conversely, how ideas concerning meta-dynamism from MDyn could contribute to the further development of others’ proposals. In Section 5.4.1,Section 5.4.2,Section 5.4.3, I focus on three contemporary approaches, the Integrated Information Theory of consciousness (IIT) [7,60], Kremnizer and Ranchin’s proposal [62] concerning objective quantum collapse guided by “quantum integrated information” (which is consciousness-related but independently interesting), and the Orch OR (Orchestrated Objective Reduction) theory of consciousness [70]. On the other hand, in Section 5.4.4, I mention possible interplay with the much earlier work of J.G. Fichte.

#### 5.4.1. Cross-Fertilization with Integrated Information Theory

IIT is an interesting theory to discuss in the context of MDyn, because both theories seek to arrive at physical formulations of conditions for consciousness from a starting point of philosophical consideration of the intrinsic nature of phenomenology, and both explain phenomenality in terms of aspects of causal structure. However, they differ markedly in the paths taken: MDyn reifies dynamism and introduces meta-dynamism, while IIT does neither; MDyn considers causation (dynamism) at the microphysical level whereas IIT considers it as operating at any chosen level of description; and MDyn predicates consciousness on the presence of a certain type of causation (i.e., suitable auto-meta-dynamism) whereas IIT predicates it on a measure of the overall integratedness of the system. Nevertheless, such a measure could be useful to MDyn, and in the converse direction, something that is at some level MDyn’s meta-dynamism could be introduced into IIT. The latter move could even help IIT against a certain type of objection. The following comments spell out some possibilities on these lines.

IIT has it that a system’s degree of consciousness is given by its “Φ” value. This value is a measure of the extent to which the causal interactions over time within the system are integrated as opposed to being a matter of subsystems working more independently. The precise details are not needed for the present comments. “Causation” in the context of IIT is a matter of conditional probabilities of sequences of states, where a state is ultimately a matter of ordinary physical quantities. So ITT, unlike MDyn, has no reliance on MDyn’s claim that there must be more to consciousness than the trajectories of (ultimately) ordinary physical states within the system. Because IIT does not require a conscious system to involve any meta-dynamism as defined by MDyn, IIT cannot be a correct theory of consciousness if MDyn is at least roughly on the right lines. Φ might well measure something important to do with consciousness, such as how richly unified it is, but no level of Φ, no matter how high, could itself guarantee any degree of consciousness at all.

However, it is still conceivable that, if MDyn is true, some level of Φ is a *necessary* condition for consciousness. Thus, it would be possible to propose that MDyn needs to be enriched by adding this necessary condition to MDyn’s existing Necessity Condition concerning meta-dynamism, and correspondingly adding a condition concerning Φ into MDyn’s Sufficiency Conjecture, as well as using Φ to measure something about the strength or richness of the consciousness episode. It is even conceivable that the as-yet-unresolved question about what particular sort of meta-dynamic auto-sensitivity MDyn needs is answered just by saying that Φ needs to be high enough, although I do not specifically propose this and have no argument for it.

Another possibility is that a measure related to Φ could play a role in the gating expressions in meta-dynamic laws, as suggested in Section 4.1.2, so that it facilitates (or is even necessary for) the arising and falling-away of meta-dynamism and hence consciousness, even if not needing to remain present for meta-dynamism in general or consciousness in particular to be maintained throughout a process.

Note that the importation of Φ into MDyn would raise the question of what sort of causation is involved in the imported Φ. An immediate possibility would be just to let it be defined as it is at present in IIT, without regard to what MDyn means by “causation” and in particular without involving meta-dynamism (meta-causation). However, a more interesting and coherent (indeed, “integrated”) proposal would be, on the one hand, (i) to replace the patterns of causation that IIT relies on by patterns of causation in MDyn’s sense, i.e., dynamism at the fundamental physical level, and, on the other hand, (ii) to enrich Φ by having it consider all forms of that dynamism, meta-dynamism included.

In such an importation of Φ into MDyn we would *not* thereby be importing the panpsychic element of IIT. This element is that, unless one applies a threshold to Φ below which there is no consciousness, it follows that virtually every system of any significant complexity has at least some degree and type of consciousness. In the importation, this would no longer be the case even if a threshold were not applied, because the requirements of MDyn might block panpsychism anyway.

Conversely, ideas concerning meta-causation from MDyn might possibly be exported into IIT, provided that the concept could be defined in terms of the type of activity profiles used by IIT in its explication of causation. Thus, the idea is have a “meta” version of causation as conceived by IIT, not to use MDyn’s physical meta-dynamism. Then, the IIT meta-causation could, for instance, contribute to the calculation of Φ. If one does not believe that MDyn is on the right lines, one could refrain from imposing meta-causation as a necessary condition for consciousness to exist. But involving meta-causation somehow in IIT could help IIT proponents to defuse some criticisms (see, e.g., [60]), including that IIT is over-liberal in its attributions of consciousness.

#### 5.4.2. Cross-Fertilization with Objective Quantum Collapse Guided by Integrated Information

Kremnizer and Ranchin [62] (henceforth K&R) propose a collapse model (or collapse theory), i.e., a mathematical characterization of a dynamic process whereby the smoothly developing quantum wave relatively abruptly (if still smoothly) collapses (into particular alternative physical possibilities that it can be interpreted as jointly representing) [71]. Collapse models integrate collapse seamlessly into overall quantum dynamics rather than leaving it as a separate, unexplained matter. K&R’s specific proposal involves a measure of integrated information related to IIT’s Φ but expressed entirely in quantum-physical terms. Thus, the interplay with MDyn is partly analogous to that in the IIT case.

K&R’s measure, while still labelled Φ, is not intended to be a quantum-theoretic explication of IIT’s own measure, but is instead a new measure that is intuitively analogous to IIT’s Φ. K&R call this information the Quantum Integrated Information (QII) of the system. Being objectively defined at the fundamental physical level, QII and its use in affecting the dynamics of collapse is more directly relatable to MDyn than IIT’s concepts themselves are. This is despite the fact that K&R do not intend their model as a definite explanation of consciousness, but rather as an account of how consciousness could have a role, indirectly, in collapse. On the assumption that consciousness is reflected in QII, consciousness can precipitate or otherwise help control collapse. There are then possibilities for ideas from MDyn to enrich the definition of QII and/or (via MDyn-like gating expressions) to refine the extra QII-based term in K&R’s modified Schrödinger equation, and conversely for a measure such as QII to refine the way meta-dynamism is controlled in MDyn.

MDyn does not itself relate consciousness to collapse, whether to say that consciousness somehow affects collapse—see [72] for a recent discussion—or to claim that collapse is constitutively involved in consciousness, as in the Orch OR theory. Nevertheless, there are motivations for MDyn-inspired meta-dynamic modifications to K&R’s model. For instance, if MDyn is right that meta-dynamism is necessarily involved in some way in consciousness, and QII measures consciousness, then QII should be suitably sensitive to meta-dynamism.

Conversely, the K&R model, if plausibly correct about how collapse happens as a function of QII, is a candidate for the detailed mathematical framework to use in further development of MDyn, as MDyn is most at home with versions/interpretations of quantum theory that absorb collapse into the general dynamics. If, also, QII does measure the strength or richness of consciousness, and/or high QII is a necessary condition for consciousness, or facilitates or is required for an episode of consciousness to arise, it could be given a role in laws separate from the role that the model gives it, for instance by having it as an argument in a new type of gating expression in MDyn.

#### 5.4.3. Orch OR’s Objective Reduction

Orch OR [70] proposes that objective quantum-state reduction (collapse) can “result in moments of conscious awareness and/or choice”. This opens up possible interactions with MDyn. MDyn does not propose that consciousness is constitued of, or a result of, collapse events, orchestrated or otherwise. However, suppose that both MDyn and Orch-OR are roughly on the right lines: that some form of meta-dynamism is needed in conscious processes, and conscious processes consist of one or more collapse events. Then, presumably, we must have meta-dynamism playing a role in how at least some collapses happen or proceed. Potentially, this role could be partly or wholly within an individual collapse event, considered as a short-lived continuous process. The process could be meta-dynamically sensitive to its own dynamism. Or, a collapse could even be meta-dynamically sensitive to the dynamism in preceding non-collapse state evolution and/or to prior collapse events.

Importantly, not all collapse events would need to involve (significant) meta-dynamism, and hence it would not need to be that all have a connection to consciousness. Thus, meta-dynamism could provide an important element of selectivity to Orch OR. The modification would lessen or eliminate the need to talk about all collapse events being, to a small degree at least, occasions of consciousness (events of “proto-consciousness” [70]). Hameroff [73] proposes that one factor that could usefully regiment the collapses that are involved in consciousness is an “envelope” of a particular sort of neural activity in the brain. Further control, and control of a qualitatively different sort, via meta-dynamism could be a useful adjunct to the envelope idea.

As a specific, detailed point concerning the control of individual collapse events, we note that Orch OR says that collapse takes place in average time τ inversely proportional to the gravitational self-energy $E_{G}$ of the superimposed states. (This self-energy arises because of different mass-distributions of the different states in the superposition, resulting in different spacetime curvatures for them.) One immediate suggestion would be to make the average collapse time dependent on meta-dynamism as well as gravitational self-energy. However, also, the theory leaves the specific time for collapse arising on a specific occasion to be random. Therefore, there is scope for adding meta-dynamism-dependent refinements to the theory, affecting the detailed timings in a non-random way.

#### 5.4.4. Fichte’s Science of Knowledge

One intriguing historical angle to be explored in future research is a set of potential parallels with the *Wissenschaftslehre* or *Science of Knowledge* [henceforth SoK] developed by the 18th/19th-century transcendental idealist J.G. Fichte. (I am grateful to an anonymous reviewer for the suggestion to attend to Fichte. My comments are on the basis mainly of [8], a translation of an exposition by Fichte of SoK, with some matters, including about later developments of SoK, illuminated by [69,74,75,76]. Page references in double square brackets refer into [8].)

Only a brief, highly provisional indication of the possible parallels and non-parallels can be given here. (This is especially so because of the difficulty of interpreting Fichte’s work. Even the translators’ Preface to [8], on pp.vii–xx, while according high praise overall, is critical of Fichte’s vagueness, inconsistency and failure to explain key matters.) SoK and MDyn differ fundamentally in that the former is an idealist theory whereas MDyn is realist about a mind-independent physical world. However, for both accounts, consciousness and its underpinnings are a matter of the way *there is an activity that acts upon itself* and is sensitive to itself, including to its own self-action/sensitivity. Self-acting activity in SoK occurs at the very basis of any individual, personal self, and is all there is at that level, the level of the “absolute self”. There is just “pure” activity (pp. xi, 97), a “doing” merely (p. 66), one that is its own product or “deed” (pp. 42, 97). The activity’s self-action appears therefore to be entirely unmediated, allowing a possible analogy between the activity and dynamism, and between Fichtean self-activity and MDyn’s auto-meta-dynamism. Notice here that if, in MDyn’s terms, a process is sensitive to something X, then X affects the process, so a being-sensitive-to is at the same time an affecting or acting-upon. There are also passages in [8] where meta-causation (meta-dynamism) is more explicitly indicated, as when the self’s pure activity is said to cause its object-directed activity (the latter being the activity that causes an object) (p. 231/2). No mediator is mentioned for that causation by the pure activity, so we do not, ostensibly, have a case of mere chained causation.

One question for further exploration is whether there are SoK parallels for MDyn’s core and [almost-]pure consciousness. The absolute self is probably not a candidate. [74] says that the absolute self is only an abstraction, while [69] implies that the absolute self is not an object but merely the potential for or locus of epistemicity or phenomenal consciousness. Zöller [76] states that the absolute self lacks all consciousness. However, SoK (p. 216) says that “The more a determinate individual [or individual ‘empirical self’] can think away of himself, the closer does his empirical self-consciousness approximate to a pure self-consciousness”. We may see here something akin to core consciousness that can exist to various extents of purity but is considerably more than the absolute self. At any rate, a self in SoK possesses various basic feelings that are fundamental in the theory, such as a feeling of compulsion to act (pp. 211, 261, 266), a feeling of limitation (pp. 21, 266), longing (p. 265), and, notably, a non-conceptual “intuition” that the self has of its own existence ([75], p. 261). This last is highly analogous to a specific core feeling that MDyn proposes in Section 5.2, the feeling of own continuing existence.

A salient disanalogy between SoK and MDyn is that Fichte [8] often emphasizes that, in talking of activity, he is abstracting away from temporality (pp. 129–131). Nevertheless, much of the time he talks about the activities of the self in highly temporal terms. Though he may mean these terms only metaphorically, the idea that the activity is at root atemporal is hard to reconcile with its being a “doing”. Thus, for the sake of bringing out possible parallels I have taken the activity to be one that unfolds in time. If this violates the intent of SoK, it just means that the parallels are more metaphorical than portrayed above. They are still of value in the landscape of consciousness theory, as it might suggest possible atemporal variants of MDyn or useful temporal, physically realist variants of aspects of SoK.

### 5.5. Conscious Machines

The multiple realizability of MDyn (see Section 1.8) extends to computational artefacts, as long as their implementation in a physical device provides suitable meta-dynamism. According to MDyn, it is not enough just to run a sufficiently complex and cunning algorithm in a conventional computational substrate such as a present-day computer. The dynamism in the transitions between the physical realizations of the computational states needs to have a direct, meta-dynamic effect upon the computations (or upon other such dynamism). A consequence of this is that if one ran a computer simulation of a conscious system, a conscious brain for example, and even if the simulation covered the minutiae of meta-dynamism, the result would not be conscious unless the simulation algorithm itself were physically implemented in a suitable meta-dynamic way. Moreover, even if it were implemented in a way that amounted to consciousness, that consciousness might not be the one simulated: it would be at an entirely different level of description of the overall system. In terms of thought experiments such as the Chinese Room [77,78], MDyn agrees that the mere bandying about of symbols in, e.g., the Chinese Room is not itself sufficient for consciousness.

### 5.6. Simplicity

Meta-dynamism presents conceptual subtleties and complications, and introduces formal complications such as those brought out by the inclusion of the web of constraint in Section 4.2. Furthermore, the way that a particular meta-dynamic law or system equation works, such as in the case of our toy-example equations in Section 3.11, Section 3.15 and Section 3.16, can conceptually complicated. Nevertheless, those equations are in themselves quite simple formally. MDyn presents the promise that it is simple to describe the auto-meta-dynamism needed for consciousness.

There is, thus, a sense in which physical systems that exhibit relatively basic forms of phenomenality may be rightfully regarded as simple systems. Perhaps the best way to put this is that meta-dynamism in general introduces considerable complications into physics, but what’s needed for phenomenality on top of that general framework (particular marshalling of meta-dynamism) is simple. Of course, as we scale up to more and more cognitively and behaviourally complex conscious systems, the overall physical complexity increases in parallel, and in particular the way in which phenomenality is intertwined with the cognition, perception and action will be more and more complex. In a neurophysiological context, the physical-mechanism aspects supporting phenomenality may reside in a complex, convoluted way within a large neural network, for example (while we should not put aside the possibility raised by MDyn that complex meta-dynamism could exist inside parts of single neuron, given the huge complexity of, for example, the dendritic fields of important types of neuron). But the point is that not much physical complexity is needed to step from the flow of meta-dynamism in general to the first rung of the ladder of phenomenality.

A qualification is needed here. As has been occasionally mentioned, the physical situations that give rise to an episode of experiencing, in reality (as opposed to our toy examples), or that lead to its demise may be complex: the claimed simplicity is thus mainly about what is needed for the progress of the experience once started.

The claim of simplicity might be thought to be in tension with the inherent reflexivity of consciousness in MDyn’s view (as provided by the Sufficiency Conjecture plus Adjuncts, Section 5.1 and especially Section 5.2). One might ask: wouldn’t consciousness that is directed at simple external objects and does not have any sort of auto-consciousness be a simpler matter? With reflexivity of consciousness on board, the MDyn answer is sure, this would be simpler if possible, but it’s not:- Consciousness is inherently reflexive, so, in fact, an episode of experience that is directed at something outside (it is conscious *of* something outside itself), or consciously acts on its external environment) is a *more* complex matter than an experience that does not have such a feature.

### 5.7. Further Issues

The current framework has a mixed nature in crucially involving holistic dynamism regions as well as dynamism at individual spacetime locations, and keeping to laws whose applications are thought of as pertaining to individual locations, even though the laws can refer to past regions, and even though the dynamism at the individual location can itself include time-hopping relations to past regions. Moreover, dynamism is arguably a fundamentally non-punctate matter, as it intrinsically concerns how a locations fits in with other locations, so the idea of punctate dynamism is arguably a rather artificial device included to fit in with laws about individual locations. These observations raise two suggestions for further development.

First, punctate dynamism could be removed without enormously affecting the remainder of the framework. In particular, no crucial claims in this paper rest on the ability for current state to be in mutual constraint with current punctate dynamism as opposed to a past, temporally abutting region of dynamism. If it should turn out that reference to current dynamism is needed in some sense, then what could be introduced is reference to a surrounding region of dynamism even though it pokes into the future.

Second, and more radically but cleanly, one could make a major shift in the ontology and in the mathematical formulation, and abandon punctate state and laws entirely, using only non-punctate regions and laws about state over them. Punctate states would become mere approximative and heuristic abstractions, useful for some but only some approximative theoretical or practical purposes. This may not only serve the notion of dynamism better, but also fits with Bohm and Hiley’s observation in [59] (p. 374ff) that regions may better serve quantum theory, and with use of regions in work on quantum gravity (see, e.g., [64,65]). The shift would also fit with the more general idea that temporal non-locality in physics could profitably be given more central attention, more equal to the attention given to spatial non-locality [28].

The thrust of Section 4.2 on the web of constraint suggests the possibility of just identifying dynamism with constraining. Under such a view, constraining would be physically reified and would itself be able to enter into constraining with physical aspects of the world (meta-constraining). Aside from the metaphysical issues here, mathematically speaking it may be tantamount to simply identifying the dynamism at a location with the web of constraint at that location, and a dynamism region with the overall resulting web. While I am drawn to a view of time-flow as physically real (with a close relationship to dynamism as portrayed as a pushing-forward), a version of MDyn with dynamism identified with constraining would be more compatible with a static-block view of the universe where there is no time-flow other than as an illusion in consciousness. (See [58] for metaphysical theories of time, including with reference to physical theory, and to entropy in particular.) However, the notion of constraining as itself subject to meta-constraining, with (meta-)constrainings being first-class physical citizens, needs further work.

In considering the toy systems in Section 3.11, we saw that there could be more than one “solution” concerning the development of behaviour as far as the equations of change of the system are concerned, but that considerations of maximal evolutionary consistency can plausibly secure a particular solution. It should be stressed, though, that equations of change trump evolutionary consistency when there is a conflict between them. Additionally, these two possibilities concerning selection amongst candidate solutions are just a special case of a broader question for future research, namely the extent to which the proposed meta-dynamic laws lead to more complex conflicts between constraints, to the extent that a theory is needed about how such conflicts are resolved and a particular solution “selected”, or a range of solutions survives and one is stochastically or completely arbitrarily “chosen”.

This is reminiscent of the idea of quantum wave collapse, of course. If the comments in Section 5.4.2 on the possible links between meta-dynamism and collapse turn out to have some truth, it is then not beyond the bounds of possibility that a constraint-resolution feature that operates in complex meta-dynamic scenarios could be involved in the direction a collapse takes.

## 6. Conclusions

The current aim of MDyn is to provide a model of consciousness in the basic sense of sheer phenomenality. While the proposed meta-dynamic behaviour in consciousness is a radical addition to philosophy and physics, is very strange intuitively, and is highly complex in terms of how ordinary quantities, base-level dynamism and meta-dynamism relate to each other in general, at the same time, MDyn holds the promise of being able to provide a model of phenomenality that is at its core very simple. It involves a meta-dynamic auto-sensitivity that is describable in a relatively simple way in equations. As part of this, it supports, and give solid flesh to, the idea of an (almost-)pure episode of consciousness whose only significant activity is to react to itself, and indeed where this auto-reaction, *is*, to exaggerate only slightly, itself.

MDyn is multiply radical. Giving meta-dynamism, or any form of meta-causation (in the sense of causation with causings as relata, whether or not all this is at the basic physical level), a central constitutive role in consciousness is philosophically a radical and apparently novel proposal about consciousness; and this is quite apart from the fact that meta-causation, where at the basic physical level of meta-dynamism or some higher scientific or conceptual level, is at best a minority concern in the philosophy of causation in the round, putting aside consciousness. Moreover, on the physical-theory side, MDyn is of course radical in introducing dynamism as a first-class citizen into physics, with the accompanying introduction of an entirely new order of laws—meta-dynamic ones—giving the overall set of laws the peculiar, reflexive potential of mentioning the dynamism that consists of their own ongoing or past application.

The article sets out a rich framework for further research. Various points where work is necessary or desirable have been noted in passing throughout the article, and I do not summarize them here. Clearly, there is a need for major developments in understanding the nature and mathematics of dynamism, and in the details of gating expressions, temporal non-locality, the time-reversal asymmetry, the web of constraints, dynamism-instance valuation functions and auto-sensitivity. A big future project is to modify the account to be suitable as an extension to a quantum-physical framework, and to determine specific changes to specific law-equations. Moreover, of course, as the philosophical side develops, this may require other major changes on the physical-theory side.

Clearly also, MDyn needs ultimately to be developed in the direction of accounting more fully for consciousness-of and for broad, complex, conscious systems, not just accounting for sheer phenomenality in principle. One particular deep challenge here (and one that faces all theories of consciousness) is how to account plausibly for the fact that we have such radically different modes of conscious experience (visual, auditory, emotional, etc.), if all the modes are based on basically the same mechanisms if in different precise arrangements. MDyn has proposed a natural basis for certain basic feelings (including feeling of own continuing existence and “core [dis]comfort”), but it still remains difficult to see why the qualitative, experienced differences between modes are as radical as they are.

MDyn’s concepts might be borrowed not only by other physicalist theories of consciousness (as in Section 5.4), but might also be borrowed to illuminate a number of current issues in philosophy, even by someone who does not accept the theory as a whole. Meta-dynamism, apart from providing a mechanism for concepts such as for-itselfness, mine-ness, ipsiety (thisness) and self-intimation in the theory of consciousness, could have more specialized uses such as supporting the idea of direct causation-based perception of causation as has arisen in power theory (see Appendix A.1), realizing meta-acquaintance or direct acquaintance with direct acquaintance (Section 1.9), and reformulating Fichte’s ideas on consciousness and self (Section 5.4.4). Putting aside meta-dynamism, MDyn raises (in Section 5.3) the profile of phenomena weaker than pre-reflective self-consciousness as normally discussed, and in particular suggests that pre-reflective consciousness about own *pre*-reflective consciousness is especially interesting though under-discussed.

There is no claim that MDyn rests on definite, unimpeachable arguments that consciousness must involve meta-dynamism. The arguments are at least strongly suggestive, however. Moreover, there is no definite argument (as yet) that some special form of meta-dynamism is sufficient for, indeed constitutive of, core consciousness. This is presented as a constructive suggestion for further investigation. But we can make a motivational comment here. The nature of dynamism as a whole, let alone meta-dynamism in particular, may appear metaphysically and physically mysterious, except insofar as this nature is constrained or revealed by the specific assumptions and proposals made in this article. However, novelty may account for much of any air of mystery, and the nature of dynamism may ultimately be perceived to be no *more* mysterious than that of say, mass, energy, fields, etc. However, before this conflation of mysteriousness happens, the intuitive strangeness of meta-dynamism—both in its basic conception and in its consequences such as the self-swallowing web of constraint) may be the bridge we have been seeking in order to span the Explanatory Gap between the non-strangeness of the (common-sense) physical world and the intuitively strange nature of consciousness. The type of strangeness seems intuitively to be on the right track in the self-swallowing, or quality of something getting inside itself.

## Abbreviations

The following abbreviations are used in this manuscript:

MDyn	Meta-Dynamic Theory of Consciousness
QII	Quantum Integrated Information

## Appendix A

The main role of this Appendix is to sketch the arguments that suggest that meta-dynamism is needed in consciousness and that dynamism should be added as a new component in physical states. So in the course of that argumentation, any physical states mentioned, will, except if otherwise noted, be “ordinary” states in the sense used in the main text, and so will not include dynamism. Furthermore, for simplicity, the argumentation does not distinguish between different spatial locations within a process, so that the state at a given time is the overall state across all spatial locations at that time. The arguments start in Appendix A.2, after comments on another matter.

### Appendix A.1. More on Causation and Meta-Causation

This article’s usage of the term “causation” refers to something like the productivity or “[causal] oomph” that others have discussed (see the Introduction), and not to what is predominantly called causation in the philosophy of causation (see, e.g., [25,79]). “Causation” is usually cast as a relation between causal relata that concern separate moments of time and spatially limited objects, events, etc. at those moments, rather than a pushing-forward that exists everywhere at every moment. Those relata are variously held to be a wide range of things, depending on the theory—events, facts, property instances, etc., and/or types of such things. Furthermore, causation theory is typically (though not exclusively) pitched at a level much higher than fundamental physics, for instance everyday human activities, or interactions between simple objects that feature in everyday life (billiard balls colliding, etc.). The notion of causation as being between spatially and/or temporally separated events is also common in discussions about relativity theory, entanglement, and so forth. While many causation researchers work hard to achieve an objective characterization of causation, in terms such as conditional probabilities, or counterfactual worlds that are supposed somehow objectively to exist, this has certainly been difficult, especially as so often the relata rest on how humans view the world, e.g., how we commonsensically divide the world up into types, or how we take an event to be a particular instance of a particular type. My aim is not to object to such work but rather just to distance MDyn terminology from how causation is viewed in such studies, and to emphasize that I take causation in the form of dynamism to be an entirely objective, fundamental physical constituent of the world.

Meta-causation, in the sense of causation instances themselves being causal relata, has occasionally been discussed in the philosophical literature. Ehring [79] briefly covers it in an article surveying the philosophy of causation, though he calls it iterated causation. (This is a sub-optimal term as it may sound like mere chained causation, which is simply where A causes B and B causes C.) Koons [80] discusses it under the heading of higher-order causation. In Section 5.4.4 we see that Fichte [8] can be construed as talking about meta-causation. However, as Kovacs [23] notes, meta-causation under any name has been remarkably little discussed overall.

The notion of dynamism has much in common with contemporary “power” theory [81,82], where the notion of a power is, or is similar to, the notion of a disposition to do something. Dynamism at particular locations or over regions may be seen as (partly) constituting what are often called exercisings of powers. Meta-dynamism would become exercisings that are directly interacting, via their own powers, with other aspects of the world, including power exercisings. There is an existing notion of “meta-causal powers” [83], but this does *not* involve what I would call meta-causation or meta-dynamism, as it does not involve exercisings acting on power exercisings. Instead, it merely covers operations on other powers as such: it is to do with a power potentially creating, destroying or modifying some *power*, not acting directly upon a power-*exercising*. On the other hand, Klinge [84] discusses, in the context of power theory, a special form of downwards mental-to-mental causation that does qualify as meta-causation.

There has, on the other hand, been discussion, for instance in [85,86]), of whether we can be directly, perceptually conscious of causings. If one holds that perception is partly a matter of things in the world causing happenings in the brain, then one appears to be proposing meta-causation. But this line of thought is at most about how there might be meta-causal influences on our conscious states, not about consciousness itself being made of (suitable) meta-causation, which is what MDyn proposes.

### Appendix A.2. Problems Concerning Representation

As mentioned in Section 2.6, there is, I argue, no theory of representation that is both completely objective and pre-reflective. For instance, in the comprehensive survey of possibilities that Shea [48] goes through in devising a theory of representation, it is possible to discern in every one a point where it (i) requires something that would go outside pre-reflectivity and/or (ii) involves non-objective considerations about what represents what. As noted in Section 1.10, the arguments of [49,50] also militate against the possibility of entirely objective representation.

The non-objectivity in (ii) arises in the following sorts of ways (partly paralleling the discussion in, e.g., [49,50]). If, for instance, the representational relation depends only on some sort of structural correspondence between representor (the item that represents) and representee, or some sort of correspondence as defined between logical expressions and a model, then there can be no objective, physics-based matter of fact of what the representee is. If I have a representor in my brain of some some state in my brain, but that represented state happens to be structurally similar enough to a state in your brain, then I would be as much representing your state as mine if representation is based merely on structural correspondence: but that cannot work for an objective theory of *my* consciousness. (See [55] for a related argument.) If one tries to bring in the question of which particular pieces of matter are involved, demanding a particular connection between the piece the representee lies in and the piece the representor lies in, then we get into issues of substitution of matter already raised in discussing substrate replacements in Section 2.3, and questions of what are the criteria for adequate persistence of and connectivity between the pieces.

This question of connection also relates to theories of representation that rely in part on the representee having caused the representor to arise in some particular way. There are many issues discussed in this area (e.g., see Shea ibid) about what one should take the cause in the causation relationship to be. For instance, a dog may somehow cause my representor that represents the dog, but I could equally say that the light rays etc. between me and the dog, not the dog itself, cause my representor. Similarly, the causation link to my brain typically involves both less than the dog (I’m not receiving much in the way of light rays from its side facing away from me, let alone from its intestines, for example) and more than the dog (I would not be receiving any light rays unless there were a source of illumination). There is also indeterminacy in the category of the object represented: is my representation really one of a dog, or rather of a member of a broader category of things that could be mistaken for dogs? However, given all such problems, there is no completely objective fact of the matter about what is represented. Going back to the states within a conscious process, there would be no objective matter of fact that a current state is indeed representing the particular prior states or between-state causal/dynamic relationships that are allegedly represented.

There are also more or less “teleological” theories of representation that rely on representors having historically played a survival-supporting role for that system, thereby achieving a survival-enhancing ability to perform important cognitive tasks (see again Shea ibid.). But here we not only have similar issues as regards the objectivity of the causation involved, but also lack of objectivity about how tasks are identified, how useful the alleged representation relationship has been, how the usefulness can be assigned to the representation as opposed to other aspects of the organism, what survival amounts to, etc.

### Appendix A.3. Conscious Processes as Causally/Dynamically Bound

I claim that consciousness is a property most felicitously applied to “genuine” processes. Intuitively, a genuine process is one that is held together by dynamism *within* itself, dynamism that is centrally concerned with how [ordinary] states in the process’s state trajectory evolve from prior states in it, as opposed to dynamic influences from outside the process. I will call the latter “incoming dynamism”. To put the matter in terms of causation, states of a genuine process are, to a crucial degree, caused by prior states within the process rather than outside, though influences from outside can also modulate the evolution. Genuine processes are in contrast to *pseudo-processes* (see [87] for review). A common example of a pseudo-process is the shadow of someone walking. States of the shadow do not cause further states of the shadow, under reasonable accounts of causation. Rather, there is a genuine process of someone walking, and causation emanating from each state of that process leads to changes of illumination that we regard as a moving shadow. The distinction between genuine and pseudo processes is difficult to make precise and objective, partly because as already noted we must allow (in general) for even a genuine process’s unfolding to be affected by physical influences from the world outside itself. Moreover, conversely, there is *some* extent, if minuscule, of causal interaction amongst the states of a pseudo-process. For example, the lower illumination of the shadowed region at a given moment lowers the temperature there, causing some lowering of temperature in a region just outside the shadow but shortly to be shadowed, affecting those atoms’ movements and therefore affecting exactly which atoms it is that are involved in the shadowed region and therefore what that region is and perhaps how strongly it reflects light. So the genuine/pseudo distinction is a hazy one hinging on the balance of internal versus incoming dynamism. Fortunately, the present article does not require there to be a precise, objective distinction. The notion is only a way of heuristically guiding argumentation towards the conclusion that meta-dynamic effects of dynamism within the process is a good explanation for the required auto-sensitivity of processes, allowing the internal/incoming dynamism balance to vary widely. Notice in particular that the mathematical treatment in main-text Section 3 does not distinguish between pseudo and genuine processes.

It may already be quite intuitive to the reader that a conscious process must be a genuine one. It may in particular seem obvious that, if a process Q were like a moving shadow in developing almost entirely because of constantly arriving “input” from another process P, and not because of internal dynamism, and indeed the internal dynamism could notionally be eliminated without much affecting the progress of Q, then Q would not be conscious, even if P was. However, it is useful to back this intuition up with a more careful argument.

We can get a specific argument by considering the following question: can a theory about when a process is conscious be based entirely on objective conditions placed on, merely, the trajectory of [ordinary] states in the process, without imposing some requirement C on the amount and nature of the internal dynamism that (at least in part) sustains the trajectory? If the answer is No, then merely copying a trajectory could not be inferred to create a new conscious process, unless the copy somehow necessarily had to have internal dynamism that fulfilled requirement C. That the answer should indeed be No is suggested by the following thought-experiment (related to thought experiments in [1] and ones elsewhere in the literature, e.g., in [54,55].)

Suppose a process’s being-conscious is merely a matter of what the process’s state trajectory is like. We will argue that this leads to an unacceptable consequence. Imagine a scenario S1 in which there are N>1 identical brains, each sustaining a conscious process over some time interval (u,v), where the processes in the different brains are identical to each other except for being in different spatial locations. (It is easiest to imagine that there is no input to the brains, but we could allow for input as long as it was isomorphic across the brains.) It does not matter here what type of [ordinary] state (electromagnetic, positional, etc.) is included in the processes, or what portion of each brain is involved. Now consider an arbitrary division of (u,v] into *N* successive sub-intervals $(t_{k},t_{k+1}],k=1\ldots N$, with $t_{1}=u$ and some $t_{N+1}=v$. We can then consider the state sub-trajectory of the relevant neurons, etc. of brain 1 over sub-interval $\left(t_{1},t_{2}\right]$, the state sub-trajectory of the relevant neurons, etc. of brain 2 over sub-interval $\left(t_{2},t_{3}\right]$, and so on. Then, by concatenating these sub-trajectories, we have identified a process D over (u,v) that is “diagonal” across the brains. Abstracting from the question of spatial location and which particular neurons and connections are involved, D is clearly isomorphic to the process happening in each of the brains. D involves neurons etc. of brain 1 in the first sub-interval, neurons, etc. of brain 2 in the second sub-interval, and so on. Intuitively it is a strange process, but nevertheless exists in the scenario just as much as the *N* original processes do.

Now consider a different scenario S2, in which there is the same time interval (u,v) and division into sub-intervals, but one brain BM moving as follows: at $t_{1}$ it is at the location of brain 1 in the original scenario; at $t_{2}$ it is at the location of brain 2 in that scenario, and so forth; and in between $t_{k}$ and $t_{k+1}$ it moves continuously in some way between the location of the *k*th and k+1th brains of the original scenario. Let BM sustain a conscious process M isomorphic to that in each of the brains of the original scenario: it is the same process except that *M*, within each subinterval, is moving in space from one brain’s position to another. Then clearly the diagonal process D constructed in the first scenario is the same as the process M in BM, apart from such movements and the fact that the two processes involve different neurons, etc. The neurons etc. involved in D change from sub-interval to sub-interval whereas the neurons, etc. involved in the BM process stay the same throughout, and in each sub-interval D stays at one location whereas M is continuously moving.

Now, this difference between D and M should not matter to the presence of consciousness, if it is correct that whatever movements in space that a process is undergoing are irrelevant to the presence of consciousness (see Section 2.3), and it is correct that one can replace neurons in a brain at any moment by different neurons (etc.) without affecting consciousness. If one can replace even one neuron at a given instant while preserving consciousness, it is difficult to see why one cannot replace them all (in an imagined thought experiment). Moreover, of course if one can do it at one instant there is no reason one cannot do it again at another. So, M could in our imagination be replaced by D without affecting the presence of consciousness. Thus, we must conclude that, merely by virtue of of there being isomorphic conscious processes in *N* brains, there is automatically also a further one, the D above. Given that the original *N* processes are, indeed, different consciousnesses even though isomorphic, we must presume that D is also a consciousness different from all the rest. But surely it is implausible that a new consciousness can exist in this way just as a logical result of the existence of others. (The original *N* processes are different consciousnesses even if it is argued that each of them feels exactly the same to itself as every other one of them does, i.e., they are qualitatively identical, especially given the absence of any interactions with the external world. It is just that the same quality is multiply instantiated. Each instantiation is a different entity.)

Moreover, the problem is vastly more extensive than portrayed so far. It does not matter what order we consider the *N* brains in when defining D. So there is in fact not just one “extra” consciousness but N! different ones (actually more if we allow D to come back to a brain rather than involve all *N* brains but each one only once). Worse, the division of (u,v) into sub-intervals is itself arbitrary. This consideration introduces indefinitely many new possibilities.

One might seek to object to the argument on the basis that a diagonal process *D* hops instantaneously to a new place between sub-intervals. But if one is defining consciousness entirely in terms of state trajectories one would need to have a principled argument why such spatial hops would matter, if one is completely excluding from the picture any question of how states are dynamically linked together. Furthermore, recall that each D exists as much as the *N* brains and their processes do; the hopping is not a matter of pieces of matter hopping instantaneously from one position to another.

Another attempted objection might be based on the instantaneity of the imagined replacement of neurons, etc. when we imagine converting M into D. This objection could be met by noting that replacement could be done non-instantaneously but fast compared to the length of the sub-intervals. This would lead to a process D${}^{\prime }$ that deviates a little from D, but we could make this deviation arbitrarily small by making the replacements fast enough. Plausibly, small enough deviations should ensure that D${}^{\prime }$ would still be conscious.

On the other hand, the thought-experiment fails to go through if we now do allow the question of whether a process is conscious to depend to some degree on dynamism within the process. This is because the lack of dynamic connection between the segments of D in different sub-intervals could violate the needed involvement of dynamism in a conscious process. Each individual segment of D might still be a conscious process, but we would not be able to argue that the segments would combine to form a single, unified, uninterrupted conscious process.

Thus, to avoid the conclusions of the thought experiment, a theory of conscious processes needs to propose some suitable involvement of dynamism throughout the process, with no breaks. If there is even one break, then a version of the thought-experiment with N=2 can be constructed, where D’s “hop” between sub-intervals happens at the break time.

Of course, the involvement of brains in this subsection is only incidental. Similar comments could be made about systems of any type considered as possible bearers of consciousness, for instance computers running programs.

### Appendix A.4. Why Does the Dynamism Matter?

With, or without, the considerations of the previous subsection, one might readily accept *that* a characterization of conscious processes must advert to their internal dynamism, not just to their state trajectories. This presumably means that one accepts that dynamism is real, at least in a metaphysical sense.

However, if the characterization of consciousness requires dynamism, then it cannot be enough just for there to be *some* dynamic influence or other between the states, because any state might have at least an extremely tiny influence on later states for entirely incidental reasons, much as we argued in the case of a moving shadow. For instance, neurons involved in a conscious brain process would affect each other to a tiny degree through gravitation. This would be a process-internal internal dynamic influence, but would plausibly be irrelevant to the presence of consciousness (unless perhaps if Orch OR’s gravitational aspect is correct). So there must be constraints on the nature of the mutual dynamic binding of the states in a conscious process. But whatever that constraint is, one can ask: *in what way* does the constrained dynamic binding matter? What job is it doing in the constitution of consciousness, over and above any job being done by merely the states that the dynamism binds together?

One type of answer to this question could be that it is is simply a brute metaphysical (or physical) fact that the consciousness of a process is partly constituted by its inner dynamic binding: there is no further explanation or mechanism to be found. However, it is at least interesting to consider whether there are other possible answers.

A consideration pointing towards an alternative answer is provided by the notion of pre-reflective self-consciousness (PRSC) mentioned above. (This was developed in the phenomenal tradition in a way not dependent on considerations of dynamic binding within processes.) The notion raises the question of what, more precisely, is it in or about itself that a conscious experience (i.e., conscious process) is conscious of, in its “self”-consciousness. Another way this issue could be put is: what is it about itself that matters to the experience? Arguably the most general, abstract answer one can give to this is that *its own being-an-experience matters to itself.* This answer is entertainable across all the different types of consciousness that might exist under different circumstances, and abstracts over the different types of circumstance (e.g., in the external world) that the experience might be conscious of.

If that answer is correct, then *being-a-conscious-process* matters to the process itself, since an experience in this article’s terminology just is a conscious process: the two terms are entirely synonymous. There is no (hyper)intensionality problem here. We are not considering the process to be *conceiving* of itself as a conscious experience. If it were to do this, the identity of experiences with conscious processes might not guarantee that the process is thereby conceiving of itself as a conscious process (either because it does not realize that experiences are conscious processes, or because, despite realizing this, it never makes the needed step from one concept to the other). Thus, it is a crucial element of this article that it focuses on the *pre*-reflective (non-conceptual, non-propositional, non-inferential) underlying nature of consciousness, not optional reflective processes that might recruit it.

Now, as explained above, for certain reasons, I believe it is too strong, and unnecessary, to postulate auto-*consciousness* is present during an experience. In moving from PRSC to PRAIS, I have therefore gone to the weaker notion of auto-sensitivity, which does not explicitly presume auto-consciousness, but leaves the latter as something that the ensuing theory implies is present. However, this move from PRSC to PRAIS does not change the claim that being-a-conscious-process matters to the process.

Recall that we are assuming that being-a-conscious-process is a question of being a state trajectory together with some suitably constrained internal dynamism (partially or wholly) giving rise to it. Hence, given that being-a-conscious-process matters to the process itself, so does being a process that involves thus-constrained dynamic binding. This therefore provides an answer to the above question of why the dynamic binding in a conscious process matters: *it matters to the conscious process itself*.

In short, by proposing PRAIS as necessary for consciousness we are proposing both a less presumptive version of PRSC and simultaneously an answer to the question of why dynamic binding in consciousness matters.

This mattering is opposed to the unexplained, brute mattering that was entertained in passing above. It is also opposed to the idea that the dynamic binding could matter to some physical entity outside or beyond the conscious process. This other entity had better not be a conscious system, a suggestion which would create circularity, but on the other hand it is difficult to see why any sort of mattering to a non-conscious system should contribute to making our original process conscious.

Crucial also is that this mattering of dynamic binding needs to be *entirely objective*: the existence of the mattering cannot have any whiff whatever of construal by us as theoreticians. This is because (this article assumes that) consciousness is an entirely objective property of a given physical process.

The question now is, *how* does the process’s dynamic binding matter to the process? That is, what does the objective auto-mattering consist of?

### Appendix A.5. How Does the Dynamism Matter? Meta-Dynamism and Other Suggestions

Were it not for the problems with representation that we have noted, we might answer the auto-mattering question by suggesting that each state in process involves a representor that represents the prior dynamism in the process. MDyn proposes instead that prior dynamism within the process, or more precisely the dynamism within the process across some interval up to the current time, has, in its own right and as a whole, a *direct* causal effect on the current state, not mediated by the normal evolution of state over that interval. This proposal avoids the above problem that a (partly) causal account of representation, in this case of past dynamism, would have with specifying how far back a causal chain should go, and it obviates any need to consider similarity relations or the use of conceptual apparatus.

The claim that prior dynamism (over time intervals) has a direct effect, where this affecting is therefore a type of meta-dynamism, could just be adopted as another basic postulate of the theory. But it is worth providing an argument against another possibility that might be suggested. This competing suggestion recognizes the reality of dynamism, at least at a metaphysical level, even if not admitting it into the physics. However, it argues that it is enough for the trajectory of ordinary *states* over some segment of the process history leading up to a given state to have, as a unit, a direct causal effect on that state (i.e., an effect over and above the moment-by-moment evolution of state according to ordinary physical laws: rather we are now talking about a fundamentally temporally non-local influence from a sequence of past states, as a unit, on the present state). The basis for making this suggestion would be a claim that, given an [ordinary] state trajectory, it is completely determined what dynamism gives rise to it. So, someone might claim that the process’s state at *t* is sensitive to the process’s dynamic binding in some interval abutting *t* by virtue merely of the fact that it is directly influenced by the sub-trajectory of states over that interval. Or, to put it in terms of mattering, dynamic binding up to *t* matters to state at *t* as an indirect side-effect of the mattering to the state at *t* of some [ordinary] states up to *t*, without there being any separate direct effect of the dynamic binding itself. But I will shortly argue that this *ordinary-states-only direct-causation* proposal (or ordinary-states-only proposal, for short) is distinctly disadvantageous compared to to MDyn’s meta-causation proposal.

I do not consider a fourth possibility, namely that it is the spacetime region that (some recent part of) the process history occupies that has a direct causal effect on the state at *t*. One might argue that, in the actual world, for any given region it is determined what the states and dynamic binding within it are, so that mattering of the dynamic binding is implicitly provided by mattering of the region. However, in MDyn theory I wish to allow only some selected aspects of state and binding in a region to be involved in a process occupying that region. In this case, causation by the region is not selective enough.

Another possibility we put aside is that each state of the process has a “minimal trace” of its having been caused through standard state evolution (i.e., without any involvement of meta-dynamism) within the process. Minimal traces have been suggested as part of a mechanism for episodic memory, and are representation-free items somehow linked to past experiences. So, being representation-free, they might avoid problems we have levelled against representation as a mechanism for PRAIS. Presumably one would need to claim that the minimal trace in a give state in the process causally arises from arbitrarily recent states in the process where that causation is partly dependent on the prior states’ own minimal traces. But this is powerless to make the process at the current moment sensitive to (some abutting portion of) its own past history *as a unity* rather than simply sensitive to arbitrarily recent states individually: there is no collecting together of past states into a (sub-)history. The meta-dynamic proposal, on the other hand, is a matter of prior abutting sub-histories dynamically affecting current state as unities. Note, however, that it may be that the effect on current state might be some sort of minimal trace in a new sense (it is some non-representational aspect of of ordinary or dynamism state that is caused by a past region of dynamism). So the issue is not the minimality of minimal traces, but rather how they arise. (Indeed, we might turn the rhetoric around and claim that meta-dynamically produced minimal traces are a good candidate for explaining the occurrence of conscious episodic memories of at least very recent events.)

Of course, we have not proved that there are not, for the purpose of ensuring PRAIS, any conceivable alternatives to the five possibilities we have been considering, namely: representation of histories; direct, meta-dynamic effects of prior dynamism; direct causation by prior states; direct causation by prior region; and minimal traces of ordinary causation. But our proposal stands as at least good candidate explanation of the non-representational mattering of dynamic binding that can be assessed against further candidates if they come to light.

### Appendix A.6. Insecurity of the Ordinary-States-Only Direct-Causation Proposal

The ordinary-states-only proposal in the previous subsection rests on the idea that a sub-trajectory of [ordinary] states over an interval within a process determines the process’s dynamic binding within that interval. But we should immediately elaborate this claim to include any input—incoming dynamic influences from outside the process—that the process has, since the specific nature of a given process includes that input. So the expanded claim is that the state sub-trajectory determines the dynamic binding amongst those states together with incoming dynamism (and outgoing dynamism, i.e., output, but we will not be dwelling on this aspect).

A first set of observations about the proposal are as follows. If it holds that the reality of dynamism is only metaphysical but not physical (whatever sense one can make out of that claim), then we are in the strange position that whether a process has the objective, assumed-to-be *physical* property of being conscious is dependent on some *non*-physical property of state trajectories. On the other hand, if the proposal holds that dynamism is physically real, but does not interact meta-dynamically, then it has a strangely dislocated nature compared to MDyn. It has to separately propose that a state can be directly affected by a prior ordinary-state trajectory, taken as a whole, as an entirely separate matter from the fact that there is dynamism holding the states in the trajectory together. That is, the theory is ignoring the resource that MDyn does use to get the desired sensitivity outcomes, and instead introducing an independent, no less mysterious resource.

The ordinary-states-only direct-causation proposal has a more technical problem, independent of any consideration of what sort of reality it is that dynamism has. What does it mean for the ordinary state sub-trajectory (over an interval within a process) to determine such dynamism? There is more than one possibility. One is that, in the conception of state we have been using, the state sub-trajectory implicitly determines the spacetime region that that part of the process occupies, as it is a function from locations to states. (We separated out the region in our mathematical formulation of a process in Section 3.9, but this was for presentational convenience.) So, we might claim the sub-trajectory determines the internal and incoming dynamism merely in the sense that it determines the dynamism that just *happens to exist* in/into that region and that is relevant to the states in the sub-trajectory. (The relevance qualification is included here because a process may be selective as to the aspects of ordinary state it includes.) Let us call this possibility the *contingent determination* possibility. However, without further assumptions in the proposal, it leaves it completely open that there may be some other pattern of dynamic binding and incoming dynamism that could have been involved with the same state sub-trajectory. So it seems wrong to claim that the specific dynamism in the process *does* matter at all to the process.

This problem is fixed by another version of the proposal, which is the *logically necessary determination* possibility. This is that the ordinary-state sub-trajectory, by strict, objective mathematical derivation, entails that the dynamism in/into the process is exactly such and so (and is therefore equal to the dynamism that is actually in/into the process, if we are talking about a physically possible process). Thus, if the dynamism had been different, the state sub-trajectory would have been different. So the direct effect of merely the states up to but not including *t* on the state at *t* would indirectly make the dynamism in/into that sub-trajectory of states matter to the state at *t*.

However, it is far from clear that it can indeed be maintained that there is always a unique pattern of dynamism in/into the process that is objectively, mathematically implied by its state sub-trajectory, especially if the sub-trajectory does not include all features of state at the spacetime points in question. Perhaps different combinations of internal and incoming dynamism could provide the same sub-trajectory. The theory would have to prove that this could not happen under any circumstances, or at least under circumstances that satisfy any further necessary conditions for consciousness that might have been postulated.

### Appendix A.7. An Auto-Individuation Argument against Ordinary-States-Only

The auto-individuation aspect of PRAIS is that the process should distinguish its inside from its outside by virtue of being sensitive to its own unified process-ness in some prescribed way but not sensitive to the outside world in that way. Here outside means outside the physical region it occupies or outside the particular types of ordinary-state aspect and dynamic aspect included in the process. Adding auto-individuation as a consideration to our current question of how a process’s own dynamism matters to it, we get the requirement that a conscious process, at any given time *t* in its time-span, be sensitive in some prescribed way $W_{d}$ to (some recent) prior dynamism but not sensitive in that way to dynamism outside the process.

Now, the ordinary-states-only proposal needs to give us this distinguishability of inside from outside. Let us grant that, by being directly causally sensitive at any given time to its own (recent) prior state sub-trajectory in some particular way $W_{s}$, the process is thereby sensitive in way $W_{d}$ to the dynamism accounting for that sub-trajectory. The trouble arises when we try to ensure that the process at *t* is *not* sensitive in way $W_{d}$ to dynamism outside the process. For instance, it is not enough for the process at *t* to fail to be sensitive in way $W_{s}$ to states outside the process. This is because the process might be directly causally sensitive to states outside in some way $W_{s}^{\prime }$ that is different from $W_{s}$ but still means the process is sensitive in way $W_{d}$ to dynamism outside. So to get the required selective sensitivity to dynamism, we would have the rather demanding and theoretically troublesome condition that there be no such way $W_{s}^{\prime }$. Or, we could consider the simpler but yet more demanding condition that the process is not sensitive in *any* directly causal way to prior states outside. But this could as a side-effect preclude the process from being sensitive in some way $W_{d}^{\prime }$ to dynamism outside, even though such sensitivity could be useful to include in the theory for reasons unconnected to consciousness. There would then be artefactual restriction that our meta-dynamic proposal does not have.

The problem here for the ordinary-states-only proposal would disappear if the auto-individuation requirement was simply that the process should be sensitive to its own recent prior dynamism in *some* direct way or other, and not sensitive in any such way to prior dynamism outside. Then the corresponding requirement at the level of states seems reasonable. This is the condition that the process is directly causally sensitive to own [ordinary] prior state sub-trajectories in some way or other, but not directly causally sensitive in any way to ordinary state sub-trajectories outside. This state-level requirement is no longer more demanding or complex than the dynamism-level one. However, it would surely be very odd if the presence of consciousness had nothing to do with the *particular* way prior sub-trajectories affect current states.

In sum, while the arguments in this subsection and the previous one are not decisive against the ordinary-states-only direct-causation proposal, they certainly raise important questions for it to answer, and give it a certain artificiality and incoherence in comparison to MDyn. MDyn therefore stands as at least as important a candidate for consideration as the ordinary-states only proposal, and arguably as a more important one.
